# Coral physiology and microbiome dynamics under combined warming and ocean acidification

**DOI:** 10.1371/journal.pone.0191156

**Published:** 2018-01-16

**Authors:** Andréa G. Grottoli, Paula Dalcin Martins, Michael J. Wilkins, Michael D. Johnston, Mark E. Warner, Wei-Jun Cai, Todd F. Melman, Kenneth D. Hoadley, D. Tye Pettay, Stephen Levas, Verena Schoepf

**Affiliations:** 1 School of Earth Sciences, The Ohio State University, Columbus, OH, United States of America; 2 Department of Microbiology, The Ohio State University, Columbus, OH, United States of America; 3 School of Marine Science and Policy, University of Delaware, Lewes, DE, United States of America; 4 Reef Systems Coral Farm, New Albany, OH, United States of America; King Abdullah University of Science and Technology, SAUDI ARABIA

## Abstract

Rising seawater temperature and ocean acidification threaten the survival of coral reefs. The relationship between coral physiology and its microbiome may reveal why some corals are more resilient to these global change conditions. Here, we conducted the first experiment to simultaneously investigate changes in the coral microbiome and coral physiology in response to the dual stress of elevated seawater temperature and ocean acidification expected by the end of this century. Two species of corals, *Acropora millepora* containing the thermally sensitive endosymbiont C21a and *Turbinaria reniformis* containing the thermally tolerant endosymbiont *Symbiodinium trenchi*, were exposed to control (26.5°C and *p*CO_2_ of 364 μatm) and treatment (29.0°C and *p*CO_2_ of 750 μatm) conditions for 24 days, after which we measured the microbial community composition. These microbial findings were interpreted within the context of previously published physiological measurements from the exact same corals in this study (calcification, organic carbon flux, ratio of photosynthesis to respiration, photosystem II maximal efficiency, total lipids, soluble animal protein, soluble animal carbohydrates, soluble algal protein, soluble algal carbohydrate, biomass, endosymbiotic algal density, and chlorophyll *a*). Overall, dually stressed *A*. *millepora* had reduced microbial diversity, experienced large changes in microbial community composition, and experienced dramatic physiological declines in calcification, photosystem II maximal efficiency, and algal carbohydrates. In contrast, the dually stressed coral *T*. *reniformis* experienced a stable and more diverse microbiome community with minimal physiological decline, coupled with very high total energy reserves and particulate organic carbon release rates. Thus, the microbiome changed and microbial diversity decreased in the physiologically sensitive coral with the thermally sensitive endosymbiotic algae but not in the physiologically tolerant coral with the thermally tolerant endosymbiont. Our results confirm recent findings that temperature-stress tolerant corals have a more stable microbiome, and demonstrate for the first time that this is also the case under the dual stresses of ocean warming and acidification. We propose that coral with a stable microbiome are also more physiologically resilient and thus more likely to persist in the future, and shape the coral species diversity of future reef ecosystems.

## Introduction

Increased atmospheric carbon dioxide (CO_2_) is causing the oceans to warm and become more acidic, thus resulting in lower seawater pH and carbonate mineral saturation state. At the current rate of warming and ocean acidification, reefs are expected to experience significant declines in coral abundance, coral diversity, and reef growth before the end of this century [1]. Despite this, some coral species appear to be more tolerant of these predicted conditions than others [2–4]. The relationship between coral physiology and its microbiome may shed light on why some corals are more resilient to global change conditions.

Elevated seawater temperature can lead to coral bleaching: a process whereby scleractinian corals lose substantial numbers of their photosynthetic endosymbiotic dinoflagellates (*Symbiodinium* spp.), giving the colony a pale (hence bleached) appearance. Bleaching damages coral health, slows or arrests growth, and can lead to mortality [1,5–7]. Factors associated with the ability to tolerate and recover from bleaching include coral energy reserves (i.e., lipid, protein, carbohydrates) [8–11], heterotrophic feeding capacity and/or plasticity [12–15], and endosymbiont type shuffling [9,16]. Ocean acidification can also affect some corals. Decreases in seawater pH hamper coral calcification in some species [4,17–19], but not in others [3,4,20,21]. Additionally, ocean acidification can dramatically impact coral health in some cases [2,22], while in other cases it has little or no negative effects on coral host physiology [4,19,23] and is often coupled with a stimulating effect on the endosymbiotic algae [23–25]. However, corals are increasingly exposed to the chronic dual stress of both rising temperature and ocean acidification simultaneously, and our understanding of how corals respond to the single stress of elevated temperature or acidity do not inform us on how coral might respond to the dual stress. At the current rate of atmospheric CO_2_ increase, the tropical oceans are conservatively expected to warm by 1–2°C and seawater pH to decrease by ~0.3 pH units over the course of this century [26].

Coral physiological responses to elevated temperature stress tend to be more severe when simultaneously exposed to reduced pH conditions in some species [2,4,23], but not in other species [3,4,23,24]. Physiological traits associated with coral resilience to both temperature and pH stress include high energy reserves, thermally tolerant endosymbiont types, and heterotrophic feeding on zooplankton or organic matter [4,23,27]. Thus, some species are more resilient to stress conditions expected by the end of this century than others. However, we do not fully understand what drives that resilience. Documented shifts, and lack of shifts, in the microbial community composition of heat-stressed corals [28–32] suggest that the microbiome may be a critical component of coral resilience. More importantly, the relationship between coral physiology and its microbiome may shed light on why some corals are more affected by the dual stress of elevated temperature and ocean acidification than others.

Coral-associated microbial communities are highly diverse, vary seasonally, and–with some exceptions–are generally consistent within a coral species [33–38]. Elevated temperature stress has been associated with significant shifts in coral microbial community composition in some cases [28–30] that appear to return to pre-bleaching states following recovery [28]. Specific Operational Taxonomic Units (OTUs) associated with bacteria of the genus *Vibrio*, and other OTUs from the bacterial classes γ-Proteobacteria, δ-Proteobacteria, and Acidobacteria, coincide with bleaching in corals, suggesting that such taxa cause or facilitate coral bleaching, or that they opportunistically increase in abundance in bleached corals when the coral’s health is compromised [28,29,33,39–44]. Bourne *et al*. [28] hypothesized that the loss of endosymbiotic algae during coral bleaching reduces the amount of reactive oxygen species–natural bacterial inhibitors–thus allowing opportunistic bacteria to infect and/or proliferate. Conversely, Banin *et al*. [33] proposed that bacterial pathology increases with temperature and causes bleaching, which is consistent with studies showing that microbial pathogenesis is temperature-dependent [39]. However, more recent work indicates that the microbiome may play a role in coral resilience to heat stress. Reschef *et al*. [45] proposed that beneficial microbes increase in stressed corals, conferring an immune-like response coined the “Coral Probiotic Hypothesis” making corals tolerant of stressful conditions like bleaching. Santos *et al*. [43] suggested that increases in nitrogen-fixing bacteria in bleached corals provide an alternative mechanism for corals to acquire fixed nitrogen in the absence of abundant endosymbionts, thus helping corals tolerate climate change stresses–though Pogoreutz *et al*. [46] found that increases in Diazotrophs and N-fixation are a stress response that exacerbates coral-algal symbiotic breakdown and bleaching. Others have shown that thermally tolerant corals can also have stable microbial communities under temperature stress or benefit from stable microbial communities that resemble their non-bleached counterparts [31,32]. Therefore, the microbiome has the potential to play a role in coral susceptibility, resistance, and recovery from stress events that could make the difference between coral species resilience and persistence, or extinction.

Ocean acidification has also been shown to cause shifts in coral microbial community composition, though no patterns emerge from the existing literature. Decreases in seawater pH result in increases, decreases, and no change in microbial diversity and relative abundances of the dominant classes of microbes [30,47–50]. Microbially mediated nutrient cycling can also be affected by ocean acidification by causing decreases in nitrogen fixation rates [51]. Clearly, more research is needed to address the effects of ocean acidification on coral microbial communities, particularly in combination with ocean warming.

Today, both rising temperatures and ocean acidification are occurring simultaneously. Only one study to date has examined the combined effects of elevated temperature and ocean acidification on the coral microbiome [30], and none have investigated the possible connection between the coral physiology and the microbiome under these dual stress conditions. Webster *et al*. [30] found that the microbial community composition shifts in response to end-of-century conditions were greater for the coral *Acropora millepora* than for the coral *Seriatopora hystrix*, and that dominant changes in bacterial phyla differed between the species. While *Vibrio* seems to play a large role in coral responses to temperature stress [28,44], this temperature sensitivity is lost when combined with ocean acidification stress [30]. At the same time, how changes in the coral microbiome are related to changes in coral physiology (animal host and endosymbiotic algae) under *both* temperature and pH conditions expected by the end of this century are completely unknown. Understanding this relationship may be key to uncovering why some corals are more resilient than others to climate change (see reviews by [52–54]). Here, we assessed the effects of the *dual stresses* of increased temperature and ocean acidification (i.e., increased *p*CO_2_) expected later this century on coral microbial community composition. We interpreted the microbial findings within the context of previously published physiological measurements from the exact same corals in this study (i.e., animal host and endosymbiotic algae). This holobiont approach to understanding corals [37] can only increase our understanding of why some corals are more resilient than others. We hypothesize that corals with a stable microbial community composition are physiologically more resilient to combined ocean warming and acidification.

## Materials and methods

### Experimental design

This experiment was conducted at Reef Systems Coral Farm (New Albany, OH, USA) in summer 2011 in coarsely filtered (150 μm filters) artificial seawater and is described in detail in Schoepf *et al*. [4]. Additional filtering of the seawater was deemed unnecessary as corals maintain microbial community compositions that are compositionally distinct from their surrounding seawater [32,36,37,55]. Due to financial limitations, this study focused only on a subset of these corals, which were analyzed for microbial community composition. A brief description of the experimental methods that pertain only to this subset of corals is as follows.

Six colonies of the Pacific corals *Acropora millepora* and *Turbinaria reniformis* were collected from northwest Fiji and transported to Reef Systems Coral Farm (New Albany, Ohio, USA) which is a CITES permit holder. The coral colonies were maintained in a single large recirculating tank for 2.5 months prior to the experiment, and were exposed to the same recirculating seawater and environmental conditions. We assumed that the opportunity for acquiring any given bacteria was equal among all corals in the same way that it would have been had the corals been freshly collected from the reef prior to the experiment. Since corals maintain microbial community compositions that are compositionally distinct from the surrounding seawater [32,36,37,55], any differences in the microbial community composition of the corals after 2.5 months in the large tank was assumed to be due to species-specific differences in how coral establish and maintain their microbiome.

Prior to starting the manipulative experiment, 6 control tanks and their shared recirculating sump, and all 6 treatment tanks and their shared recirculating sump were filled with artificial seawater that was made in a common bath and equally partitioned among all tanks and sumps. Thus all seawater starting conditions in the experimental tanks, including the seawater microbial community composition, were the same for all corals in this study and the only differences between the treatment and control tanks during the experiment were the temperature and *p*CO_2_ levels.

Each coral colony was divided into 12 fragments. *A*. *millepora* is a branching coral that contains the thermally sensitive endosymbiont type *Symbiodinium* C21a, while *T*. *reniformis* is a foliose coral that contains the thermally tolerant *Symbiodinium trenchi* (*Symbiodinium* type reported in the companion paper by Hoadley *et al*., [23]). One fragment from each colony was assigned to each of the six control tanks (26.5°C and *p*CO_2_ of 364 μatm) and to each of the treatment tanks (29.0°C and *p*CO_2_ of 750 μatm), yielding a total sample size of 6 treatment and 6 control fragments for each species (total n = 24). This sample size is the same or larger than the sample sizes used in the vast majority of coral microbial studies (e.g., [28,30,32,44,55–57]).

Seawater *p*CO_2_ was controlled by bubbling in pure CO_2_, CO_2_-free air, or ambient air in each sump to achieve the desired *p*CO_2_. Temperature was also controlled in each sump with submerged computer-controlled titanium heaters. At the beginning of the experiment, temperature was raised gradually from 26.5°C to 31.5°C over the first 18 days to prevent heat shock, then maintained at 31.5°C for 6 additional days, for a total of 24 days with an average temperature of 29°C. *p*CO_2_ was raised gradually over the first 4 days to prevent *p*CO_2_ shock, and maintained at 750 μatm for the remaining 18 days. Corals were grown in the tanks for 24 days on a 9:15 hour light:dark cycle (275 μmol quanta m^-2^ s^-1^) and fed every three days with two-day old brine shrimp nauplii that were hatched in a separate single batch culture with new seawater. Coral were fed the brine shrimp in separate feeding containers, and returned to their experimental tanks after one hour. The feeding container water and remaining brine shrimp were discarded so as not to introduce brine shrimp into the recirculating system (see Schoepf *et al*., [4] for more details). The control temperature of 26.5°C represented the average summer temperature in Fiji where the coral colonies were originally sourced, while the average elevated temperature of 29.0°C represented the upper limit of current Fiji summer temperatures, but is still below the bleaching threshold at that location (www.ospo.noaa.gov/Products/ocean/index.html), and representative of the increase in baseline temperatures expected by the end of this century in tropical regions under the RCP 8.5 scenario [26]. The control and treatment *p*CO_2_ levels represented present day conditions and those expected by mid-century under the RCP 8.5 scenario and by 2100 under the RCP 6.0 scenario [26], respectively. Throughout the study, temperature, salinity, pH_NBS_, and total alkalinity (TA) were measured daily according to methods described in Schoepf *et al*. [4]. Daily *p*CO_2_, aragonite saturation state (Ω_arag_), and pH (reported on the pH total scale, pH_T_) were then calculated according to Schoepf *et al*. [4]. The experiment ran for 24 days from 19 July– 12 August, 2011, then coral fragments were frozen at -80°C, and transported to the lab for analyses.

### 16S rRNA gene sequencing and OTU table construction

Whole coral tissue and associated mucus layer was airbrushed with milliQ water to produce a slurry from the 24 frozen coral fragments and frozen at -80°C. Coral slurries were centrifuged and total genomic DNA was extracted using PowerSoil® DNA Isolation kits according to the manufacturer instructions (MoBio Laboratories, Inc., Carlsbad, CA, USA). DNA was eluted in the provided buffer and quantified on a Qubit 2.0 instrument (Invitrogen, Carlsbad, CA, USA). DNA samples were then sequenced at the Argonne National Laboratory using the Illumina MiSeq platform. The V4 region of the 16S rRNA gene was targeted with the universal primers 515F and 806R [58,59] that targeted both bacteria and archaea. The use of the 16S rRNA gene is a standard in the field for identifying microbial community composition and structure in the coral literature (e.g., [28,31,32,44,49,55,57]. Sequencing data was processed using the QIIME platform [60]. Briefly, FASTQ forward and reverse files were joined and libraries were split based on the barcodes. Sequence length control and end-trimming were performed with default parameters, and the minimum quality score was set as 19. Additional parameters included a minimum count of 10 for an OTU to be retained and presence in at least 25% of the samples. OTUs were assigned based on the release 111 of the Silva ribosomal database [61] and clustered at 97% similarity level. Chimeras were identified and removed with usearch61 [62]. After the OTU table was generated, any OTUs matching mitochondria, chloroplasts, or eukaryotes were removed from the biom table using filter_taxa_from_otu_table.py and later summarize_taxa.py. Details on PCR, sequence processing, and command line work are available in the S1 Methods of the Supporting Information.

### Physiology measurements

Physiological measurements in this study were already reported in Schoepf *et al*. [4], Levas *et al*. [27], and Hoadley *et al*. [23] along with details of each analysis. In brief, calcification [from [4]] was measured by the buoyant weight method [63] during the study and standardized to surface area to produce calcification rates for the first and second half of the experiment. Only the latter calcification rate is used in this study as it is most relevant to the other physiological and microbial data, which were all measured either during the last few days of the experiment or at the end of the 24-day experiment. Particulate organic carbon (POC) flux [from [27]], ratio of photosynthesis to respiration (P:R) [from [23]], and photosystem II maximal efficiency (F_v_/F_m_) [from [23]] were measured on living corals during the last four days of the experiment. POC flux was calculated as the difference in blank-corrected POC concentration of the seawater in a sealed chamber containing a coral fragment and the initial seawater POC concentration, standardized to the incubation duration (1.5 hours) and fragment surface area. POC was defined as the concentration of organic particles captured on a GF/F (0.7 μm nominal pore size) and measured by combustion using a Costech Elemental Analyzer. When POC fluxes are positive, corals are releasing organic matter into the water, typically in the form of mucus. When POC fluxes are negative, coral are taking up POC as a source of fixed carbon [27]. Maximum net photosynthetic rate (P) and light acclimated dark respiration (R) were measured from the change in oxygen concentration of each coral fragment in a respirometry chamber and standardized to surface area. The ratio of P:R was calculated from the ratio of gross P (net P + R) to R. Dark acclimated quantum yield of photosystem II (F_v_/F_m_), was measured in the light by pulse amplitude modulation fluorometry (Diving PAM, Waltz, Germany). F_v_/F_m_ is generally viewed as a proxy for coral heat sensitivity [64].

In the laboratory, the following physiological analyses were performed on all frozen coral fragments collected on the last day of the experiment and reported by Schoepf *et al*. [4]: total lipids, animal host soluble protein, animal host soluble carbohydrates, total biomass, endosymbiotic algal density, chlorophyll *a*, and total surface area. The following physiological variables were performed on the same frozen fragments and reported by Hoadley *et al*. [23]: soluble algal protein, and soluble algal carbohydrates.

In brief, total lipids, animal host soluble protein and animal host soluble carbohydrates were quantified from ground whole coral subsamples and standardized to ash-free dry weight. Lipids were extracted in a 2:1 chloroform:methanol solution, washed in 0.88% KCl, extracted and washed again in 100% chloroform and 0.88% KCl, respectively. For the animal host soluble protein and carbohydrate analyses, the animal fraction was separated from the endosymbiotic algae via sonication and centrifugation. Protein was extracted using the bicinchoninic method [65] with bovine serum albumin as a standard (Pierce BCA Protein Assay Kit). Carbohydrates were quantified using the phenol-sulfuric acid method with glucose as a standard [66]. Biomass was determined as the difference between the dry and burned weight of a ground coral subsample and standardized to surface area. Coral surface area was determined using the single wax dipping method [67,68] for the branching *A*. *millepora* and the aluminium foil method [69] for the plating *T*. *reniformis*.

Endosymbiotic algal density, chlorophyll *a*, soluble protein, and soluble carbohydrates were determined on airbrushed coral slurry where the algae were then separated from the coral host via centrifugation, and standardized to surface area. The number of algal cells were counted on six replicate subsamples using a hemocytometer and a Nikon microphot-FXA epifluorescent microscope. Chlorophyll *a* was extracted from another subsample in methanol and quantified spectrophotometrically according to Porra *et al*. [70]. Soluble algal protein and carbohydrates were determined using the same methods as above for the animal host.

### Statistical analyses

The data were analyzed using non-parametric multivariate techniques to determine if the microbial community composition and structure varied among coral species and treatments. As rare OTUs can sometimes play an interesting role in microbial ecology [65], we included all sequences in the analyses. The OTU table was used as the input for microbial community analyses in PRIMER E v. 1.0.6 (Quest Research Limited, Auckland, New Zealand). The sequence data was evaluated at three levels: Phylum, Class, and OTU. These levels of analyses were done to facilitate comparisons with other publications. At the phylum level (Level 2 table from Qiime), the sum of the abundances of all OTUs within each phylum was square root-transformed prior to construction of a Bray-Curtis resemblance matrix. The same was performed at the Class (Level 4) level. Analysis of Similarity (ANOSIM) was used to test for the effect of species and treatment on the microbial community composition at the phylum and class levels. Similarity Percentage (SIMPER) analyses were conducted to determine the degree of dissimilarity in microbial communities between coral species, between control and treatment corals within a species, and to determine which phyla or classes were responsible for the largest portion of those dissimilarities. The same analyses were then conducted using all of the individual OTU sequence abundances. In addition, the Shannon Diversity Index at the OTU level was computed for each species and treatment. A univariate two-way analysis of variance (ANOVA) was used to test the effects of species and treatment on the Shannon Diversity Index, where the data was first tested for normality using the Shapiro-Wilk’s test and homogeneity of variance was assessed with plots of expected vs. residual values. A posteriori Slice tests (i.e., tests of simple effects, Winer [71]) were used to determine if treatment corals differed from controls within each species.

The physiological measurements were analyzed using non-parametric techniques to determine if corals differed physiologically between species and treatments. Since these data are a subset of those from Schoepf *et al*. [4], Hoadley *et al*. [23], and Levas *et al*. [27], and presented together for the first time, new statistical analyses were necessary using only the data from the samples in this study. Univariate Kruskal-Wallis tests were conducted on each individual variable to determine if they differed between control and treatment corals of each species. A Euclidean distance-based resemblance matrix was then constructed using normalized data. ANOSIM was used to test for the effect of species and treatment on the overall coral physiology. SIMPER analyses were conducted to determine which physiological variable(s) were responsible for the largest portion of the differences detected in the ANOSIM. These multi-variate analyses of the physiological data are unique to the current study.

Non-parametric multidimensional scaling (NMDS) analyses were performed to graphically represent relationships between the microbial abundance variability and the coral physiology variability between species and treatments in multidimensional space. The two sets of data (microbial and coral physiology) were then compared to determine if microbial community composition and structure co-varied with coral physiology using two strategies: 1) vectors were added to NMDS plots (Pearson correlations > 0.1) to show the direction and magnitude of the influence of each physiological variable to the distribution of the microbial-based data points in 2D NMDS space, and 2) Spearman correlations were performed to compare the physiology-based NMDS distribution pattern to the microbial-based NMDS distribution pattern (BEST test).

The Kruskal-Wallis tests were performed using SAS software version 9.2. All other analyses were generated using the software package Primer V6 [72,73]. Where appropriate, p < 0.05 was considered significant.

Since the coral fragments in the control and treatment tanks were all from the same parent colonies (i.e., one fragment from each colony and species in each tank), changes in the microbiome were due to treatment effects and not due to genetic differences between colonies. Overall, the experimental design allowed us to detect changes in the coral microbial community composition due to treatment effects, independent of starting microbial community composition and inter-colony variability. Thus, any observed differences in the coral microbial community composition in the experiment were due to innate differences between species and treatment effects alone.

## Results

**Fig 1:**
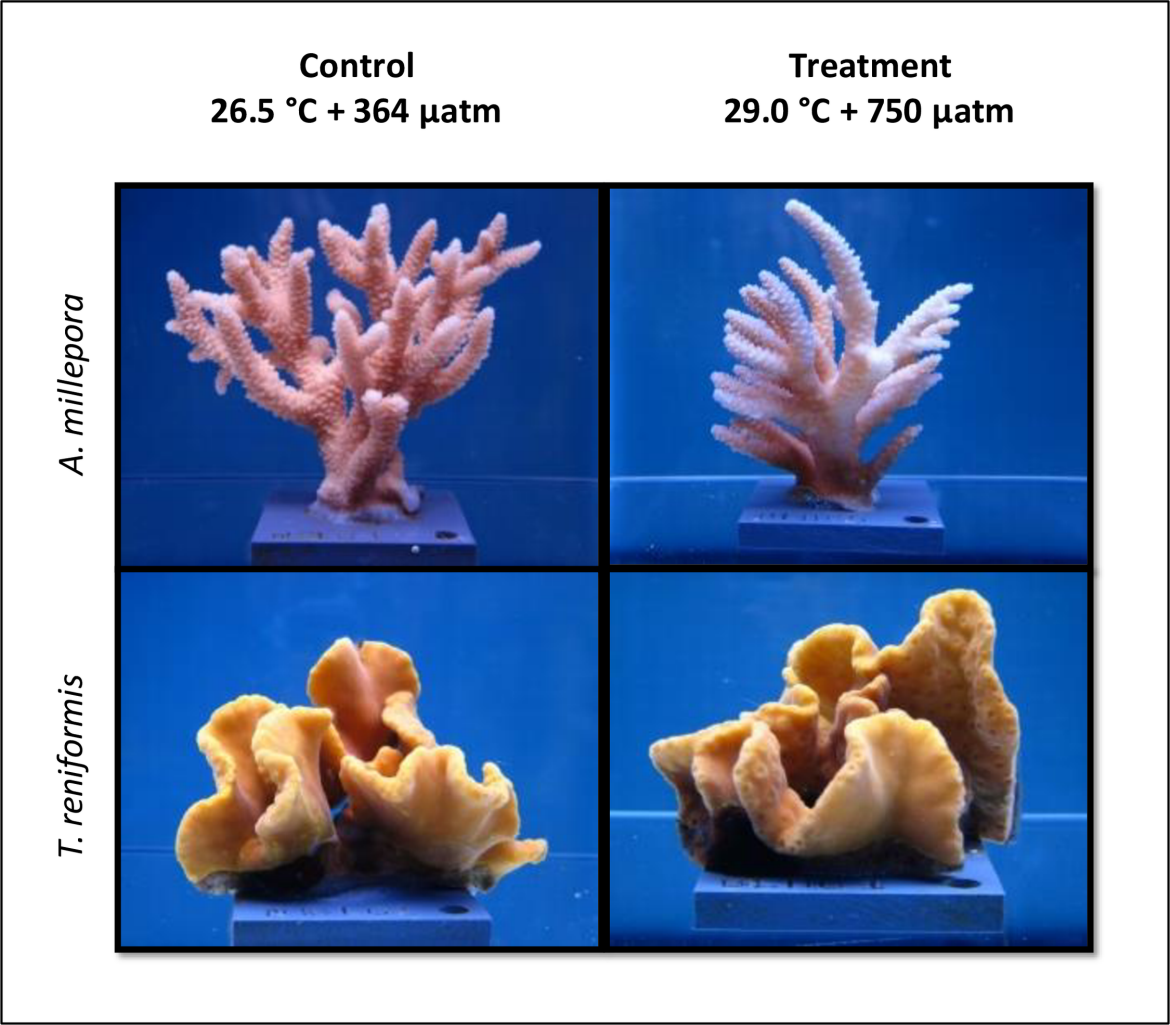
Photographs of representative coral fragments at the end of the experiment. *A*. *millepora* and *T*. *reniformis* after 24 days in the experiment under control (26.5°C and *p*CO_2_ of 364 μatm) (left side) and treatment (29.0°C and *p*CO_2_ of 750 μatm) (right side) conditions. The treatment *A*. *millepora* fragment typifies the paling that was observed in half of the fragments in this group. Photos by V Schoepf.

All corals survived the experiment, though their physical appearance varied by species and treatment. Half of the treatment *A*. *millepora* were pale at the end of the study and all other fragments retained a healthy brown (for control *A*. *millepora*) or mustard yellow (for both control and treatment *T*. *reniformis*) color (Fig 1). The average seawater temperature, pH_T_, *p*CO_2_, total alkalinity, and Ω_arag_ for the control and treatment tanks are given in Table 1.

**Table 1:** Average conditions (± 1standard error) for the control and treatment tanks.

	Control	Treatment
Temperature (°C)	26.45 ± 0.01	28.93 ± 0.02
pH_T_	8.07 ± 0.01	7.81 ± 0.01
*p*CO_2_ (μatm)	364 ± 10	750 ± 26
TA (μmol kg^-1^)	2269 ± 11	2305 ± 9
Ω_arag_	3.69 ± 0.07	2.52 ± 0.06

pH_T_ = pH reported on the total scale, TA = total alkalinity, Ω_arag_ = aragonite saturation state.

Modified from Schoepf et al. [4].

### Microbial community composition

**Fig 2:**
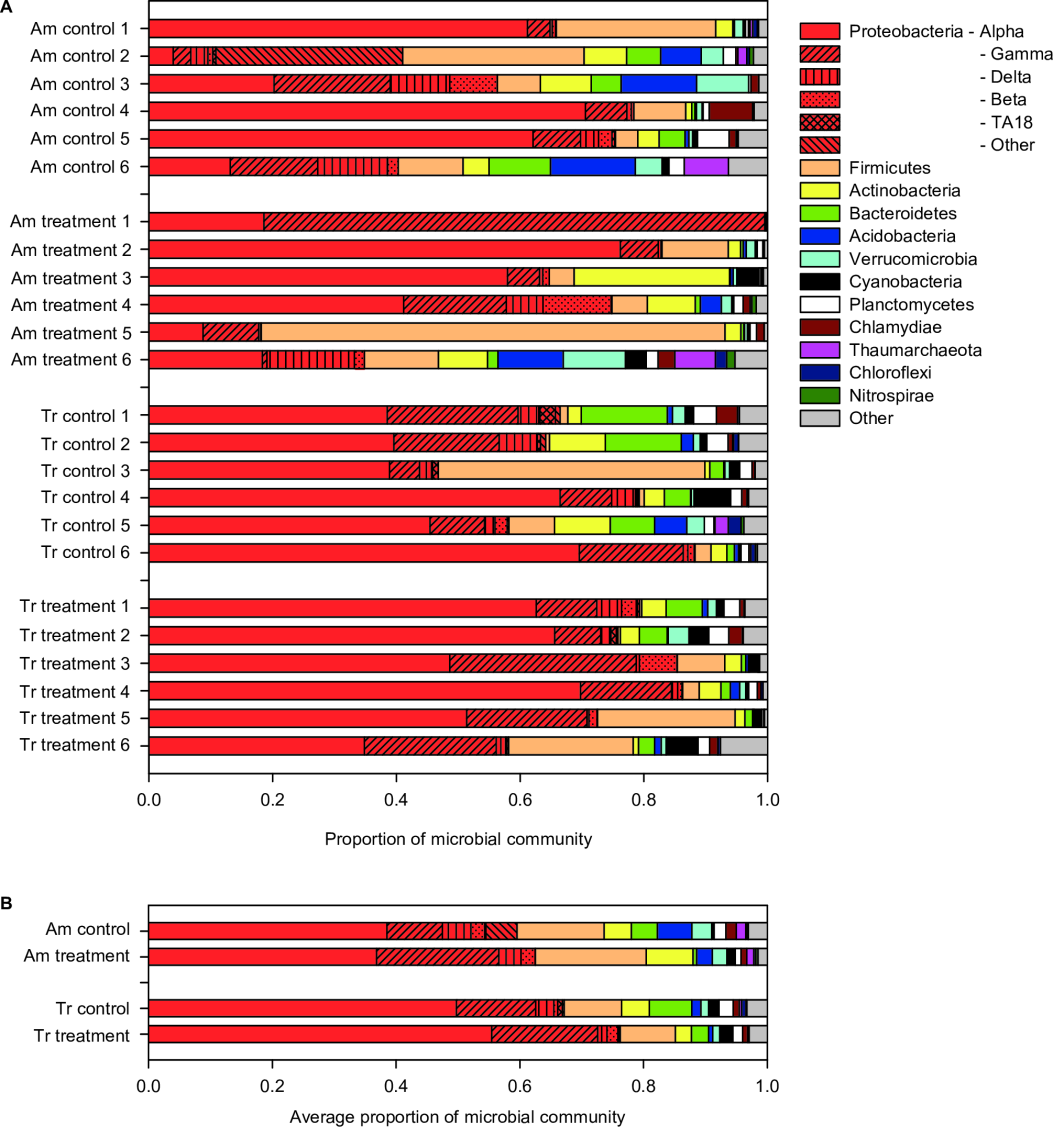
Microbial relative abundances by phylum. (A) *Acropora millepora* (Am) and *Turbinaria reniformis* (Tr) under control (26.5°C and *p*CO_2_ of 364 μatm) and treatment (29.0°C and *p*CO_2_ of 750 μatm) conditions for each sample (1–6). (B) Average microbial relative abundances by Phylum.

Overall, there were 831 OTUs across all coral fragments spanning 48 Phyla: 565 for *A*. *millepora* and 650 for *T*. *reniformis* (S1 Table). In addition, 388 OTUs were shared by both coral species, 175 were unique to *A*. *millepora*, 263 were unique to *T*. *reniformis*. We observed that sequences affiliated with members of the Proteobacteria (primarily Alphaproteobacteria and Gammaproteobacteria classes) and Firmicutes Phyla were the most abundant in treatment and controls of both coral species, with sequences matching Actinobacteria, Bacteroidetes, and Acidobacteria Phyla being the next most abundant and typically present in both species and treatments (Fig 2A). However, ANOSIM revealed that at the Phylum level, bacterial communities on average did not significantly differ between species, or between treatment and controls of either species (ANOSIMs Global R -0.036, p = 0.66) (Fig 2B).

**Fig 3:**
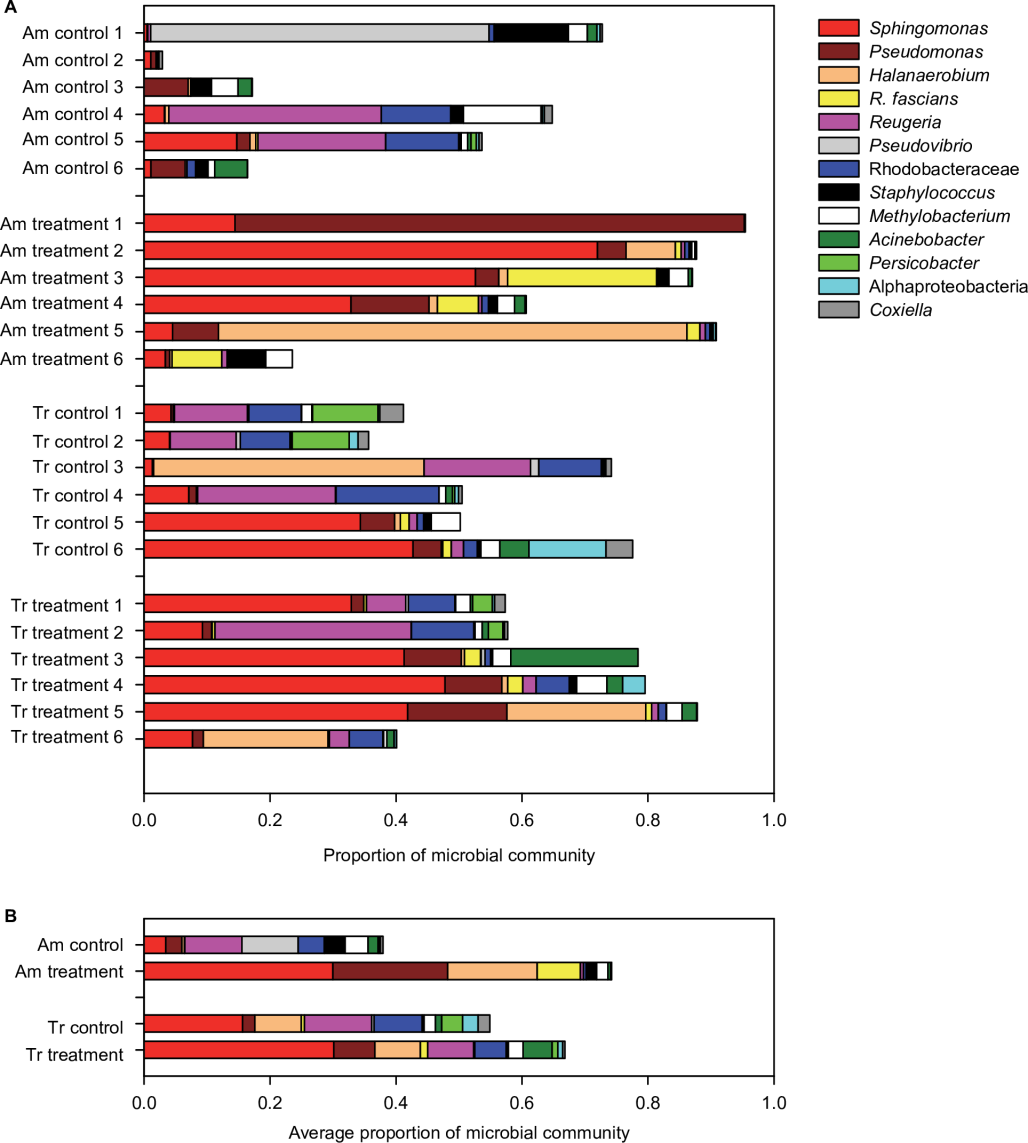
Relative abundance of the nine OTUs contributing the most to the dissimilarity between treatment and control corals as determined by SIMPER analysis. (A) Results for *Acropora millepora* (Am) and *Turbinaria reniformis* (Tr) under control (26.5°C and *p*CO_2_ of 364 μatm) and treatment (29.0°C and *p*CO_2_ of 750 μatm) conditions for (A) each sample and (B) on average per treatment and species. Average relative abundance details in S2 Table. Dissimilarity analyses details by SIMPER are in S2 Table. ANOSIM results in Table 2 correspond to panel (B). Note that the proportionate contributions of the OTUs here do not sum to 1 because only the nine OTUs contributing the most to the dissimilarities are shown. *Sphingomonas*, *Ruegeria*, *Pseudovibrio*, *Methylobacterium*, and Rhodobacteraceae belong to the class Alphaproteobacteria. *Pseudomonas*, *Acinetobacter*, and *Coxiella* belong to the class Gammaproteobacteria. *Rhodococcus fascians* belongs to the phylum Actinobacteria, *Halanaerobium* and *Staphylococcus* to Firmicutes, and *Percinobacter* to Bacteroidetes.

Significant differences in the coral microbial communities were found at the Class and OTU levels between the species, and between treatment and controls of *A*. *millepora*, but not between treatment and control *T*. *reniformis* according to ANOSIM analyses (Table 2, S1 Fig). At the OTU level, the microbial community composition of treatment and control *A*. *millepora* and *T*. *reniformis* were 77% and 59% dissimilar, respectively (S2 Table). In *A*. *millepora*, this dramatic dissimilarity was due to increases in OTUs associated with *Sphingomonas*, *Pseudomonas*, and *Halanaerobium*, the virtual appearance of *Rhodococcus fascians*, and decreases in 16S rRNA gene sequence associated with the Rhodobacteraceae in treatment corals compared to the controls (Fig 3, S2 Table). The virtual disappearance of *Pseudovibrio* was due to a single coral fragment (Fig 3, S2 Table). At the same time, the Shannon Diversity Index decreased significantly from an average of 3.21 in control to 1.98 in treatment *A*. *millepora* (S3 Table). Though variability among treatment and control *A*. *millepora* fragments was high (Fig 3A), several overarching patterns emerged in this coral species. Treatment conditions resulted in relative abundance increases in sequences affiliated with *Sphingomonas* (observed in 5 of the 6 fragments), *Pseudomonas* (observed in 4 of the 6 fragments), *Halenaerobium* (observed in 5 of 6 fragments) and *R*. *fascians* (observed in all 6 fragments) coupled with decreases in a 16S rRNA gene sequence associated with the Rhodobacteraceae (observed in 4 of 6 fragments) (Fig 3A, S1 Table). On average, this resulted in -9 to +100-fold changes in these OTUs (Fig 3B). Microbial OTU diversity did not significantly differ between control and treatment *T*. *reniformis* (average Shannon Diversity Index of 3.4 vs 2.9, respectively) nor between species (S3 Table).

**Table 2:** One-way ANOSIMs of microbial community composition with pairwise tests of each coral species and treatment combination at the a) Class and b) OTU level.

Pairwise tests of groups	R statistic	P-value
**a) Class level**		
Am Control vs. Am Treatment	0.296	**0.028**
Am Control vs. Tr Control	0.254	**0.043**
Am Control vs. Tr Treatment	0.433	**0.002**
Am Treatment vs. Tr Control	0.356	**0.013**
Am Treatment vs. Tr Treatment	0.230	**0.002**
Tr Control vs. Tr Treatment	0.039	0.284
**b) OTU level**		
Am Control vs. Am Treatment	0.220	**0.048**
Am Control vs. Tr Control	0.263	**0.037**
Am Control vs. Tr Treatment	0.394	**0.009**
Am Treatment vs. Tr Control	0.426	**0.011**
Am Treatment vs. Tr Treatment	0.302	**0.004**
Tr Control vs. Tr Treatment	0.059	0.234

The overall model at the Class and OTU levels were significant (Global R = 0.256, p<0.01, 999 permutations and R = 0.257, p<0.02, 999 permutations, respectively). Bolded p-values are significant. Am = Acropora millepora, Tr = Turbinaria reniformis, Control = 26.5°C and pCO_2_ of 364 μatm, Treatment = 29.0°C and pCO_2_ of 750 μatm.

### Coral physiology

**Fig 4:**
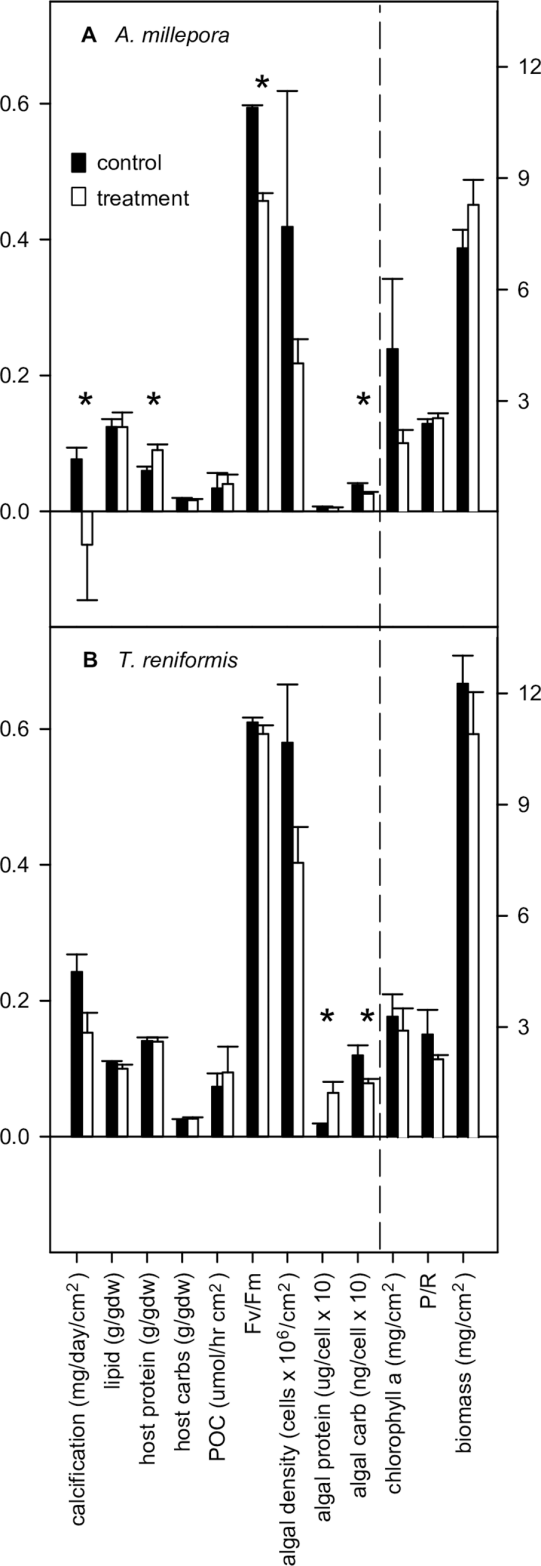
Average (± 1SE) physiological variables. Results given for (A) *Acropora millepora* and (B) *Turbinaria reniformis*. Measurement units for each variable in brackets along the x-axis. Left-hand y-axis scale corresponds to the first 9 variables and the right-hand y-axis scale corresponds to the last three variables (separated by a dashed line). Black bars = control coral (26.5°C and *p*CO_2_ of 364 μatm), white bars = treatment coral (29.0°C and *p*CO_2_ of 750 μatm), carbs = carbohydrates, POC = particulate organic carbon flux, * = significant difference between control and treatment averages for a given variable by Kruskal-Wallis test (details in S3 Table). Data from Schoepf *et al*. [4], Hoadley *et al*. [23], and Levas *et al*. [27].

Calcification, F_v_/F_m_, and algal carbohydrate concentration significantly declined by 136%, 23%, and 32%, respectively, while animal host protein concentration increased by 51% in treatment compared to control *A*. *millepora* (Fig 4A, S4 Table). In *T*. *reniformis*, the endosymbiotic algal protein was four-fold higher and algal carbohydrate concentrations were 34% lower in the treatment compared to control corals (Fig 4B, S4 Table). No other significant differences were detected between treatment and control corals of either species (S4 Table). It is worth noting that host protein concentrations as well as the total energy reserves (i.e., lipid, protein, and carbohydrates) in treatment *T*. *reniformis* were 100% and 13% greater than in treatment *A*. *millepora*, respectively (Fig 4). When all of the physiology data were evaluated as a whole, significant overall differences between coral species, and between treatments within species, were found (Table 3). Animal host protein and endosymbiotic algal carbohydrates contributed the most, and algal cell density and P:R the least, to the overall physiological differences between the two coral species (S5 Table).

**Table 3:** One-way ANOSIM of coral physiology with pairwise tests of each species and treatment combination.

Pairwise tests of groups	R statistic	P-value
Am Control vs. Am Treatment	0.265	0.015
Am Control vs. Tr Control	0.515	**0.002**
Am Control vs. Tr Treatment	0.537	**0.002**
Am Treatment vs. Tr Control	0.807	**0.002**
Am Treatment vs. Tr Treatment	0.754	**0.002**
Tr Control vs. Tr Treatment	0.178	0.024

The overall model was significant (Global R = 0.509, p<0.001, with 999 permutations). Bolded p-values are significant. Am = Acropora millepora, Tr = Turbinaria reniformis, Control = 26.5°C and pCO_2_ of 364 μatm, Treatment = 29.0°C and pCO_2_ of 750 μatm.

### Coral microbiome and physiology

**Fig 5:**
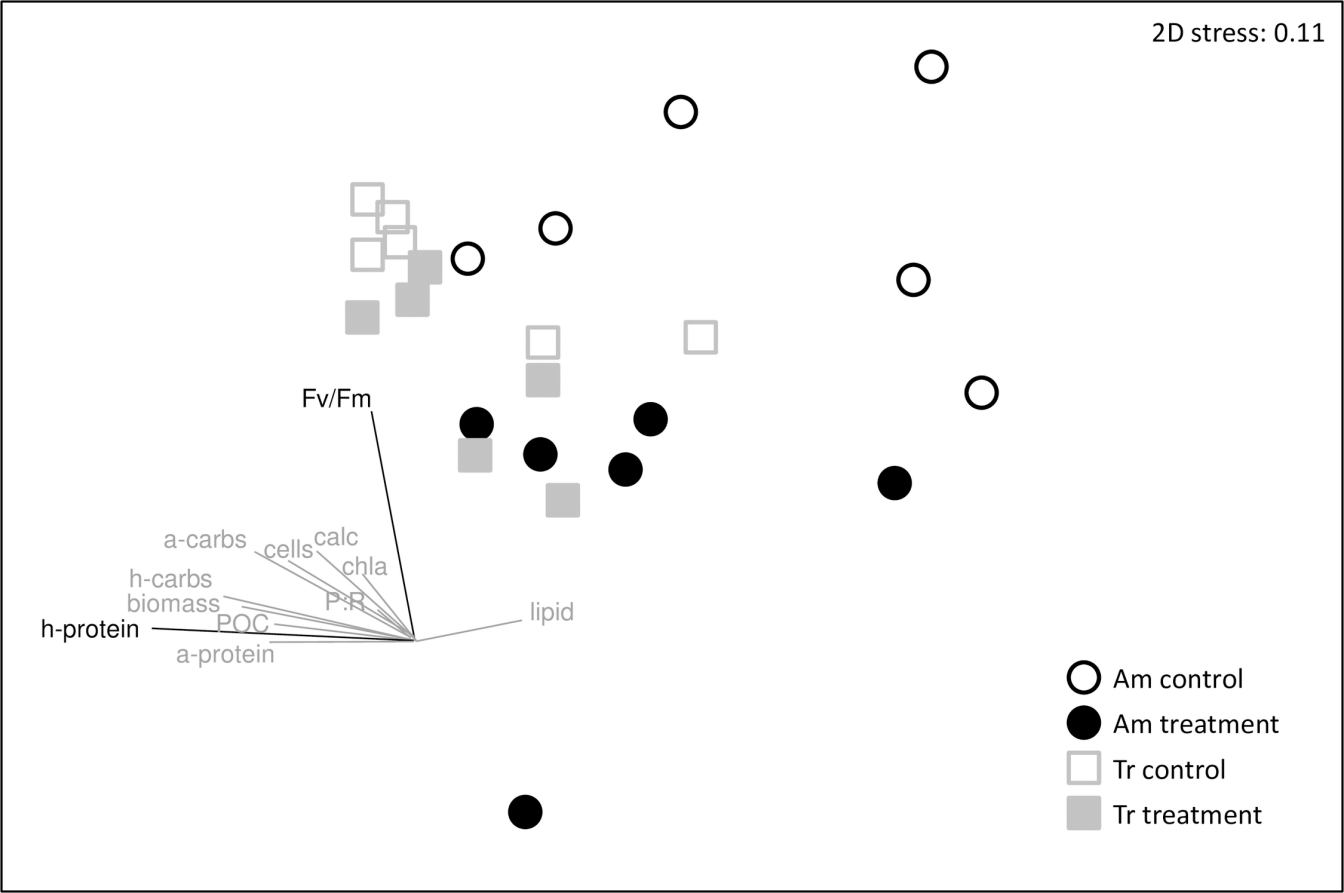
NMDS ordination using microbial OTU community composition from all 24 coral fragments. Gray vector overlay shows the proportional influence of each physiology variable to the NMDS plot distribution. Am = *Acropora millepora* (circles), Tr = *Turbinaria reniformis* (open squares), Control (blue) = 26.5°C and *p*CO_2_ of 364 μatm, Treatment (black) = 29.0°C and *p*CO_2_ of 750 μatm. Calc = calcification rate during the last two weeks of the study, a_cells = endosymbiotic algal cell density, Chla = chlorophyll *a* concentration, carbs = carbohydrate concentration, lipid = total lipid concentration, protein = soluble protein concentration, POC = particulate organic carbon flux, h = animal host, a = endosymbiotic algae.

NMDS analyses of the microbial OTU relative abundance data revealed clear separation of the coral microbial communities between the coral species, and between treatment and controls of *A*. *millepora*, but not between treatment and control *T*. *reniformis* (Fig 5, Table 2). Of the physiological variables, host protein and F_v_/F_m_ best described the overall microbiology NMDS pattern of the corals (BEST, spearman correlation R = 0.344, p = 0.26), though the results were not statistically significant. Nevertheless, the host protein vector appears to define an axis that best describes differences in the microbial communities between the species whereas the F_v_/F_m_ vector appear to define an axis separating the microbial communities of treatment and control corals (Fig 5). BEST analysis was also conducted on *A*. *millepora* alone as it expressed significantly different microbial communities between treatment and control fragments. However, the model was not significant and no physiological variables described the overall microbiology NMDS pattern within *A*. *millepora* (Global R = 0.21, p = 0.95).

## Discussion

When corals are exposed to stress such as increased temperature or decreases in seawater pH, the coral microbial community often undergoes compositional changes [28,29,40,44,48,49,55,74,75]. The current study is the first to simultaneously examine the coral microbial community composition, coral host physiology, and endosymbiotic algal physiology associated with the dual stress of increased seawater temperature and lower pH. When comparing the two coral species here, we find that *T*. *reniformis* with its thermally tolerant endosymbiont *Symbiodinium trenchi* has a stable microbial community composition and is only slightly affected physiologically when exposed to conditions expected by the end of this century for 24 days, whereas *A*. *millepora* with its more thermally sensitive endosymbiont type C21a suffers both a decline in microbial diversity and a shift in its microbial community composition combined with a more dramatic physiological decline under the same stressful conditions.

The coral microbiomes of *A*. *millepora* and *T*. *reniformis* were dominated by members of the phylum Proteobacteria (Fig 2), which is a pattern found in most coral microbial communities (e.g., [30,48,55,76]). However, when exposed to the dual stress of increased temperature and lower pH, we found no significant change in the phylum-level microbial community composition of *A*. *millepora* (Fig 2B) whereas Webster *et al*. [30] did. This could be due to the different experimental conditions of both studies. Namely, Webster *et al*. [30] corals were collected two weeks before the experiment, maintained at 28°C or 31°C during the experiment, and were kept in 1μm filtered seawater and starved of any heterotrophic food source. In the current study, the corals were collected three months prior to the experiment, maintained at 26.5°C or 29°C in 150 μm filtered seawater, and fed *Artemia* nauplii twice a week.

At the OTU level, the microbial community composition changed and diversity decreased for *A*. *millepora*, but not *T*. *reniformis*, when exposed to the dual stress treatment (Table 2, S3 Table). In the field of ecology, the insurance hypothesis states that microbial diversity stabilizes microbial community function [77]. Therefore, the loss of diversity observed in dually stressed *A*. *millepora* may indicate a destabilized microbial community function. However, increases in coral microbial diversity in response to sewage and sedimentation stress have also been interpreted to mean microbial community destabilization [78]. Here, we find that the large shifts in OTUs most responsible for the differences between treatment and control *A*. *millepora* corals do suggest an association between the microbiome and decreased coral health, which suggests destabilized microbial function (Fig 3). Specifically, the large increases in OTU relative abundance of *Sphingomonas* and *Pseudomonas* (Fig 3B) may be an indication of declining coral health as *Sphingomonas*-like bacteria and *P*. *aeruginosa* are associated with coral disease [44,79]. The large declines in Rhodobacteraceae could indicate a decrease in nitrogen-fixing ability [43,51,80]. Lastly, the known plant pathogen *Rhodococcus fascians* dramatically increased in all six *A*. *millepora* fragments (Fig 3A). Though we have no direct evidence that any of the bacterial shifts in *A*. *millepora* led to specific diseases or health decline, none of these OTU shifts suggest an acclimation response that could be conferring a probiotic [45] or protective effect in response to the dual stress of elevated temperature and ocean acidification. Alternatively, these large OTU shifts may indicate a restructuring of the microbial community in order to facilitate adaptation to the dual-stress conditions [31]. The maintenance of diversity and the microbial community stability observed in *T*. *reniformis* might have been because this species was already pre-adapted to the dual stress conditions, as has been demonstrated for some populations of *Acropora hyacinthus* from American Samoa [31]. However, it is unlikely that the microbial community of *T*. *reniformis* was already pre-adapted to the dual stress of elevated temperature and *p*CO_2_ while *A*. *millepora* was not, since both sets of coral colonies were collected from the same site with the same temperature and pH history. Additional studies in other coral species from other regions are needed to further evaluate these findings.

More importantly, the combined microbial and physiological changes in *A*. *millepora* indicate that this species was broadly compromised under treatment conditions. Major components of the holobiont–the animal host, endosymbiotic alga, and the microbiome–all deteriorated under treatment conditions. In addition to the decline in microbial diversity and shifts in microbial community composition (Fig 3), decreases in calcification, F_v_/F_m_, and algal carbohydrate energy reserves in *A*. *millepora* indicate that both the host and algal physiological functions were compromised under treatment conditions (Fig 4A). In contrast, the *T*. *reniformis* holobiont was less affected with no negative impacts on the coral host physiology, a decline only in the algal carbohydrates, and no significant changes in the microbiome community or diversity (Fig 4B, Table 2, S3 Table). Though overall physiology was significantly different between treatment and controls for both species, the degree of separation between the two groups was greater for *A*. *millepora* than for *T*. *reniformis* (Table 3). In addition, treatment *T*. *reniformis* had double the host protein concentrations and slightly greater total energy reserves (i.e., lipid, protein, and carbohydrates) compared to treatment *A*. *millepora* (Fig 4). High energy reserves have been shown to be a key component to coral resilience in the face of temperature stress [8,9]. Interestingly, host animal protein was one of two variables that explained the highest amount of variation in microbial community composition (Fig 5) and overall physiological differences between the two species (S5 Table). In addition, *T*. *reniformis* releases almost twice as much organic matter (probably as mucus) as *A*. *millepora* under control and treatment conditions (Fig 4) [27], which could provide a stable medium for cultivating its microbiome since bacterial growth is dramatically enhanced on coral mucus [81,82]. This could be advantageous as microbial community composition stability has been linked to coral health in some cases [31,32,81]. It is also possible that *T*. *reniformis* has greater heterotrophic capacity and/or plasticity under dual stress conditions than does *A*. *millepora*. Previous work has shown that corals that are either heterotrophically plastic or have high heterotrophic capacity recover more quickly from temperature stress [12,14]. Heterotrophic plasticity or high capacity under the dual stress of elevated temperature and *p*CO_2_ could potentially underlie some of the holobiont resilience observed here in *T*. *reniformis*, though further research is needed to test this hypothesis. Finally, *T*. *reniformis* hosted a thermally tolerant endosymbiont type *Symbiodinium trenchi* and maintained F_v_/F_m_ under dual stress, whereas *A*. *millepora* hosted the more sensitive endosymbiont C21a and F_v_/F_m_ rates dropped (Fig 4). While endosymbiont type does play a role in thermal sensitivity, *T*. *reniformis* also has a much thicker tissue layer than *A*. *millepora*, which would provide greater photoprotection to its *S*. *trenchi* endosymbionts when under stress [23]. At this time, it is not clear what the link between endosymbiont type and microbial community composition shifts (or stability) under a dual temperature and acidity stress is. What does emerge from this study is that the physiological traits of *T*. *reniformis*, combined with a thermally tolerant *Symbiodinium* type and stable microbiome together appear to make it more tolerant to warmer and more acidic seawater conditions than *A*. *millepora*.

### Summary

Overall, we show that the bacterial microbiome changed in the physiologically sensitive coral *A*. *millepora* but not in the physiologically more tolerant coral *T*. *reniformis* in response to conditions of elevated temperature and OA expected later this century. These findings are consistent with previous physiological work on the coral host and endosymbiotic algae of these species demonstrating the more resilient nature of the *T*. *reniformis* holobiont compared to the *A*. *millepora* holobiont [4,23,27]. These findings support the hypothesis that coral with a stable and diverse microbiome are also physiologically more resilient to the dual stress of elevated temperature and OA. Furthermore, our findings suggest that the animal host may play a role in determining the microbial community composition and diversity, and that multiple traits across the holobiont (i.e., host energy reserves, mucus production, *Symbiodinium* type, microbiome stability) appear to be involved in coral sensitivity or resilience to changes in seawater conditions expected on reefs later this century. We hypothesize that coral holobiont deterioration in the face of climate change may be in part triggered by a combination of the animal host’s protein and mucus influence on the microbiome and the *Symbiodinium* type. Further research is needed to determine if shifts in the coral holobiont physiology and/or *Symbiodinium* type cause shifts in the microbiome or vice versa, or if the two are independent of each other. Since some coral diseases and bleaching are known to be caused by bacteria [39,44,83,84], it is unlikely that the microbiome community changes in response to environmental stress are independent of the coral physiology. We further suggest that large organic matter release rates (typically in the form of mucus) may be important for supporting stable and presumably healthier microbial symbionts for corals. Our results are consistent with recent findings that some temperature-stress tolerant corals have a stable microbiome or benefit from stable microbial communities that resemble their non-bleached counterparts [31,32], and demonstrate for the first time that this also appears to be the case under the dual stresses of ocean warming and acidification. Additional studies with different coral species are needed to fully test this hypothesis. Model projections of coral persistence over the next century might need to consider not just coral host and endosymbiotic algae physiological responses to stress, but the combined responses of the coral host, endosymbiotic algae, and microbiome.

## Supporting information

S1 Methods 16S rRNA gene sequencing and OTU table construction details. Additional details.
(DOCX)

S1 Fig Microbial relative abundances by class. (A) *Acropora millepora* (Am) and *Turbinaria reniformis* (Tr) under control (26.5°C and *p*CO_2_ of 364 μatm) and treatment (29.0°C and *p*CO_2_ of 750 μatm) conditions for each sample (1–6). (B) Average microbial relative abundances by Class.
(TIFF)

S1 Table Proportional abundance of the Operational Taxonomic Units (OTU). Results for *Acropora millepora* and *Turbinaria reniformis* under control (26.5°C and *p*CO_2_ of 364μatm) and treatment (29.0°C and *p*CO_2_ of 750μatm) conditions. Samples are from colonies numbered 1–6.
(XLSX)

S2 Table SIMPER analysis of OTUs. Average percent dissimilarity between species and between treatments within species in microbial OTU abundance. Control = 26.5°C and *p*CO_2_ of 364μatm, Treatment = 29.0°C and *p*CO_2_ of 750μatm. Am = *Acropora millepora*, Tr = *Turbinaria reniformis*, Av.Abund = average abundance, Av.Diss = average dissimilarity, Diss/SD = Dissimilarity /standard deviation, Contrib% = contribution percent, Cum.% = Cumulative percent contribution.
(DOCX)

S3 Table Results of a two-way ANOVA testing the effect of species and treatment on Shannon Diversity Index. Effects of species and treatment were fixed and fully crossed. *A posteriori* slice tests were used to test for differences between treatment and controls within species. df = degrees of freedom, SS = type III sum of squares of the main effects, F = F-statistic. Significant effects are bolded.
(DOCX)

S4 Table Kruskal-Wallis p-values for each physiological variable. Significant differences (p ≤ 0.05) are bolded. calc = calcification, chla = chlorophyll *a*, cells = endosymbiotic algal cell density, lipid = total lipids, protein = soluble animal protein concentration, carbs = carbohydrate concentration, biomass = coral ash free dry weight per area, h = host, a = endosymbiotic algae.
(DOCX)

S5 Table SIMPER analysis of coral physiology. Summary of average squared distance (Av.Sq.Dist = 29.54) in coral physiology between *Acropora millepora* and *Turbinaria reniformis* across all control and treatment coral fragments. SD = standard deviation, Av.Value = average, Contrib % = percent contribution, Cum. % = cumulative percent.
(DOCX)
