# Supplementary material for: Coral physiology and microbiome dynamics under combined warming and ocean acidification
Source: PLoS One. 2018 Jan 16;13(1):e0191156. doi: 10.1371/journal.pone.0191156 (PMC5770069; doi:10.1371/journal.pone.0191156)
Supplement: S1 Methods — Additional details. (DOCX) [file pone.0191156.s001.docx]

**Supporting Information**

**Coral physiology and microbiome dynamics under combined warming and ocean acidification**

Andréa G Grottoli, Paula Dalcin Martins, Michael J. Wilkins, Michael D. Johnston, Mark E Warner, Wei-Jun Cai, Todd F. Melman, Kenneth D. Hoadley, D. Tye Pettay, Stephen Levas, Verena Schoepf

**S1 Methods. 16S rRNA gene sequencing and OTU table construction.** Additional details.

Sequencing primers 515F (5′-GTGCCAGCMGCCGCGGTAA) and 806R (5′- GGACTACHVGGGTWTCTAAT) that cover archaea and bacteria were used as previously described [1]. PCR conditions and the number of cycles are available online at http://press.igsb.anl.gov/earthmicrobiome/protocols-and-standards/16s/. Samples were amplified in 3 replicates and pooled. The MoBio UltraClean PCR Clean-Up Kit was used for PCR cleanup following the manufacturer instructions. For library preparation, the Nextera XT DNA Library Preparation Kit was utilized.

For constructing the OTU table, Qiime was run with an in-house pipeline available online at https://github.com/TheWrightonLab/16S-analyses-with-QIIME as the commands bellow indicate. The mismatch allowed for barcodes was 1.5. The default parameters for sequence length were a minimum of 200 and maximum of 1000, as default parameters in split_libraries.py from Qiime, with default trimming and -q 19 for quality filtering. Bootstrap cut off was 0.51 as the default in assign_taxonomy.py. Singletons were filtered out by using stringent parameters: an OTU had to be counted at least 10 times and also it needed to be observed in at least 25% of the samples to be retained (see command line below).

Out of 303074 sequences, 293519 passed the chimera check, generating an OTU table free of mitochondrial, chloroplastal, and eukaryotic sequences, with 831 OTUs in total - 565 for *A. millepora* and 650 for *T. reniformes*.

**Commands used to run Qiime**

/ORG-Data/scripts/bin/Phylogeny_Protpipe/QIIME_PIPELINE.sh /ORG-Data/Feb2015_MiSeq16s/Undetermined_S0_L001_I1_001.fastq.gz /ORG-Data/Feb2015_MiSeq16s/Undetermined_S0_L001_R1_001.fastq.gz /ORG-Data/Feb2015_MiSeq16s/Undetermined_S0_L001_R2_001.fastq.gz /home2/projects/Paula/Coral_Microbiome_Collaboration_with_Andrea_Grottoli_16S_rRNA_gene_analyses/re-run_22aug2017/mapping_Andrea_descriptions.txt dalcinmartins.1@osu.edu 10 p 25

$1 = the unzipped barcode file

$2 = the unzipped forward reads file

$3 = the unzipped reverse reads file

$4 = the mapping file

$5 = email or NO_EMAIL

$6 = the minimum total observation count of an OTU for that OTU to be retained

$7 = p or s, where p = percent of samples an OTU must be observed in for that OTU to be retained, and s = the minimum number of samples an OTU must be observed in for that OTU to be retained

$8 = integer value for $7 Ex: 25 = 25%

**Commands used to filter out any sequences matching mitochondria, chloroplasts, or eukaryotes**

filter_taxa_from_otu_table.py -i otu_table_mc2_w_tax_no_pynast_failures.biom -o otu_table_mc2_w_tax_no_pynast_failures_no_mitoc_no_chloroplast.biom -n __mitochondria,__Chloroplast,Eukaryota

summarize_taxa.py -i otu_table_mc2_w_tax_no_pynast_failures_no_mitoc_no_chloroplast.biom -o ./tax_no_mitochondria_no_chloroplast_no_euk

**Qiime log**

Qiime script started

logfile location = /home2/projects/Paula/Coral_Microbiome_Collaboration_with_Andrea_Grottoli_16S_rRNA_gene_analyses/re-run_22aug2017/qiime_1503435656/qiime_script.log

Arg 1 barcode file= /ORG-Data/Feb2015_MiSeq16s/Undetermined_S0_L001_I1_001.fastq.gz

Arg 2 forward reads file = /ORG-Data/Feb2015_MiSeq16s/Undetermined_S0_L001_R1_001.fastq.gz

Arg 3 reverse reads file = /ORG-Data/Feb2015_MiSeq16s/Undetermined_S0_L001_R2_001.fastq.gz

Arg 4 mapping file = /home2/projects/Paula/Coral_Microbiome_Collaboration_with_Andrea_Grottoli_16S_rRNA_gene_analyses/re-run_22aug2017/mapping_Andrea_descriptions.txt

Arg 5 email address = dalcinmartins.1@osu.edu

Arg 6 = 10

Arg 7 = p

Arg 8 = 25

Qiime 1.9 environment was activated

/ORG-Data/Feb2015_MiSeq16s/Undetermined_S0_L001_I1_001.fastq.gz file exists

Converting mapping file to unix

Checking to make sure mapping file is correct

map_ok = No errors or warnings were found in mapping file.

Mapping file okay

Unzipping the 3 data files

barcode file has 72712964 lines

forward reads file has 72712964 = 18178241 sequences

reverse reads file has 72712964 = 18178241 sequences

Running join_paired_ends.py

join_paired_ends.py -f forward_reads.fastq -r reverse_reads.fastq -b barcode.fastq -o STEP1_OUT/

Made directory STEP1_OUT

made files:

fastqjoin.join_barcodes.fastq (61699868 lines = 15424967 sequences)

fastqjoin.join.fastq (61699868 lines = 15424967 sequences)

fastqjoin.un1.fastq (11013096 lines = 2753274 sequences)

fastqjoin.un2.fastq (11013096 lines = 2753274 sequences)

[This 16S rRNA gene sequencing run included multiple samples from different projects - this is why join_paired_ends.py generated the above mentioned number of sequences. Using a mapping file containing only samples utilized in this study, we selected sequences belonging only to these samples at the split_libraries_fastq.py level.]

Removing unzipped data files

Running split_libraries.py

split_libraries_fastq.py -i fastqjoin.join.fastq -b fastqjoin.join_barcodes.fastq --rev_comp_mapping_barcodes -o STEP2_OUT/ -m /home2/projects/Paula/Coral_Microbiome_Collaboration_with_Andrea_Grottoli_16S_rRNA_gene_analyses/re-run_22aug2017/mapping_Andrea_descriptions.txt -q 19

made directory STEP2_OUT

made seqs.fna (303074 sequences)

Looking for chimeric sequences in seqs.fna

identify_chimeric_seqs.py -i seqs.fna -m usearch61 -r /home2/Database/RDP_Gold/rdp_gold.fa -o usearch61_chimera/

made 11 files in directory usearch61_chimera

chimeras.txt (9555 lines = chimeras found)

Removing chimeras from seqs.fna

filter_fasta.py -f seqs.fna -o seqs_chimeras_filtered.fna -s usearch61_chimera/chimeras.txt -n

seqs_chimeras_filtered.fna (293519 sequences)

Running pick_open_reference_otus.py

pick_open_reference_otus.py -i /home2/projects/Paula/Coral_Microbiome_Collaboration_with_Andrea_Grottoli_16S_rRNA_gene_analyses/re-run_22aug2017/qiime_1503435656/STEP1_OUT/STEP2_OUT/seqs_chimeras_filtered.fna -r /home2/Database/Silva/rep_set/97_Silva_111_rep_set.fasta -o /home2/projects/Paula/Coral_Microbiome_Collaboration_with_Andrea_Grottoli_16S_rRNA_gene_analyses/re-run_22aug2017/qiime_1503435656/STEP1_OUT/STEP2_OUT/STEP3_OUT -f -a -O 40

made directory STEP_3_OUT

rep_set.fna (5983 sequences)

summarize_taxa.py -i otu_table_mc2_w_tax.biom -o taxonomy_summaries/ -L 2,3,4,5,6

makes directory taxonomy_summaries with 6 biom and 6 txt files of otu tables 2-6

taxonomy_summaries/otu_table_mc2_w_tax_L6.txt (850 lines)

percent_calculate_relative_abundance_output.txt file made

Qiime script has completed

Qiime was run on the mapping file /home2/projects/Paula/Coral_Microbiome_Collaboration_with_Andrea_Grottoli_16S_rRNA_gene_analyses/re-run_22aug2017/mapping_Andrea_descriptions.txt and the OTU tables are located at:

/home2/projects/Paula/Coral_Microbiome_Collaboration_with_Andrea_Grottoli_16S_rRNA_gene_analyses/re-run_22aug2017/qiime_1503435656/STEP1_OUT/STEP2_OUT/STEP3_OUT/taxonomy_summaries

Script finished and sending email to dalcinmartins.1@osu.edu

Qiime 1.9 environment was deactivated

**References:** 1. Caporaso J, Lauber C, Walters W, Berg-Lyons D, Huntley J, et al. (2012) Ultra-high-throughput microbial community analysis on the Illumina HiSeq and MiSeq platforms. The International Society for Microbial Ecology Journal 6: 1621-1624.
