# Supplementary figures and images for: Coral physiology and microbiome dynamics under combined warming and ocean acidification
Source: PLoS One. 2018 Jan 16;13(1):e0191156. doi: 10.1371/journal.pone.0191156 (PMC5770069; doi:10.1371/journal.pone.0191156)

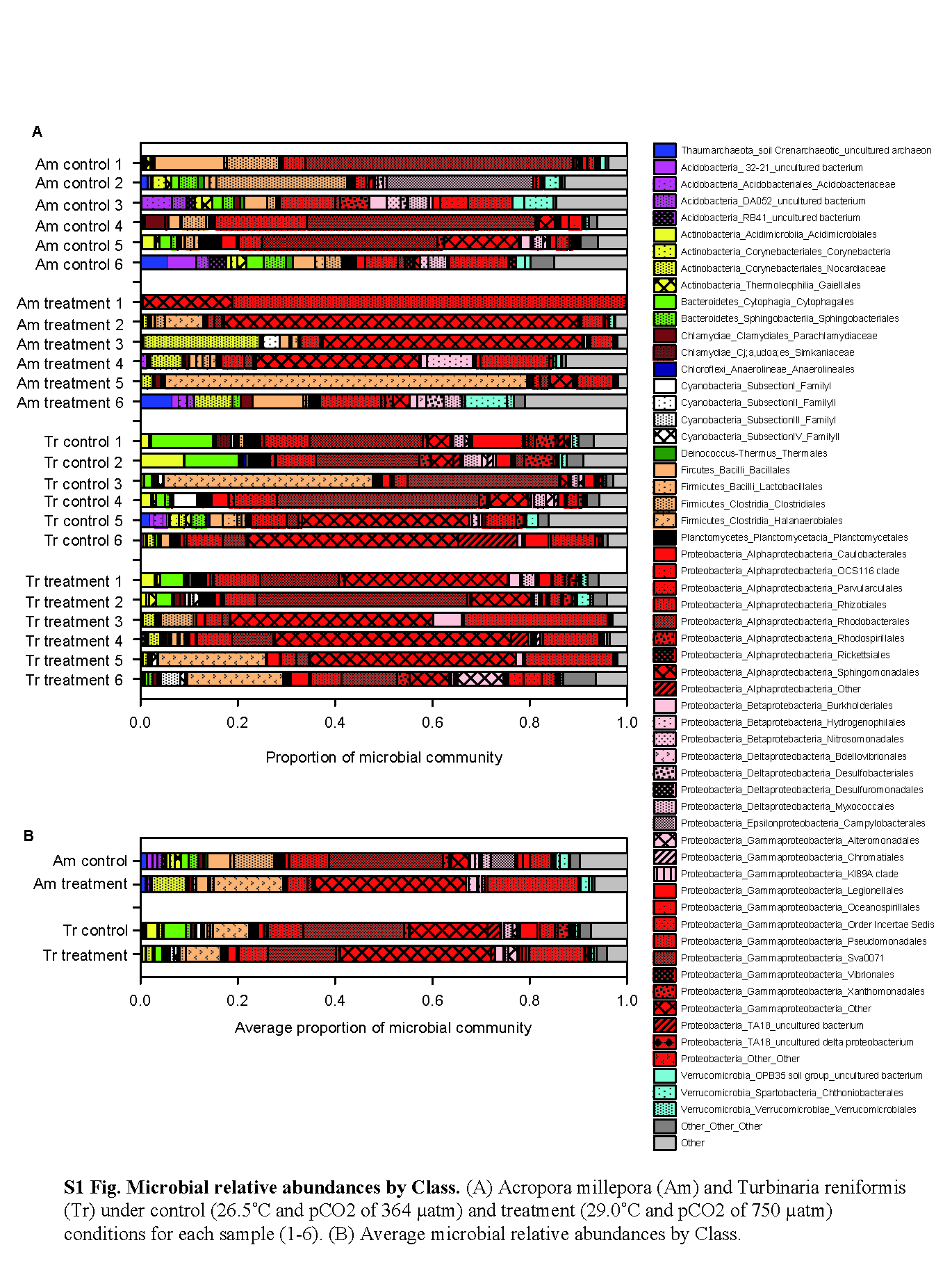

Supplement: S1 Fig — (A) Acropora millepora (Am) and Turbinaria reniformis (Tr) under control (26.5°C and pCO2 of 364 μatm) and treatment (29.0°C and pCO2 of 750 μatm) conditions for each sample (1–6). (B) Average microbial relative abundances by Class. (TIFF) [file pone.0191156.s002.tiff]
