# Supplementary material for: Coral physiology and microbiome dynamics under combined warming and ocean acidification
Source: PLoS One. 2018 Jan 16;13(1):e0191156. doi: 10.1371/journal.pone.0191156 (PMC5770069; doi:10.1371/journal.pone.0191156)
Supplement: S2 Table — Average percent dissimilarity between species and between treatments within species in microbial OTU abundance. Control = 26.5°C and pCO2 of 364μatm, Treatment = 29.0°C and pCO2 of 750μatm. Am = Acropora millepora, Tr = Turbinaria reniformis, Av.Abund = average abundance, Av.Diss = average dissimilarity, Diss/SD = Dissimilarity /standard deviation, Contrib% = contribution percent, Cum.% = Cumulative percent contribution. (DOCX) [file pone.0191156.s004.docx]

**Supporting Information**

**Coral physiology and microbiome dynamics under combined warming and ocean acidification**

Andréa G Grottoli, Paula Dalcin Martins, Michael J. Wilkins, Michael D. Johnston, Mark E Warner, Wei-Jun Cai, Todd F. Melman, Kenneth D. Hoadley, D. Tye Pettay, Stephen Levas, Verena Schoepf

**S2 Table.** **SIMPER analysis of OTUs**. Average percent dissimilarity between treatments within species, and between species, in microbial OTU abundance, with a cut off for low contributions of 90%. Control = 26.5˚C and *p*CO_2_ of 364µatm, Treatment = 29.0˚C and *p*CO_2_ of 750µatm. Am = *Acropora millepora*, Tr = *Turbinaria reniformis*, Av.Abund = average abundance, Av.Diss = average dissimilarity, Diss/SD = Dissimilarity /standard deviation, %Contrib = percent contribution, %Cum= Cumulative percent contribution.

1. *Acropora millepora* Control and Treatment (average dissimilarity 77.05)

|  |  |  |  |  |  | **Avg. Abund.** | **Avg. Abund.** | **Avg** | **Diss/** | **%** |
| --- | --- | --- | --- | --- | --- | --- | --- | --- | --- | --- |
| **Operational Taxonomic Unit** | | | | | | **Control** | **Treat** | **Diss.** | **SD** | **Contrib** |
| Bacteria | __Proteobacteria | __Alphaproteobacteria | __Sphingomonadales | __Sphingomonadaceae | __Sphingomonas | 0.14 | 0.49 | 2.98 | 1.43 | 3.87 |
| Bacteria | __Proteobacteria | __Gammaproteobacteria | __Pseudomonadales | __Pseudomonadaceae | __Pseudomonas | 0.13 | 0.33 | 2.27 | 0.75 | 2.94 |
| Bacteria | __Firmicutes | __Clostridia | __Halanaerobiales | __Halanaerobiaceae | __Halanaerobium | 0.05 | 0.24 | 1.73 | 0.71 | 2.25 |
| Bacteria | __Actinobacteria | __Corynebacteriales | __Nocardiaceae | __Rhodococcus | __Rhodococcus_fascians | 0.01 | 0.22 | 1.58 | 1.34 | 2.05 |
| Bacteria | __Proteobacteria | __Alphaproteobacteria | __Rhodobacterales | __Rhodobacteraceae | __Ruegeria | 0.18 | 0.06 | 1.44 | 0.89 | 1.87 |
| Bacteria | __Proteobacteria | __Alphaproteobacteria | __Rhodobacterales | __Rhodobacteraceae | __Pseudovibrio | 0.13 | 0 | 1.21 | 0.45 | 1.57 |
| Bacteria | __Proteobacteria | __Alphaproteobacteria | __Rhodobacterales | __Rhodobacteraceae | Other | 0.15 | 0.06 | 0.99 | 1.1 | 1.28 |
| Bacteria | __Firmicutes | __Bacilli | __Bacillales | __Staphylococcaceae | __Staphylococcus | 0.15 | 0.1 | 0.91 | 0.97 | 1.19 |
| Bacteria | __Proteobacteria | __Alphaproteobacteria | __Rhizobiales | __Methylobacteriaceae | __Methylobacterium | 0.16 | 0.11 | 0.86 | 1.11 | 1.12 |
| Bacteria | Verrucomicrobia | __Spartobacteria | __Chthoniobacterales | __DA101_soil_group | __uncultured_bacterium | 0.11 | 0.09 | 0.84 | 1.3 | 1.09 |
| Bacteria | __Proteobacteria | __Epsilonproteobacteria | __Campylobacterales | __Campylobacteraceae | __Campylobacter | 0.09 | 0 | 0.77 | 0.43 | 1 |
| Archaea | __Thaumarchaeota | __Soil_Crenarchaeotic_Group(SCG) | __uncultured_archaeon | Other | Other | 0.08 | 0.05 | 0.75 | 1.03 | 0.97 |
| Bacteria | __Firmicutes | __Clostridia | __Clostridiales | __Clostridiaceae | __Clostridium | 0.11 | 0.04 | 0.71 | 1.47 | 0.92 |
| Bacteria | __Proteobacteria | __Gammaproteobacteria | __Pseudomonadales | __Moraxellaceae | __Acinetobacter | 0.1 | 0.05 | 0.7 | 1.18 | 0.9 |
| Bacteria | __Proteobacteria | __Gammaproteobacteria | __Oceanospirillales | __Hahellaceae | __Endozoicomonas | 0.07 | 0.03 | 0.61 | 0.98 | 0.79 |
| Bacteria | __Proteobacteria | __Deltaproteobacteria | __GR-WP33-30 | __uncultured_bacterium | Other | 0.1 | 0.05 | 0.61 | 1.09 | 0.79 |
| Unassigned | Other | Other | Other | Other | Other | 0.13 | 0.07 | 0.61 | 1.16 | 0.79 |
| Bacteria | __Chlamydiae | __Chlamydiales | __Parachlamydiaceae | __Neochlamydia | Other | 0.05 | 0.05 | 0.58 | 1.12 | 0.76 |
| Bacteria | __Proteobacteria | __Alphaproteobacteria | __Sphingomonadales | Other | Other | 0.01 | 0.07 | 0.58 | 0.79 | 0.75 |
| Bacteria | __Proteobacteria | __Alphaproteobacteria | __Rhizobiales | __Xanthobacteraceae | __uncultured | 0.05 | 0.06 | 0.57 | 0.91 | 0.74 |
| Bacteria | __Firmicutes | __Clostridia | __Clostridiales | __Family_XI_Incertae_Sedis | __Peptoniphilus | 0.06 | 0.02 | 0.57 | 0.56 | 0.74 |
| Bacteria | __Acidobacteria | __32-21 | __uncultured_bacterium | Other | Other | 0.08 | 0.04 | 0.56 | 0.87 | 0.73 |
| Bacteria | __Acidobacteria | __DA052 | __uncultured_bacterium | Other | Other | 0.06 | 0.04 | 0.55 | 1 | 0.71 |
| Bacteria | __Firmicutes | __Clostridia | __Clostridiales | __Family_XI_Incertae_Sedis | __Finegoldia | 0.06 | 0.01 | 0.54 | 0.71 | 0.7 |
| Bacteria | __Firmicutes | __Clostridia | __Clostridiales | __Clostridiaceae | __Clostridiisalibacter | 0.05 | 0 | 0.52 | 0.46 | 0.68 |
| Bacteria | __Acidobacteria | __RB41 | __uncultured_bacterium | Other | Other | 0.06 | 0.01 | 0.5 | 0.84 | 0.65 |
| Bacteria | Verrucomicrobia | __OPB35_soil_group | __uncultured_bacterium | Other | Other | 0.06 | 0.02 | 0.49 | 1.05 | 0.64 |
| Bacteria | __Proteobacteria | __Gammaproteobacteria | __Legionellales | __Legionellaceae | __uncultured | 0.07 | 0.01 | 0.49 | 1.87 | 0.64 |
| Bacteria | __Bacteroidetes | __Sphingobacteriia | __Sphingobacteriales | __Chitinophagaceae | __uncultured | 0.06 | 0.01 | 0.47 | 1.07 | 0.61 |
| Bacteria | __Firmicutes | __Clostridia | __Clostridiales | __Family_XI_Incertae_Sedis | __Anaerococcus | 0.06 | 0.01 | 0.46 | 1.1 | 0.6 |
| Bacteria | __Proteobacteria | __Betaproteobacteria | __Hydrogenophilales | __Hydrogenophilaceae | __Hydrogenophilus | 0 | 0.06 | 0.46 | 0.6 | 0.6 |
| Bacteria | __Actinobacteria | __Thermoleophilia | __Gaiellales | __uncultured | __uncultured_bacterium | 0.06 | 0.01 | 0.45 | 1.15 | 0.59 |
| Bacteria | __Proteobacteria | __Betaproteobacteria | __Nitrosomonadales | __Nitrosomonadaceae | __uncultured | 0.05 | 0 | 0.43 | 1.2 | 0.56 |
| Bacteria | __Proteobacteria | __Deltaproteobacteria | __Myxococcales | __Cystobacterineae | __uncultured | 0.05 | 0.02 | 0.43 | 0.96 | 0.56 |
| Bacteria | __Firmicutes | __Bacilli | __Bacillales | __Family_XII_Incertae_Sedis | __Exiguobacterium | 0.05 | 0 | 0.42 | 0.92 | 0.54 |
| Bacteria | __Proteobacteria | __Gammaproteobacteria | __Legionellales | __Coxiellaceae | __Coxiella | 0.06 | 0.01 | 0.42 | 1.5 | 0.54 |
| Bacteria | __Proteobacteria | __Alphaproteobacteria | __Rhizobiales | __Hyphomicrobiaceae | __Filomicrobium | 0.07 | 0.04 | 0.41 | 1.25 | 0.53 |
| Bacteria | __Proteobacteria | __Alphaproteobacteria | __Rhodospirillales | __Rhodospirillaceae | __uncultured | 0.05 | 0.01 | 0.4 | 1.03 | 0.52 |
| Bacteria | __Proteobacteria | __Gammaproteobacteria | __Vibrionales | __Vibrionaceae | __Vibrio | 0.05 | 0 | 0.39 | 1.05 | 0.5 |
| Bacteria | __Proteobacteria | __Deltaproteobacteria | __Desulfovibrionales | __Desulfovibrionaceae | __Desulfovibrio | 0.01 | 0.05 | 0.38 | 0.7 | 0.49 |
| Bacteria | __Firmicutes | __Bacilli | __Bacillales | __Bacillaceae | __Bacillus | 0.03 | 0.04 | 0.37 | 0.97 | 0.48 |
| Bacteria | __Bacteroidetes | __Sphingobacteriia | __Sphingobacteriales | __Chitinophagaceae | __Ferruginibacter | 0.04 | 0 | 0.36 | 0.55 | 0.47 |
| Bacteria | __Planctomycetes | __Planctomycetacia | __Planctomycetales | __Planctomycetaceae | __Pirellula | 0.05 | 0.03 | 0.36 | 1.23 | 0.47 |
| Bacteria | __Proteobacteria | __Alphaproteobacteria | __Rhizobiales | __Phyllobacteriaceae | __Nitratireductor | 0.04 | 0.01 | 0.36 | 0.64 | 0.47 |
| Bacteria | Deinococcus-Thermus | __Deinococci | __Thermales | __Thermaceae | __Thermus | 0.04 | 0 | 0.35 | 0.9 | 0.45 |
| Bacteria | __Proteobacteria | __Deltaproteobacteria | __Myxococcales | __Nannocystineae | __Haliangiaceae | 0.03 | 0.03 | 0.35 | 0.65 | 0.45 |
| Bacteria | __Actinobacteria | __Corynebacteriales | __Corynebacteriaceae | __Corynebacterium | Other | 0.05 | 0.02 | 0.34 | 1.19 | 0.44 |
| Bacteria | __Proteobacteria | __Deltaproteobacteria | __Sh765B-TzT-29 | __uncultured_bacterium | Other | 0.04 | 0.01 | 0.33 | 0.59 | 0.43 |
| Bacteria | __Proteobacteria | __Alphaproteobacteria | __Rhodobacterales | __Rhodobacteraceae | __uncultured | 0.04 | 0.01 | 0.33 | 0.91 | 0.43 |
| Bacteria | __Acidobacteria | __DA023 | __uncultured_bacterium | Other | Other | 0.02 | 0.04 | 0.33 | 0.84 | 0.43 |
| Bacteria | __Proteobacteria | __Deltaproteobacteria | __Desulfobacterales | __Nitrospinaceae | __Candidatus_Entotheonella | 0.04 | 0 | 0.33 | 0.8 | 0.42 |
| Bacteria | __Nitrospirae | __Nitrospira | __Nitrospirales | __Nitrospiraceae | __Nitrospira | 0.02 | 0.04 | 0.32 | 1.1 | 0.41 |
| Bacteria | Verrucomicrobia | __Verrucomicrobiae | __Verrucomicrobiales | __Verrucomicrobiaceae | __Haloferula | 0.03 | 0.03 | 0.31 | 1.09 | 0.4 |
| Bacteria | __Acidobacteria | __Acidobacteriales | __Acidobacteriaceae | __uncultured | __uncultured_bacterium | 0.03 | 0.02 | 0.31 | 0.75 | 0.4 |
| Bacteria | __Planctomycetes | __Planctomycetacia | __Planctomycetales | __Planctomycetaceae | __Planctomyces | 0.04 | 0.02 | 0.31 | 1.05 | 0.4 |
| Bacteria | __Cyanobacteria | __MLE1-12 | __uncultured_bacterium | Other | Other | 0.01 | 0.04 | 0.31 | 0.67 | 0.4 |
| Bacteria | __Chlamydiae | __Chlamydiales | __Family_Incertae_Sedis | __Criblamydia | Other | 0.03 | 0.01 | 0.3 | 0.69 | 0.39 |
| Bacteria | __Proteobacteria | __Alphaproteobacteria | __Rhodospirillales | __DA111 | __uncultured_bacterium | 0.04 | 0 | 0.3 | 0.55 | 0.39 |
| Bacteria | __Proteobacteria | __Alphaproteobacteria | __Rhizobiales | __Phyllobacteriaceae | __uncultured | 0.03 | 0.01 | 0.3 | 0.75 | 0.39 |
| Bacteria | __Proteobacteria | __Betaproteobacteria | __Burkholderiales | __Comamonadaceae | Other | 0.03 | 0.01 | 0.29 | 0.72 | 0.38 |
| Bacteria | __Proteobacteria | __Gammaproteobacteria | __NKB5 | __uncultured_bacterium | Other | 0.04 | 0 | 0.29 | 0.83 | 0.37 |
| Bacteria | __Planctomycetes | __Planctomycetacia | __Planctomycetales | __Planctomycetaceae | __uncultured | 0.05 | 0.03 | 0.28 | 1.27 | 0.37 |
| Bacteria | __Proteobacteria | __Alphaproteobacteria | __Rhizobiales | __Xanthobacteraceae | Other | 0.02 | 0.03 | 0.28 | 1.03 | 0.37 |
| Bacteria | __Acidobacteria | __Acidobacteriales | __Acidobacteriaceae | __uncultured | __uncultured_Acidobacteria_bacterium | 0 | 0.04 | 0.28 | 0.94 | 0.36 |
| Bacteria | __Planctomycetes | __Planctomycetacia | __Planctomycetales | __Planctomycetaceae | __Pir4_lineage | 0.03 | 0.03 | 0.28 | 1.19 | 0.36 |
| Bacteria | Candidate_division_WS3 | __uncultured_bacterium | Other | Other | Other | 0.01 | 0.03 | 0.28 | 0.64 | 0.36 |
| Bacteria | __Acidobacteria | __Candidatus_Solibacter | __uncultured_bacterium | Other | Other | 0.01 | 0.03 | 0.28 | 1 | 0.36 |
| Bacteria | __Proteobacteria | __Alphaproteobacteria | Other | Other | Other | 0.03 | 0.03 | 0.27 | 1.22 | 0.35 |
| Bacteria | __Bacteroidetes | __Cytophagia | __Cytophagales | __Cytophagaceae | __Flexibacter | 0.03 | 0 | 0.27 | 0.43 | 0.35 |
| Bacteria | __Firmicutes | __Bacilli | __Bacillales | __Alicyclobacillaceae | __Alicyclobacillus | 0.03 | 0.01 | 0.27 | 0.73 | 0.35 |
| Bacteria | __Proteobacteria | __Alphaproteobacteria | __Rhizobiales | __Methylobacteriaceae | __Microvirga | 0.03 | 0 | 0.26 | 0.47 | 0.34 |
| Bacteria | __Proteobacteria | __Betaproteobacteria | __Burkholderiales | __Oxalobacteraceae | Other | 0.03 | 0.02 | 0.26 | 1.03 | 0.34 |
| Bacteria | __Actinobacteria | __Corynebacteriales | __Corynebacteriaceae | __Corynebacterium | __uncultured_bacterium | 0.03 | 0.01 | 0.26 | 0.56 | 0.34 |
| Bacteria | __Proteobacteria | __Gammaproteobacteria | __Pseudomonadales | __Moraxellaceae | __Enhydrobacter | 0.03 | 0 | 0.26 | 0.68 | 0.34 |
| Bacteria | __Proteobacteria | __Alphaproteobacteria | __Rhizobiales | __Bradyrhizobiaceae | __Bradyrhizobium | 0.01 | 0.03 | 0.26 | 0.98 | 0.34 |
| Bacteria | __Planctomycetes | __Planctomycetacia | __Planctomycetales | __Planctomycetaceae | __Blastopirellula | 0.04 | 0.02 | 0.26 | 1.24 | 0.33 |
| Bacteria | __Proteobacteria | __Alphaproteobacteria | __Rhizobiales | __Rhodobiaceae | __Rhodobium | 0.03 | 0.03 | 0.25 | 1.39 | 0.33 |
| Bacteria | __Proteobacteria | __Deltaproteobacteria | __Desulfobacterales | __Desulfobulbaceae | __uncultured | 0 | 0.03 | 0.25 | 0.52 | 0.33 |
| Bacteria | __Actinobacteria | __Thermoleophilia | __Gaiellales | __uncultured | __uncultured_actinobacterium | 0.03 | 0.01 | 0.25 | 1.15 | 0.33 |
| Bacteria | __Firmicutes | __Clostridia | __Clostridiales | __Veillonellaceae | Other | 0.03 | 0 | 0.25 | 0.43 | 0.33 |
| Bacteria | __Firmicutes | __Bacilli | __Lactobacillales | __Streptococcaceae | __Streptococcus | 0.04 | 0.03 | 0.25 | 1.19 | 0.33 |
| Bacteria | __Proteobacteria | __Gammaproteobacteria | __Enterobacteriales | __Enterobacteriaceae | __Enterobacter | 0.02 | 0.02 | 0.25 | 0.89 | 0.32 |
| Bacteria | __Cyanobacteria | __SubsectionII | __FamilyII | __Chroococcidiopsis | __uncultured_bacterium | 0 | 0.03 | 0.25 | 0.44 | 0.32 |
| Bacteria | __Planctomycetes | __Planctomycetacia | __Planctomycetales | __Planctomycetaceae | __Rhodopirellula | 0.03 | 0.02 | 0.25 | 1.22 | 0.32 |
| Bacteria | __Proteobacteria | __Betaproteobacteria | __Burkholderiales | __Alcaligenaceae | __Alcaligenes | 0.01 | 0.02 | 0.24 | 0.71 | 0.32 |
| Bacteria | __TM6 | __uncultured_bacterium | Other | Other | Other | 0.02 | 0.03 | 0.24 | 1.5 | 0.31 |
| Bacteria | __Proteobacteria | __TA18 | __uncultured_bacterium | Other | Other | 0.03 | 0.01 | 0.23 | 1.1 | 0.3 |
| Bacteria | __Proteobacteria | __Alphaproteobacteria | __Caulobacterales | __Caulobacteraceae | __uncultured | 0.03 | 0 | 0.23 | 0.59 | 0.3 |
| Bacteria | __Proteobacteria | __Alphaproteobacteria | __Caulobacterales | __Caulobacteraceae | __Brevundimonas | 0.03 | 0 | 0.22 | 0.53 | 0.29 |
| Bacteria | __Proteobacteria | __Deltaproteobacteria | __GR-WP33-30 | __uncultured_delta_proteobacterium | Other | 0 | 0.03 | 0.22 | 0.71 | 0.29 |
| Bacteria | Verrucomicrobia | __Verrucomicrobiae | __Verrucomicrobiales | __DEV007 | __uncultured_bacterium | 0.01 | 0.03 | 0.22 | 0.75 | 0.29 |
| Bacteria | __Firmicutes | __Bacilli | __Lactobacillales | __Carnobacteriaceae | __Granulicatella | 0.03 | 0 | 0.22 | 0.55 | 0.29 |
| Bacteria | __Firmicutes | __Bacilli | __Lactobacillales | __Lactobacillaceae | __Lactobacillus | 0.02 | 0.01 | 0.22 | 0.92 | 0.28 |
| Bacteria | __Bacteroidetes | __Sphingobacteriia | __Sphingobacteriales | __Saprospiraceae | __uncultured | 0.01 | 0.02 | 0.22 | 0.61 | 0.28 |
| Bacteria | __Proteobacteria | __Betaproteobacteria | __Nitrosomonadales | __Nitrosomonadaceae | Other | 0.03 | 0 | 0.22 | 0.43 | 0.28 |
| Bacteria | __Bacteroidetes | __Cytophagia | __Cytophagales | __Flammeovirgaceae | __uncultured | 0.02 | 0.02 | 0.22 | 0.87 | 0.28 |
| Bacteria | __Acidobacteria | __DA023 | __uncultured_Acidobacteria_bacterium | Other | Other | 0.02 | 0.01 | 0.22 | 0.63 | 0.28 |
| Bacteria | __Actinobacteria | __Frankiales | __Acidothermaceae | __Acidothermus | __uncultured_bacterium | 0.03 | 0.01 | 0.21 | 0.79 | 0.28 |
| Bacteria | __Proteobacteria | __Alphaproteobacteria | __Rhodobacterales | __Rhodobacteraceae | __Paracoccus | 0.03 | 0.01 | 0.21 | 0.76 | 0.28 |
| Bacteria | __Actinobacteria | __Acidimicrobiia | __Acidimicrobiales | __uncultured | __uncultured_bacterium | 0.03 | 0 | 0.21 | 0.66 | 0.27 |
| Bacteria | __Proteobacteria | __Alphaproteobacteria | __Rhodospirillales | __wr0007 | Other | 0 | 0.03 | 0.21 | 0.63 | 0.27 |
| Bacteria | __Bacteroidetes | __Cytophagia | __Cytophagales | __Cytophagaceae | __Hymenobacter | 0.02 | 0 | 0.2 | 0.5 | 0.27 |
| Bacteria | __Actinobacteria | __Thermoleophilia | __Gaiellales | __Gaiellaceae | __Gaiella | 0.02 | 0 | 0.2 | 0.46 | 0.26 |
| Bacteria | __Proteobacteria | __Gammaproteobacteria | Other | Other | Other | 0.03 | 0.02 | 0.2 | 1.16 | 0.26 |
| Bacteria | __Firmicutes | __Clostridia | __Clostridiales | __Family_XVIII_Incertae_Sedis | __Symbiobacterium | 0.02 | 0.01 | 0.2 | 0.78 | 0.26 |
| Bacteria | __Proteobacteria | __Alphaproteobacteria | __Rickettsiales | __Family_Incertae_Sedis | __Caedibacter | 0.02 | 0 | 0.2 | 0.43 | 0.25 |
| Bacteria | __Proteobacteria | __Deltaproteobacteria | __Myxococcales | __0319-6G20 | __uncultured_bacterium | 0.02 | 0.01 | 0.19 | 0.71 | 0.25 |
| Bacteria | __Acidobacteria | __Candidatus_Chloracidobacterium | __uncultured_bacterium | Other | Other | 0.02 | 0 | 0.19 | 0.74 | 0.25 |
| Bacteria | __Bacteroidetes | __Cytophagia | __Cytophagales | __Cytophagaceae | __Siphonobacter | 0.02 | 0 | 0.19 | 0.43 | 0.25 |
| Bacteria | __Bacteroidetes | __BSV13 | __uncultured_bacterium | Other | Other | 0.02 | 0 | 0.19 | 0.43 | 0.25 |
| Bacteria | __Proteobacteria | __Alphaproteobacteria | __Rhodobacterales | __Rhodobacteraceae | __Stappia | 0.02 | 0.01 | 0.19 | 0.86 | 0.25 |
| Bacteria | __Proteobacteria | __Alphaproteobacteria | __Sphingomonadales | __Sphingomonadaceae | Other | 0 | 0.02 | 0.19 | 1.35 | 0.25 |
| Bacteria | __Actinobacteria | __Corynebacteriales | __Nocardiaceae | __Rhodococcus | Other | 0.01 | 0.03 | 0.19 | 1.43 | 0.25 |
| Bacteria | Deinococcus-Thermus | __Deinococci | __Thermales | __Thermaceae | __Meiothermus | 0.02 | 0 | 0.19 | 0.6 | 0.25 |
| Bacteria | __Proteobacteria | __Alphaproteobacteria | __OCS116_clade | Other | Other | 0.02 | 0.02 | 0.19 | 1.42 | 0.25 |
| Bacteria | __Actinobacteria | __Acidimicrobiia | __Acidimicrobiales | __OCS155_marine_group | __uncultured_bacterium | 0.02 | 0.02 | 0.19 | 1.1 | 0.24 |
| Archaea | __Thaumarchaeota | __Soil_Crenarchaeotic_Group(SCG) | __uncultured_crenarchaeote | Other | Other | 0.02 | 0 | 0.18 | 0.45 | 0.24 |
| Bacteria | __Acidobacteria | __Acidobacteriales | __Acidobacteriaceae | __uncultured | Other | 0.02 | 0.01 | 0.18 | 0.58 | 0.24 |
| Bacteria | __Proteobacteria | __Alphaproteobacteria | __Caulobacterales | __Hyphomonadaceae | __uncultured | 0.02 | 0 | 0.18 | 0.76 | 0.23 |
| Bacteria | __Acidobacteria | __RB41 | Other | Other | Other | 0.02 | 0 | 0.18 | 0.55 | 0.23 |
| Bacteria | __Proteobacteria | __Alphaproteobacteria | __DB1-14 | Other | Other | 0.02 | 0 | 0.18 | 0.43 | 0.23 |
| Bacteria | __Proteobacteria | __Alphaproteobacteria | __Rhizobiales | __Hyphomicrobiaceae | __Pedomicrobium | 0.02 | 0 | 0.17 | 0.43 | 0.23 |
| Bacteria | __Actinobacteria | __Thermoleophilia | __Solirubrobacterales | __TM146 | __uncultured_bacterium | 0.02 | 0 | 0.17 | 0.47 | 0.23 |
| Bacteria | __Acidobacteria | __11-24 | __uncultured_bacterium | Other | Other | 0.02 | 0.01 | 0.17 | 0.63 | 0.23 |
| Bacteria | __Cyanobacteria | __SubsectionI | __FamilyI | __uncultured_bacterium | Other | 0.01 | 0.02 | 0.17 | 0.82 | 0.22 |
| Bacteria | __Bacteroidetes | __Sphingobacteriia | __Sphingobacteriales | __AKYH767 | __uncultured_bacterium | 0.02 | 0 | 0.17 | 0.43 | 0.22 |
| Bacteria | __Proteobacteria | __Alphaproteobacteria | __Rhizobiales | __Phyllobacteriaceae | __Cohaesibacter | 0.02 | 0.01 | 0.17 | 1.01 | 0.22 |
| Bacteria | __Chloroflexi | __Ktedonobacteria | __Ktedonobacterales | __HSB_OF53-F07 | __uncultured_bacterium | 0.02 | 0 | 0.17 | 0.56 | 0.22 |
| Bacteria | __Chlamydiae | __Chlamydiales | __Simkaniaceae | Other | Other | 0.02 | 0.01 | 0.17 | 0.77 | 0.22 |
| Bacteria | __Proteobacteria | __Deltaproteobacteria | __Myxococcales | __Nannocystineae | __uncultured | 0.02 | 0 | 0.17 | 0.67 | 0.22 |
| Bacteria | __Firmicutes | __Clostridia | __Clostridiales | __Veillonellaceae | __Megasphaera | 0.02 | 0 | 0.17 | 0.46 | 0.21 |
| Bacteria | __Proteobacteria | __Gammaproteobacteria | __Enterobacteriales | __Enterobacteriaceae | Other | 0.02 | 0 | 0.16 | 0.6 | 0.21 |
| Bacteria | __Actinobacteria | __Pseudonocardiales | __Pseudonocardiaceae | __Pseudonocardia | Other | 0.01 | 0.01 | 0.16 | 0.68 | 0.21 |
| Bacteria | __Proteobacteria | __Deltaproteobacteria | __Desulfurellales | __Desulfurellaceae | __uncultured | 0 | 0.02 | 0.16 | 0.44 | 0.21 |
| Bacteria | __Acidobacteria | __Acidobacteriales | __Acidobacteriaceae | Other | Other | 0.02 | 0 | 0.16 | 0.43 | 0.21 |
| Bacteria | __Firmicutes | __Clostridia | __Clostridiales | __Ruminococcaceae | __uncultured | 0.01 | 0.01 | 0.16 | 0.59 | 0.21 |
| Bacteria | __Firmicutes | __Bacilli | __Bacillales | __Staphylococcaceae | __Salinicoccus | 0.02 | 0 | 0.16 | 0.59 | 0.21 |
| Bacteria | __Firmicutes | __Bacilli | __Bacillales | __Planococcaceae | __Planococcus | 0.02 | 0 | 0.16 | 0.52 | 0.21 |
| Bacteria | Verrucomicrobia | __OPB35_soil_group | __uncultured_Verrucomicrobia_subdivision_3_bacterium | Other | Other | 0.02 | 0 | 0.16 | 0.43 | 0.21 |
| Bacteria | __Actinobacteria | __Corynebacteriales | __Mycobacteriaceae | __Mycobacterium | Other | 0.01 | 0.01 | 0.16 | 0.84 | 0.21 |
| Bacteria | __Proteobacteria | __Alphaproteobacteria | __Rickettsiales | __Holosporaceae | __Holospora | 0.02 | 0 | 0.16 | 0.64 | 0.2 |
| Bacteria | __Actinobacteria | __Thermoleophilia | __Gaiellales | __uncultured | Other | 0.02 | 0.01 | 0.16 | 0.76 | 0.2 |
| Bacteria | __Proteobacteria | __Deltaproteobacteria | __Bdellovibrionales | __Bdellovibrionaceae | __Bdellovibrio | 0 | 0.02 | 0.16 | 0.67 | 0.2 |
| Bacteria | __Planctomycetes | __OM190 | __uncultured_bacterium | Other | Other | 0.01 | 0.01 | 0.15 | 0.86 | 0.2 |
| Bacteria | __Proteobacteria | __Deltaproteobacteria | __Myxococcales | __Sorangiineae | __uncultured | 0.02 | 0 | 0.15 | 0.43 | 0.2 |
| Bacteria | __Proteobacteria | __Betaproteobacteria | __B1-7BS | __uncultured_bacterium | Other | 0.02 | 0 | 0.15 | 0.54 | 0.2 |
| Bacteria | __Acidobacteria | __DA023 | Other | Other | Other | 0.02 | 0.01 | 0.15 | 0.86 | 0.2 |
| Bacteria | __Proteobacteria | __Gammaproteobacteria | __KI89A_clade | Other | Other | 0.02 | 0 | 0.15 | 0.54 | 0.2 |
| Bacteria | __Chlamydiae | __Chlamydiales | __Simkaniaceae | __Candidatus_Rhabdochlamydia | Other | 0.01 | 0.01 | 0.15 | 0.9 | 0.2 |
| Bacteria | __Proteobacteria | __Deltaproteobacteria | __Myxococcales | __0319-6G20 | __uncultured_delta_proteobacterium | 0.02 | 0 | 0.15 | 0.43 | 0.2 |
| Bacteria | __Actinobacteria | __Streptosporangiales | __Thermomonosporaceae | __Actinoallomurus | Other | 0.02 | 0 | 0.15 | 0.54 | 0.19 |
| Bacteria | __Acidobacteria | __RB25 | __uncultured_bacterium | Other | Other | 0.02 | 0 | 0.15 | 0.47 | 0.19 |
| Bacteria | __Bacteroidetes | __Bacteroidia | __Bacteroidales | __Prevotellaceae | __Prevotella | 0.01 | 0.02 | 0.15 | 0.97 | 0.19 |
| Bacteria | __Firmicutes | __Clostridia | __Clostridiales | __Peptostreptococcaceae | __uncultured | 0.02 | 0 | 0.15 | 0.5 | 0.19 |
| Bacteria | __Cyanobacteria | __uncultured | __uncultured_bacterium | Other | Other | 0.02 | 0 | 0.15 | 0.43 | 0.19 |
| Bacteria | __Proteobacteria | __Alphaproteobacteria | __Sphingomonadales | __Sphingomonadaceae | __Sphingobium | 0.01 | 0.02 | 0.15 | 0.99 | 0.19 |
| Bacteria | __Acidobacteria | __32-21 | __uncultured_Acidobacteria_bacterium | Other | Other | 0.02 | 0 | 0.14 | 0.58 | 0.19 |
| Bacteria | __Proteobacteria | __Betaproteobacteria | __Burkholderiales | __Oxalobacteraceae | __Massilia | 0.01 | 0.02 | 0.14 | 1.13 | 0.19 |
| Bacteria | __Proteobacteria | __Gammaproteobacteria | __Enterobacteriales | __Enterobacteriaceae | __Salmonella | 0.02 | 0.01 | 0.14 | 0.99 | 0.19 |
| Bacteria | __Proteobacteria | __Deltaproteobacteria | __Bdellovibrionales | __Bdellovibrionaceae | __OM27_clade | 0 | 0.02 | 0.14 | 0.53 | 0.19 |
| Bacteria | __Proteobacteria | __Alphaproteobacteria | __Rhizobiales | __Phyllobacteriaceae | Other | 0.02 | 0.01 | 0.14 | 1.04 | 0.18 |
| Bacteria | __Proteobacteria | __Alphaproteobacteria | __Rhodobacterales | __Rhodobacteraceae | __Roseobacter | 0.02 | 0 | 0.14 | 0.58 | 0.18 |
| Bacteria | __Actinobacteria | __Micromonosporales | __Micromonosporaceae | __Actinoplanes | Other | 0.01 | 0.01 | 0.14 | 0.6 | 0.18 |
| Bacteria | __Acidobacteria | __Acidobacteriales | __Acidobacteriaceae | __Acidobacterium | __uncultured_bacterium | 0.02 | 0 | 0.14 | 0.43 | 0.18 |
| Bacteria | __Acidobacteria | __DA023 | __uncultured_soil_bacterium | Other | Other | 0 | 0.02 | 0.14 | 0.44 | 0.18 |
| Bacteria | __Acidobacteria | __Candidatus_Solibacter | Other | Other | Other | 0 | 0.02 | 0.14 | 0.44 | 0.18 |
| Bacteria | __Chlamydiae | __Chlamydiales | __Parachlamydiaceae | Other | Other | 0.01 | 0.02 | 0.14 | 1.15 | 0.18 |
| Bacteria | __Chloroflexi | __Ktedonobacteria | __Ktedonobacterales | __Ktedonobacteraceae | __uncultured | 0 | 0.02 | 0.13 | 0.44 | 0.17 |
| Bacteria | __Proteobacteria | __Gammaproteobacteria | __Legionellales | __Coxiellaceae | Other | 0.01 | 0.01 | 0.13 | 0.8 | 0.17 |
| Bacteria | __Actinobacteria | __Micrococcales | Other | Other | Other | 0.01 | 0.01 | 0.13 | 0.57 | 0.17 |
| Bacteria | __Proteobacteria | __Alphaproteobacteria | __Rhodobacterales | __Rhodobacteraceae | __Labrenzia | 0.02 | 0.01 | 0.13 | 1.12 | 0.17 |
| Bacteria | __Proteobacteria | __Alphaproteobacteria | __Rhizobiales | __Phyllobacteriaceae | __Mesorhizobium | 0.02 | 0 | 0.13 | 0.57 | 0.16 |
| Bacteria | __Firmicutes | __Bacilli | __Bacillales | __Listeriaceae | __Brochothrix | 0 | 0.02 | 0.13 | 0.44 | 0.16 |
| Bacteria | __Bacteroidetes | __Sphingobacteriia | __Sphingobacteriales | __Sphingobacteriaceae | Other | 0.01 | 0 | 0.13 | 0.46 | 0.16 |
| Bacteria | __Actinobacteria | __Acidimicrobiia | __Acidimicrobiales | __Sva0996_marine_group | Other | 0.02 | 0 | 0.13 | 0.44 | 0.16 |
| Bacteria | __Proteobacteria | __Gammaproteobacteria | __Alteromonadales | __Alteromonadaceae | __Haliea | 0.02 | 0 | 0.12 | 0.66 | 0.16 |
| Bacteria | __Firmicutes | __Clostridia | __Clostridiales | __Lachnospiraceae | __uncultured | 0.01 | 0.01 | 0.12 | 0.8 | 0.16 |
| Bacteria | __Firmicutes | __Bacilli | __Lactobacillales | __Streptococcaceae | __Lactococcus | 0.01 | 0.01 | 0.12 | 0.62 | 0.16 |
| Bacteria | __Acidobacteria | __RB41 | __uncultured_Acidobacteria_bacterium | Other | Other | 0.01 | 0 | 0.12 | 0.43 | 0.16 |
| Bacteria | __Actinobacteria | __Micrococcales | __Microbacteriaceae | Other | Other | 0.02 | 0 | 0.12 | 0.49 | 0.16 |
| Bacteria | __Chloroflexi | __Caldilineae | __Caldilineales | __Caldilineaceae | __uncultured | 0 | 0.02 | 0.12 | 0.6 | 0.16 |
| Bacteria | __Proteobacteria | __Gammaproteobacteria | __Xanthomonadales | __Sinobacteraceae | __JTB255_marine_benthic_group | 0.01 | 0.01 | 0.12 | 0.94 | 0.16 |
| Bacteria | Verrucomicrobia | __Verrucomicrobiae | __Verrucomicrobiales | __DEV007 | __uncultured_Verrucomicrobia_bacterium | 0.01 | 0.01 | 0.12 | 1.01 | 0.16 |
| Bacteria | __Proteobacteria | __Alphaproteobacteria | __Rhodobacterales | __Rhodobacteraceae | __Rhodovulum | 0.02 | 0 | 0.12 | 0.8 | 0.15 |
| Bacteria | __Proteobacteria | __Alphaproteobacteria | __Rhizobiales | __Phyllobacteriaceae | __Ahrensia | 0.01 | 0.01 | 0.12 | 0.81 | 0.15 |
| Bacteria | __Proteobacteria | __Gammaproteobacteria | __Chromatiales | __Chromatiaceae | __Marichromatium | 0 | 0.02 | 0.12 | 0.87 | 0.15 |
| Bacteria | __Firmicutes | __Bacilli | __Bacillales | __Paenibacillaceae | __Paenibacillus | 0.01 | 0 | 0.12 | 0.43 | 0.15 |
| Bacteria | __Actinobacteria | __Corynebacteriales | __Corynebacteriaceae | __Corynebacterium | __Corynebacterium_freneyi | 0.01 | 0 | 0.12 | 0.43 | 0.15 |
| Bacteria | __Bacteroidetes | __Cytophagia | __Cytophagales | __Flammeovirgaceae | __Persicobacter | 0.02 | 0 | 0.12 | 0.49 | 0.15 |
| Bacteria | __Proteobacteria | __Alphaproteobacteria | __Rhizobiales | __KF-JG30-B3 | __uncultured_bacterium | 0.01 | 0 | 0.12 | 0.48 | 0.15 |
| Bacteria | __Proteobacteria | __Betaproteobacteria | __Burkholderiales | __Burkholderiaceae | __Ralstonia | 0.01 | 0.01 | 0.12 | 1 | 0.15 |
| Bacteria | __Proteobacteria | __Gammaproteobacteria | __Oceanospirillales | __Alcanivoracaceae | __Alcanivorax | 0.01 | 0.01 | 0.12 | 0.94 | 0.15 |
| Bacteria | __Proteobacteria | __Gammaproteobacteria | __Legionellales | __Coxiellaceae | __uncultured | 0.02 | 0 | 0.12 | 0.65 | 0.15 |
| Bacteria | __Proteobacteria | __Alphaproteobacteria | __Rhizobiales | Other | Other | 0.01 | 0.01 | 0.11 | 1.25 | 0.15 |
| Bacteria | __Proteobacteria | __Gammaproteobacteria | __Xanthomonadales | __Xanthomonadaceae | __Stenotrophomonas | 0 | 0.01 | 0.11 | 0.68 | 0.14 |
| Bacteria | __Proteobacteria | __Gammaproteobacteria | __Sva0071 | __uncultured_bacterium | Other | 0.01 | 0.01 | 0.11 | 0.78 | 0.14 |
| Bacteria | __Actinobacteria | Other | Other | Other | Other | 0.01 | 0 | 0.11 | 0.64 | 0.14 |
| Bacteria | __Chlamydiae | __Chlamydiales | Other | Other | Other | 0.01 | 0.01 | 0.11 | 1.07 | 0.14 |
| Bacteria | Armatimonadetes | __Chthonomonadetes | __Chthonomonadales | __Chthonomonadaceae | __Chthonomonas | 0.01 | 0 | 0.11 | 0.47 | 0.14 |
| Bacteria | __Proteobacteria | __Betaproteobacteria | __Burkholderiales | __Comamonadaceae | __Variovorax | 0.01 | 0.01 | 0.11 | 0.89 | 0.14 |
| Bacteria | __Proteobacteria | __Deltaproteobacteria | __Myxococcales | __Sorangiineae | __Sandaracinaceae | 0.01 | 0.01 | 0.11 | 0.78 | 0.14 |
| Bacteria | __Nitrospirae | __Nitrospira | __Nitrospirales | __0319-6A21 | __uncultured_bacterium | 0.01 | 0.01 | 0.1 | 0.58 | 0.13 |
| Bacteria | Candidate_division_OD1 | __uncultured_bacterium | Other | Other | Other | 0.01 | 0.01 | 0.1 | 0.77 | 0.13 |
| Bacteria | __Proteobacteria | __Alphaproteobacteria | __Rhizobiales | __Hyphomicrobiaceae | __Hyphomicrobium | 0.01 | 0.01 | 0.1 | 0.75 | 0.13 |
| Bacteria | __Actinobacteria | __Frankiales | __Acidothermaceae | __Acidothermus | __uncultured_actinobacterium | 0.01 | 0 | 0.1 | 0.53 | 0.13 |
| Bacteria | Gemmatimonadetes | __Gemmatimonadales | __Gemmatimonadaceae | __Gemmatimonas | __uncultured_Gemmatimonadetes_bacterium | 0.01 | 0 | 0.1 | 0.51 | 0.12 |
| Bacteria | __Actinobacteria | __Propionibacteriales | __Propionibacteriaceae | __Propionibacterium | Other | 0.01 | 0 | 0.09 | 0.99 | 0.12 |
| Bacteria | __Chlamydiae | __Chlamydiales | __cvE6 | __Chlamydiales | Other | 0.01 | 0 | 0.09 | 0.58 | 0.12 |
| Bacteria | __Proteobacteria | __Betaproteobacteria | __Burkholderiales | __Comamonadaceae | __Pseudorhodoferax | 0.01 | 0 | 0.09 | 0.6 | 0.12 |
| Bacteria | __Chloroflexi | __S085 | Other | Other | Other | 0.01 | 0 | 0.09 | 0.43 | 0.12 |
| Bacteria | __Proteobacteria | __Gammaproteobacteria | __Xanthomonadales | __Xanthomonadaceae | __Dyella | 0 | 0.01 | 0.09 | 0.44 | 0.12 |
| Bacteria | __Actinobacteria | __Acidimicrobiia | __Acidimicrobiales | __TM214 | __uncultured_bacterium | 0.01 | 0 | 0.09 | 0.43 | 0.12 |
| Bacteria | __Proteobacteria | __Alphaproteobacteria | __Sphingomonadales | __Erythrobacteraceae | Other | 0.01 | 0 | 0.09 | 0.67 | 0.12 |
| Bacteria | __Proteobacteria | __Gammaproteobacteria | __Legionellales | __Coxiellaceae | __Aquicella | 0.01 | 0 | 0.09 | 0.88 | 0.12 |
| Bacteria | __Bacteroidetes | __Sphingobacteriia | __Sphingobacteriales | __Sphingobacteriaceae | __Pedobacter | 0 | 0.01 | 0.09 | 0.64 | 0.12 |
| Bacteria | __Firmicutes | __Clostridia | __Clostridiales | __Peptostreptococcaceae | __Tepidibacter | 0 | 0.01 | 0.09 | 0.69 | 0.12 |
| Bacteria | Verrucomicrobia | __Spartobacteria | __Chthoniobacterales | __DA101_soil_group | Other | 0.01 | 0.01 | 0.09 | 1.07 | 0.12 |
| Bacteria | __Actinobacteria | __Micrococcales | __Dermabacteraceae | __Brachybacterium | Other | 0.01 | 0 | 0.09 | 0.59 | 0.12 |
| Bacteria | __Acidobacteria | __Holophagae | __Sva0725 | __uncultured_bacterium | Other | 0.01 | 0.01 | 0.09 | 0.82 | 0.12 |
| Bacteria | __Bacteroidetes | __Flavobacteria | __Flavobacteriales | __Flavobacteriaceae | __Flavobacterium | 0.01 | 0.01 | 0.09 | 0.81 | 0.11 |
| Bacteria | __Proteobacteria | __Alphaproteobacteria | __Rhodobacterales | __Rhodobacteraceae | __Roseobacter_clade_CHAB-I-5_lineage | 0.01 | 0.01 | 0.09 | 0.92 | 0.11 |
| Bacteria | __Actinobacteria | __Micrococcales | __Intrasporangiaceae | __Janibacter | Other | 0.01 | 0 | 0.09 | 0.43 | 0.11 |
| Bacteria | __Proteobacteria | __Alphaproteobacteria | __Rhodobacterales | __Rhodobacteraceae | __Rhodobacter | 0.01 | 0 | 0.09 | 0.78 | 0.11 |
| Bacteria | __Proteobacteria | __Gammaproteobacteria | __Thiotrichales | __EV818SWSAP88 | __uncultured_gamma_proteobacterium | 0.01 | 0 | 0.09 | 0.5 | 0.11 |
| Bacteria | __Actinobacteria | __Micrococcales | __Dermacoccaceae | Other | Other | 0.01 | 0 | 0.09 | 0.55 | 0.11 |
| Bacteria | __Firmicutes | __Clostridia | __Clostridiales | __Clostridiaceae | __Caminicella | 0.01 | 0.01 | 0.09 | 1.16 | 0.11 |
| Bacteria | __Proteobacteria | __Betaproteobacteria | __Methylophilales | __Methylophilaceae | __OM43_clade | 0.01 | 0 | 0.08 | 0.88 | 0.11 |
| Bacteria | __Bacteroidetes | __Bacteroidia | __Bacteroidales | __Rikenellaceae | __Alistipes | 0.01 | 0 | 0.08 | 0.43 | 0.11 |
| Bacteria | __Actinobacteria | __Acidimicrobiia | __Acidimicrobiales | __Sva0996_marine_group | __uncultured_bacterium | 0.01 | 0 | 0.08 | 0.76 | 0.11 |
| Bacteria | __Proteobacteria | __Alphaproteobacteria | __Rhizobiales | __Phyllobacteriaceae | __Hoeflea | 0.01 | 0.01 | 0.08 | 0.73 | 0.11 |
| Bacteria | __Proteobacteria | __Alphaproteobacteria | __Rickettsiales | __Candidatus_Odyssella | __uncultured_bacterium | 0 | 0.01 | 0.08 | 0.44 | 0.11 |
| Bacteria | __Actinobacteria | __Corynebacteriales | __Corynebacteriaceae | __uncultured | Other | 0.01 | 0.01 | 0.08 | 0.61 | 0.11 |
| Bacteria | __Proteobacteria | __Deltaproteobacteria | __GR-WP33-30 | Other | Other | 0.01 | 0 | 0.08 | 0.52 | 0.11 |
| Bacteria | __Actinobacteria | __Micrococcales | __Cellulomonadaceae | Other | Other | 0.01 | 0 | 0.08 | 0.43 | 0.1 |
| Bacteria | __Proteobacteria | __Betaproteobacteria | __Burkholderiales | __Comamonadaceae | __Pelomonas | 0.01 | 0 | 0.08 | 0.43 | 0.1 |
| Bacteria | __Proteobacteria | __Alphaproteobacteria | __Rickettsiales | __Candidatus_Odyssella | __uncultured_alpha_proteobacterium | 0 | 0.01 | 0.08 | 0.44 | 0.1 |
| Bacteria | __Gemmatimonadetes | __Gemmatimonadales | __Gemmatimonadaceae | __uncultured | __uncultured_bacterium | 0 | 0.01 | 0.08 | 0.66 | 0.1 |
| Bacteria | __Bacteroidetes | __Cytophagia | __Cytophagales | __Flammeovirgaceae | __Flexithrix | 0.01 | 0 | 0.08 | 0.44 | 0.1 |
| Bacteria | __Proteobacteria | __Alphaproteobacteria | __Rhodospirillales | __MND8 | __uncultured_bacterium | 0.01 | 0 | 0.08 | 0.43 | 0.1 |
| Bacteria | __Proteobacteria | __Gammaproteobacteria | __Alteromonadales | __Alteromonadaceae | Other | 0.01 | 0 | 0.08 | 0.44 | 0.1 |
| Bacteria | __Proteobacteria | __Alphaproteobacteria | __Rhizobiales | __A0839 | __uncultured_bacterium | 0.01 | 0 | 0.08 | 0.51 | 0.1 |
| Bacteria | __Proteobacteria | __Gammaproteobacteria | __Enterobacteriales | __Enterobacteriaceae | __Citrobacter | 0.01 | 0 | 0.08 | 0.52 | 0.1 |
| Bacteria | __Chloroflexi | __Anaerolineae | __Anaerolineales | __Anaerolineaceae | __uncultured | 0.01 | 0.01 | 0.08 | 0.82 | 0.1 |
| Bacteria | __Verrucomicrobia | __OPB35_soil_group | __uncultured_Verrucomicrobia_bacterium | Other | Other | 0 | 0.01 | 0.08 | 0.66 | 0.1 |
| Bacteria | __Proteobacteria | __Betaproteobacteria | __Burkholderiales | __Comamonadaceae | __uncultured | 0.01 | 0.01 | 0.07 | 1.12 | 0.1 |
| Bacteria | __Proteobacteria | __Deltaproteobacteria | __Desulfuromonadales | __Geobacteraceae | __Geobacter | 0.01 | 0 | 0.07 | 0.43 | 0.1 |
| Bacteria | __Proteobacteria | __Betaproteobacteria | __Rhodocyclales | __Rhodocyclaceae | __uncultured | 0.01 | 0 | 0.07 | 0.43 | 0.1 |
| Bacteria | __Proteobacteria | __Gammaproteobacteria | __Xanthomonadales | __Sinobacteraceae | __uncultured | 0 | 0.01 | 0.07 | 0.56 | 0.1 |
| Bacteria | __Cyanobacteria | __SubsectionII | __FamilyI | __Xenococcus | Other | 0.01 | 0 | 0.07 | 0.44 | 0.09 |
| Bacteria | __Proteobacteria | __Alphaproteobacteria | __DB1-14 | __uncultured_marine_bacterium | Other | 0.01 | 0 | 0.07 | 0.44 | 0.09 |
| Bacteria | __Proteobacteria | __Alphaproteobacteria | __Rhizobiales | __Hyphomicrobiaceae | __Maritalea | 0.01 | 0 | 0.07 | 0.44 | 0.09 |
| Bacteria | __Proteobacteria | __Alphaproteobacteria | __Rhodobacterales | __Rhodobacteraceae | __Roseovarius | 0.01 | 0 | 0.07 | 0.49 | 0.09 |
| Bacteria | __Firmicutes | __Erysipelotrichi | __Erysipelotrichales | __Erysipelotrichaceae | __Turicibacter | 0 | 0.01 | 0.07 | 0.65 | 0.09 |
| Bacteria | __Proteobacteria | __Gammaproteobacteria | __Thiotrichales | __Piscirickettsiaceae | Other | 0.01 | 0 | 0.07 | 0.43 | 0.09 |
| Bacteria | __Firmicutes | __Clostridia | __Clostridiales | __Clostridiaceae | __Oxobacter | 0 | 0.01 | 0.07 | 0.57 | 0.09 |
| Bacteria | __Proteobacteria | __Alphaproteobacteria | __DB1-14 | __uncultured_alpha_proteobacterium | Other | 0.01 | 0 | 0.07 | 0.68 | 0.09 |
| Bacteria | __Firmicutes | __Clostridia | __Clostridiales | __Lachnospiraceae | Other | 0 | 0.01 | 0.07 | 0.73 | 0.09 |
| Bacteria | __Proteobacteria | __Gammaproteobacteria | __Legionellales | __Legionellaceae | __Legionella | 0 | 0.01 | 0.07 | 0.52 | 0.09 |
| Bacteria | __Acidobacteria | __Order_Incertae_Sedis | __Family_Incertae_Sedis | __Bryobacter | __uncultured_bacterium | 0 | 0.01 | 0.07 | 0.61 | 0.09 |
| Bacteria | __Proteobacteria | __Gammaproteobacteria | __Chromatiales | __Ectothiorhodospiraceae | __Acidiferrobacter | 0.01 | 0 | 0.07 | 0.43 | 0.09 |
| Bacteria | __Bacteroidetes | __Bacteroidia | __Bacteroidales | __Porphyromonadaceae | __Paludibacter | 0.01 | 0 | 0.07 | 0.49 | 0.09 |
| Bacteria | __Chlamydiae | __Chlamydiales | __Chlamydiaceae | __uncultured_Chlamydiales_bacterium | Other | 0.01 | 0 | 0.07 | 0.44 | 0.09 |
| Bacteria | __Proteobacteria | __Gammaproteobacteria | __KI89A_clade | __uncultured_bacterium | Other | 0.01 | 0 | 0.07 | 0.44 | 0.09 |
| Archaea | __Euryarchaeota | __Halobacteria | __Halobacteriales | __Halobacteriaceae | Other | 0.01 | 0 | 0.07 | 0.43 | 0.08 |
| Bacteria | __Proteobacteria | __Betaproteobacteria | __Burkholderiales | __Comamonadaceae | __Tepidimonas | 0.01 | 0 | 0.06 | 0.67 | 0.08 |
| Bacteria | __Chlamydiae | __Chlamydiales | __Parachlamydiaceae | __Candidatus_Protochlamydia | Other | 0 | 0.01 | 0.06 | 0.8 | 0.08 |
| Bacteria | __Proteobacteria | __Alphaproteobacteria | __Rhodobacterales | __Rhodobacteraceae | __Wenxinia | 0.01 | 0 | 0.06 | 0.83 | 0.08 |
| Bacteria | __Verrucomicrobia | __Spartobacteria | __Chthoniobacterales | __Xiphinematobacteraceae | __Candidatus_Xiphinematobacter | 0 | 0.01 | 0.06 | 0.6 | 0.08 |
| Bacteria | __Proteobacteria | __Betaproteobacteria | __Neisseriales | __Neisseriaceae | __uncultured | 0 | 0.01 | 0.06 | 0.57 | 0.08 |
| Bacteria | __Proteobacteria | __Alphaproteobacteria | __E6aD10 | Other | Other | 0 | 0.01 | 0.06 | 0.8 | 0.08 |
| Bacteria | __Proteobacteria | __Alphaproteobacteria | __Rhizobiales | __Rhodobiaceae | __Anderseniella | 0.01 | 0 | 0.06 | 0.6 | 0.08 |
| Bacteria | __Cyanobacteria | __SubsectionIII | __FamilyI | __Lyngbya | __uncultured_bacterium | 0 | 0.01 | 0.06 | 0.68 | 0.08 |
| Bacteria | __Planctomycetes | __Phycisphaerae | __S-70 | Other | Other | 0 | 0.01 | 0.06 | 0.44 | 0.08 |
| Bacteria | __Actinobacteria | __Frankiales | __Acidothermaceae | __Acidothermus | Other | 0.01 | 0 | 0.06 | 0.43 | 0.08 |
| Bacteria | __Proteobacteria | __Alphaproteobacteria | __Rhizobiales | __MNG7 | __uncultured_bacterium | 0.01 | 0 | 0.06 | 0.43 | 0.08 |
| Bacteria | __Proteobacteria | __Alphaproteobacteria | __MNG3 | __uncultured_bacterium | Other | 0.01 | 0 | 0.06 | 0.43 | 0.08 |
| Bacteria | __Spirochaetes | __Spirochaetales | __Leptospiraceae | __Turneriella | __uncultured_bacterium | 0.01 | 0 | 0.06 | 0.44 | 0.08 |
| Bacteria | __Cyanobacteria | __SubsectionIII | __FamilyI | __Hydrocoleum | Other | 0 | 0.01 | 0.06 | 0.44 | 0.08 |
| Bacteria | __Proteobacteria | __Betaproteobacteria | __Rhodocyclales | __Rhodocyclaceae | __Ferribacterium | 0 | 0.01 | 0.06 | 0.97 | 0.08 |
| Archaea | __Thaumarchaeota | __terrestrial_group | __uncultured_archaeon | Other | Other | 0 | 0.01 | 0.06 | 0.44 | 0.08 |
| Bacteria | __Spirochaetes | __Spirochaetales | __Spirochaetaceae | __Spirochaeta | __uncultured_bacterium | 0.01 | 0 | 0.06 | 0.61 | 0.08 |
| Bacteria | __Proteobacteria | __Alphaproteobacteria | __Sphingomonadales | __Sphingomonadaceae | __Novosphingobium | 0 | 0.01 | 0.06 | 0.68 | 0.08 |
| Bacteria | __Firmicutes | __Clostridia | __Clostridiales | __Family_XII_Incertae_Sedis | __Fusibacter | 0 | 0.01 | 0.06 | 0.58 | 0.07 |
| Bacteria | __Proteobacteria | __Alphaproteobacteria | __Rhizobiales | __Hyphomicrobiaceae | Other | 0 | 0.01 | 0.06 | 0.79 | 0.07 |
| Bacteria | __Proteobacteria | __Betaproteobacteria | __Burkholderiales | __Comamonadaceae | __Delftia | 0.01 | 0 | 0.06 | 0.68 | 0.07 |
| Bacteria | __Firmicutes | __Bacilli | __Lactobacillales | __Aerococcaceae | __Abiotrophia | 0 | 0.01 | 0.06 | 0.55 | 0.07 |
| Bacteria | __Proteobacteria | __Alphaproteobacteria | __Rhodospirillales | __Acetobacteraceae | __Roseomonas | 0 | 0.01 | 0.06 | 0.66 | 0.07 |
| Bacteria | __Proteobacteria | __Gammaproteobacteria | __EC3 | __uncultured_bacterium | Other | 0.01 | 0 | 0.06 | 0.62 | 0.07 |
| Bacteria | __Firmicutes | __Bacilli | __Lactobacillales | __Carnobacteriaceae | Other | 0 | 0.01 | 0.06 | 0.44 | 0.07 |
| Bacteria | __Actinobacteria | __Micromonosporales | __Micromonosporaceae | __Catelliglobosispora | __uncultured_bacterium | 0.01 | 0 | 0.06 | 0.43 | 0.07 |
| Bacteria | __Actinobacteria | __Thermoleophilia | __Solirubrobacterales | __288-2 | __uncultured_bacterium | 0 | 0.01 | 0.06 | 0.55 | 0.07 |
| Bacteria | __Proteobacteria | __Gammaproteobacteria | __Pasteurellales | __Pasteurellaceae | __Haemophilus | 0.01 | 0 | 0.06 | 0.62 | 0.07 |
| Bacteria | __Bacteroidetes | __Cytophagia | __Cytophagales | __Flammeovirgaceae | __Marinoscillum | 0.01 | 0 | 0.05 | 0.44 | 0.07 |
| Bacteria | __Chlorobi | __Chlorobia | __Chlorobiales | __OPB56 | __uncultured_bacterium | 0.01 | 0 | 0.05 | 0.44 | 0.07 |
| Bacteria | __Proteobacteria | __Alphaproteobacteria | __Rhodospirillales | __Candidatus_Alysiosphaera | __uncultured_alpha_proteobacterium | 0.01 | 0 | 0.05 | 0.44 | 0.07 |
| Bacteria | __Proteobacteria | __Deltaproteobacteria | __Myxococcales | __Sorangiineae | __Polyangiaceae | 0.01 | 0 | 0.05 | 0.44 | 0.07 |
| Bacteria | __Bacteroidetes | __Flavobacteria | __Flavobacteriales | __Flavobacteriaceae | __Croceitalea | 0.01 | 0 | 0.05 | 0.44 | 0.07 |

1. *Turbinaria reniformis* Control and Treatment (average dissimilarity 59.03)

|  |  |  |  |  |  | **Avg.**  **Abund.** | **Avg. Abund.** | **Avg.**  **Diss.** | **Diss/**  **SD** | **%**  **Contrib** |
| --- | --- | --- | --- | --- | --- | --- | --- | --- | --- | --- |
| **Operational Taxonomic Unit** | | | | | | **Control** | **Treat** |  |  |  |
| Bacteria | __Proteobacteria | __Alphaproteobacteria | __Sphingomonadales | __Sphingomonadaceae | __Sphingomonas | 0.34 | 0.52 | 1.6 | 1.3 | 2.71 |
| Bacteria | __Firmicutes | __Clostridia | __Halanaerobiales | __Halanaerobiaceae | __Halanaerobium | 0.14 | 0.19 | 1.32 | 0.95 | 2.24 |
| Bacteria | __Proteobacteria | __Alphaproteobacteria | __Rhodobacterales | __Rhodobacteraceae | __Ruegeria | 0.3 | 0.21 | 1.19 | 1.44 | 2.01 |
| Bacteria | __Proteobacteria | __Gammaproteobacteria | __Pseudomonadales | __Pseudomonadaceae | __Pseudomonas | 0.11 | 0.23 | 0.93 | 1.37 | 1.58 |
| Bacteria | __Proteobacteria | __Gammaproteobacteria | __Pseudomonadales | __Moraxellaceae | __Acinetobacter | 0.07 | 0.17 | 0.86 | 0.99 | 1.45 |
| Bacteria | __Bacteroidetes | __Cytophagia | __Cytophagales | __Flammeovirgaceae | __Persicobacter | 0.12 | 0.06 | 0.7 | 1.11 | 1.19 |
| Bacteria | __Proteobacteria | __Alphaproteobacteria | __Rhodobacterales | __Rhodobacteraceae | Other | 0.26 | 0.21 | 0.7 | 1.28 | 1.18 |
| Bacteria | __Proteobacteria | __Alphaproteobacteria | Other | Other | Other | 0.11 | 0.06 | 0.61 | 0.83 | 1.04 |
| Bacteria | __Proteobacteria | __Gammaproteobacteria | __Legionellales | __Coxiellaceae | __Coxiella | 0.12 | 0.04 | 0.61 | 1.28 | 1.03 |
| Bacteria | __Proteobacteria | __Gammaproteobacteria | __Legionellales | __Legionellaceae | __uncultured | 0.09 | 0.01 | 0.51 | 1.23 | 0.87 |
| Bacteria | __Proteobacteria | __Deltaproteobacteria | __Myxococcales | __Sorangiineae | __Sandaracinaceae | 0.09 | 0.05 | 0.43 | 1.46 | 0.72 |
| Unassigned | Other | Other | Other | Other | Other | 0.15 | 0.13 | 0.41 | 1.66 | 0.7 |
| Bacteria | __Proteobacteria | __Alphaproteobacteria | __Rhizobiales | __Methylobacteriaceae | __Methylobacterium | 0.12 | 0.15 | 0.41 | 1.41 | 0.69 |
| Bacteria | __Bacteroidetes | __Cytophagia | __Cytophagales | __Flammeovirgaceae | __uncultured | 0.08 | 0.03 | 0.41 | 1.39 | 0.69 |
| Bacteria | __Actinobacteria | __Corynebacteriales | __Nocardiaceae | __Rhodococcus | __Rhodococcus_fascians | 0.06 | 0.1 | 0.4 | 1.46 | 0.68 |
| Bacteria | __Proteobacteria | __Alphaproteobacteria | __Rhizobiales | __Rhodobiaceae | __Rhodobium | 0.11 | 0.07 | 0.35 | 1.44 | 0.59 |
| Bacteria | __Proteobacteria | __Gammaproteobacteria | __Xanthomonadales | __Sinobacteraceae | Other | 0.07 | 0.03 | 0.35 | 1.23 | 0.59 |
| Bacteria | __Proteobacteria | __Alphaproteobacteria | __Rhodobacterales | __Rhodobacteraceae | __uncultured | 0.08 | 0.08 | 0.33 | 1.2 | 0.56 |
| Bacteria | __Verrucomicrobia | __Spartobacteria | __Chthoniobacterales | __DA101_soil_group | __uncultured_bacterium | 0.04 | 0.05 | 0.33 | 1.07 | 0.56 |
| Bacteria | __Proteobacteria | __Alphaproteobacteria | __Rhizobiales | __Hyphomicrobiaceae | __Filomicrobium | 0.1 | 0.08 | 0.33 | 1.22 | 0.56 |
| Bacteria | __Proteobacteria | __Gammaproteobacteria | __Xanthomonadales | __Sinobacteraceae | __JTB255_marine_benthic_group | 0.08 | 0.05 | 0.33 | 1.28 | 0.55 |
| Bacteria | __Proteobacteria | __Betaproteobacteria | __Burkholderiales | __Oxalobacteraceae | __Massilia | 0.01 | 0.05 | 0.31 | 0.59 | 0.53 |
| Bacteria | __Proteobacteria | __Alphaproteobacteria | __Caulobacterales | __Hyphomonadaceae | __uncultured | 0.06 | 0.08 | 0.31 | 1.33 | 0.53 |
| Bacteria | __Actinobacteria | __Acidimicrobiia | __Acidimicrobiales | __OCS155_marine_group | __uncultured_bacterium | 0.08 | 0.04 | 0.31 | 1.01 | 0.52 |
| Bacteria | __Cyanobacteria | __SubsectionIV | __FamilyII | __Rivularia | Other | 0.04 | 0.07 | 0.3 | 1.37 | 0.5 |
| Bacteria | __Cyanobacteria | __SubsectionI | __FamilyI | __Synechococcus | __uncultured_Synechococcus_sp. | 0.05 | 0.01 | 0.3 | 0.73 | 0.5 |
| Bacteria | __Proteobacteria | __Alphaproteobacteria | __Caulobacterales | __Caulobacteraceae | __uncultured | 0.06 | 0.08 | 0.29 | 1.26 | 0.49 |
| Bacteria | __Actinobacteria | __Acidimicrobiia | __Acidimicrobiales | __Sva0996_marine_group | Other | 0.05 | 0.03 | 0.29 | 1.35 | 0.49 |
| Bacteria | __Proteobacteria | __Gammaproteobacteria | __Alteromonadales | __Alteromonadaceae | Other | 0.03 | 0.04 | 0.29 | 0.94 | 0.49 |
| Bacteria | __Proteobacteria | __Alphaproteobacteria | __Rhodospirillales | __Rhodospirillaceae | __uncultured | 0.07 | 0.04 | 0.28 | 1.41 | 0.48 |
| Bacteria | __Proteobacteria | __Gammaproteobacteria | __Legionellales | __Coxiellaceae | __Aquicella | 0.05 | 0.03 | 0.28 | 1.2 | 0.48 |
| Bacteria | __Chlamydiae | __Chlamydiales | __Simkaniaceae | __Candidatus_Rhabdochlamydia | Other | 0.05 | 0.04 | 0.28 | 1 | 0.47 |
| Bacteria | __Proteobacteria | __Alphaproteobacteria | __Rhizobiales | Other | Other | 0.03 | 0.05 | 0.27 | 0.91 | 0.46 |
| Bacteria | __Proteobacteria | __Alphaproteobacteria | __Rhodobacterales | __Rhodobacteraceae | __Pseudovibrio | 0.04 | 0.03 | 0.27 | 1.13 | 0.45 |
| Bacteria | __Proteobacteria | __Alphaproteobacteria | __Rhizobiales | __Phyllobacteriaceae | __uncultured | 0.05 | 0.05 | 0.26 | 1.52 | 0.45 |
| Bacteria | __Proteobacteria | __Gammaproteobacteria | __Sva0071 | __uncultured_bacterium | Other | 0.04 | 0.02 | 0.26 | 0.95 | 0.44 |
| Bacteria | __Proteobacteria | __Gammaproteobacteria | __Order_Incertae_Sedis | __Family_Incertae_Sedis | __Marinicella | 0.01 | 0.05 | 0.26 | 0.77 | 0.44 |
| Bacteria | __Proteobacteria | __Betaproteobacteria | __Burkholderiales | __Burkholderiaceae | __Cupriavidus | 0 | 0.04 | 0.26 | 1.09 | 0.44 |
| Bacteria | __Proteobacteria | __Alphaproteobacteria | __Rhizobiales | __Xanthobacteraceae | __uncultured | 0.04 | 0 | 0.26 | 0.99 | 0.43 |
| Bacteria | __Proteobacteria | __Gammaproteobacteria | __Chromatiales | __Granulosicoccaceae | __Granulosicoccus | 0.05 | 0.03 | 0.25 | 1.32 | 0.42 |
| Bacteria | __Proteobacteria | __Gammaproteobacteria | __Oceanospirillales | Other | Other | 0.04 | 0.05 | 0.25 | 1.19 | 0.42 |
| Bacteria | __Proteobacteria | __Alphaproteobacteria | __Rhizobiales | __Phyllobacteriaceae | Other | 0.07 | 0.03 | 0.25 | 1.39 | 0.42 |
| Bacteria | __Planctomycetes | __Planctomycetacia | __Planctomycetales | __Planctomycetaceae | __Planctomyces | 0.06 | 0.04 | 0.24 | 1.3 | 0.41 |
| Bacteria | __Firmicutes | __Bacilli | __Bacillales | __Staphylococcaceae | __Staphylococcus | 0.04 | 0.05 | 0.24 | 1.32 | 0.41 |
| Bacteria | __Proteobacteria | __Alphaproteobacteria | __Rhizobiales | __Phyllobacteriaceae | __Ahrensia | 0.05 | 0.02 | 0.23 | 1.37 | 0.39 |
| Bacteria | __Actinobacteria | __Thermoleophilia | __Gaiellales | __uncultured | __uncultured_actinobacterium | 0.04 | 0.04 | 0.23 | 1.44 | 0.38 |
| Bacteria | __Chlamydiae | __Chlamydiales | __Parachlamydiaceae | __Neochlamydia | Other | 0.02 | 0.05 | 0.22 | 1.07 | 0.38 |
| Bacteria | __Proteobacteria | __Alphaproteobacteria | __Rhizobiales | __Rhodobiaceae | __Anderseniella | 0.05 | 0.02 | 0.22 | 1.23 | 0.38 |
| Bacteria | __Firmicutes | __Clostridia | __Clostridiales | __Lachnospiraceae | __uncultured | 0.01 | 0.03 | 0.22 | 0.62 | 0.37 |
| Bacteria | __Proteobacteria | __Gammaproteobacteria | __Xanthomonadales | __Sinobacteraceae | __Nevskia | 0.04 | 0 | 0.22 | 1.08 | 0.37 |
| Bacteria | __Firmicutes | __Clostridia | __Clostridiales | __Clostridiaceae | __Clostridium | 0.04 | 0.02 | 0.22 | 1.24 | 0.37 |
| Bacteria | __Proteobacteria | __Deltaproteobacteria | __Myxococcales | __0319-6G20 | __uncultured_bacterium | 0.04 | 0.03 | 0.22 | 1.14 | 0.37 |
| Bacteria | __Planctomycetes | __Planctomycetacia | __Planctomycetales | __Planctomycetaceae | __Blastopirellula | 0.05 | 0.03 | 0.22 | 1.39 | 0.37 |
| Bacteria | __Proteobacteria | __Alphaproteobacteria | __Rhodobacterales | __Rhodobacteraceae | __Stappia | 0.05 | 0.02 | 0.22 | 1.41 | 0.36 |
| Bacteria | __Proteobacteria | __Betaproteobacteria | __Burkholderiales | __Comamonadaceae | __Variovorax | 0.03 | 0.05 | 0.22 | 1.36 | 0.36 |
| Bacteria | __Proteobacteria | __TA18 | __uncultured_bacterium | Other | Other | 0.06 | 0.04 | 0.21 | 1.3 | 0.36 |
| Bacteria | __Chloroflexi | __Anaerolineae | __Anaerolineales | __Anaerolineaceae | __uncultured | 0.04 | 0.03 | 0.21 | 1.28 | 0.35 |
| Bacteria | __Proteobacteria | __Gammaproteobacteria | Other | Other | Other | 0.05 | 0.04 | 0.21 | 1.37 | 0.35 |
| Bacteria | __Proteobacteria | __Alphaproteobacteria | __Parvularculales | __Parvularculaceae | __Parvularcula | 0.04 | 0.03 | 0.2 | 1.35 | 0.34 |
| Bacteria | __Proteobacteria | __TA18 | __uncultured_delta_proteobacterium | Other | Other | 0.03 | 0.02 | 0.2 | 0.95 | 0.34 |
| Bacteria | __Planctomycetes | __Planctomycetacia | __Planctomycetales | __Planctomycetaceae | __Rhodopirellula | 0.07 | 0.04 | 0.2 | 1.12 | 0.34 |
| Bacteria | __Verrucomicrobia | __Verrucomicrobiae | __Verrucomicrobiales | __DEV007 | __uncultured_bacterium | 0.04 | 0.03 | 0.2 | 1.44 | 0.34 |
| Bacteria | __Actinobacteria | __Corynebacteriales | __Corynebacteriaceae | __Corynebacterium | Other | 0.03 | 0.02 | 0.2 | 0.79 | 0.33 |
| Bacteria | __Proteobacteria | __Alphaproteobacteria | __OCS116_clade | Other | Other | 0.06 | 0.05 | 0.19 | 1.71 | 0.33 |
| Bacteria | __Bacteroidetes | __Flavobacteria | __Flavobacteriales | __Flavobacteriaceae | __Flavobacterium | 0.03 | 0.05 | 0.19 | 1.34 | 0.33 |
| Bacteria | __Proteobacteria | Other | Other | Other | Other | 0.03 | 0.01 | 0.19 | 1.12 | 0.33 |
| Archaea | __Thaumarchaeota | __Soil_Crenarchaeotic_Group(SCG) | __uncultured_archaeon | Other | Other | 0.03 | 0.01 | 0.19 | 0.61 | 0.33 |
| Bacteria | __Acidobacteria | __32-21 | __uncultured_bacterium | Other | Other | 0.03 | 0.03 | 0.19 | 1.36 | 0.32 |
| Bacteria | __Proteobacteria | __Alphaproteobacteria | __Sphingomonadales | __Sphingomonadaceae | Other | 0.02 | 0.04 | 0.19 | 0.79 | 0.32 |
| Bacteria | __Proteobacteria | __Alphaproteobacteria | __Rhodobacterales | __Rhodobacteraceae | __Rhodovulum | 0.04 | 0.03 | 0.18 | 1.24 | 0.31 |
| Bacteria | __Actinobacteria | __Acidimicrobiia | __Acidimicrobiales | __Sva0996_marine_group | __uncultured_bacterium | 0.03 | 0.01 | 0.18 | 1.16 | 0.31 |
| Bacteria | __Proteobacteria | __Gammaproteobacteria | __Alteromonadales | __Colwelliaceae | __Thalassomonas | 0.01 | 0.03 | 0.18 | 0.53 | 0.3 |
| Bacteria | __Firmicutes | __Clostridia | __Clostridiales | __Clostridiaceae | __Clostridiisalibacter | 0 | 0.03 | 0.18 | 0.49 | 0.3 |
| Bacteria | __Bacteroidetes | __Sphingobacteriia | __Sphingobacteriales | __Chitinophagaceae | __Sediminibacterium | 0.03 | 0 | 0.17 | 1.1 | 0.3 |
| Bacteria | __TM6 | __uncultured_bacterium | Other | Other | Other | 0.07 | 0.06 | 0.17 | 1.22 | 0.3 |
| Bacteria | __Spirochaetes | __Spirochaetales | __Leptospiraceae | __uncultured | __uncultured_bacterium | 0.03 | 0.01 | 0.17 | 1.51 | 0.29 |
| Bacteria | __Firmicutes | __Clostridia | __Clostridiales | __Ruminococcaceae | __Ruminococcus | 0.02 | 0.02 | 0.17 | 0.85 | 0.29 |
| Bacteria | __Proteobacteria | __Gammaproteobacteria | __Xanthomonadales | __Sinobacteraceae | __uncultured | 0.03 | 0.01 | 0.17 | 1.04 | 0.29 |
| Bacteria | __Proteobacteria | __Gammaproteobacteria | __Vibrionales | __Vibrionaceae | __Vibrio | 0.01 | 0.03 | 0.17 | 1.51 | 0.29 |
| Bacteria | __Chloroflexi | __Caldilineae | __Caldilineales | __Caldilineaceae | __uncultured | 0.03 | 0.01 | 0.17 | 1.07 | 0.28 |
| Bacteria | __Proteobacteria | __Alphaproteobacteria | __Rhodospirillales | __DA111 | __uncultured_bacterium | 0.03 | 0 | 0.17 | 1.01 | 0.28 |
| Bacteria | __Firmicutes | __Bacilli | __Lactobacillales | __Enterococcaceae | __Enterococcus | 0.03 | 0 | 0.17 | 0.44 | 0.28 |
| Bacteria | __Verrucomicrobia | __Verrucomicrobiae | __Verrucomicrobiales | __DEV007 | __uncultured_Verrucomicrobia_bacterium | 0.03 | 0.03 | 0.17 | 1.44 | 0.28 |
| Bacteria | __Acidobacteria | __DA052 | __uncultured_bacterium | Other | Other | 0.02 | 0.01 | 0.16 | 0.89 | 0.28 |
| Bacteria | __Proteobacteria | __Alphaproteobacteria | __Sphingomonadales | __Erythrobacteraceae | Other | 0.02 | 0.04 | 0.16 | 1.62 | 0.28 |
| Bacteria | __Bacteroidetes | __Cytophagia | __Cytophagales | __Flammeovirgaceae | __Fulvivirga | 0.02 | 0.02 | 0.16 | 1.23 | 0.28 |
| Bacteria | __Bacteroidetes | __Sphingobacteriia | __Sphingobacteriales | __Saprospiraceae | __uncultured | 0.04 | 0.05 | 0.16 | 1.29 | 0.28 |
| Bacteria | __Proteobacteria | __Alphaproteobacteria | __Caulobacterales | __Caulobacteraceae | __Phenylobacterium | 0.02 | 0.03 | 0.16 | 1.19 | 0.27 |
| Bacteria | __Proteobacteria | __Gammaproteobacteria | __Alteromonadales | __Alteromonadaceae | __Alteromonas | 0 | 0.03 | 0.16 | 0.73 | 0.27 |
| Bacteria | __Proteobacteria | __Deltaproteobacteria | __Sh765B-TzT-29 | __uncultured_bacterium | Other | 0.05 | 0.03 | 0.16 | 1.35 | 0.27 |
| Bacteria | __Proteobacteria | __Gammaproteobacteria | __Enterobacteriales | __Enterobacteriaceae | __Enterobacter | 0.02 | 0.02 | 0.16 | 1.01 | 0.27 |
| Bacteria | __Bacteroidetes | __Cytophagia | __Cytophagales | __Flammeovirgaceae | __Ekhidna | 0.02 | 0.03 | 0.16 | 1.31 | 0.27 |
| Bacteria | __Proteobacteria | __Alphaproteobacteria | __Sphingomonadales | Other | Other | 0.01 | 0.03 | 0.16 | 1.58 | 0.27 |
| Bacteria | __Acidobacteria | __Acidobacteriales | __Acidobacteriaceae | __uncultured | __uncultured_bacterium | 0.03 | 0.02 | 0.16 | 1.09 | 0.26 |
| Bacteria | __Firmicutes | __Bacilli | __Lactobacillales | __Streptococcaceae | __Streptococcus | 0.02 | 0.01 | 0.16 | 1.01 | 0.26 |
| Bacteria | __Proteobacteria | __Deltaproteobacteria | __GR-WP33-30 | __uncultured_bacterium | Other | 0.04 | 0.02 | 0.16 | 0.98 | 0.26 |
| Bacteria | __Proteobacteria | __Alphaproteobacteria | __Rhizobiales | __Phyllobacteriaceae | __Hoeflea | 0.03 | 0.01 | 0.15 | 1.42 | 0.26 |
| Bacteria | __Bacteroidetes | __Cytophagia | __Cytophagales | __Flammeovirgaceae | __Flexithrix | 0.03 | 0.02 | 0.15 | 1.39 | 0.26 |
| Bacteria | __Proteobacteria | __Gammaproteobacteria | __NKB5 | __uncultured_bacterium | Other | 0.04 | 0.03 | 0.15 | 1.27 | 0.26 |
| Bacteria | __Bacteroidetes | __Sphingobacteriia | __Sphingobacteriales | __Chitinophagaceae | __uncultured | 0.03 | 0.02 | 0.15 | 1.21 | 0.26 |
| Bacteria | __Cyanobacteria | __SubsectionIII | __FamilyI | __Spirulina | __uncultured_bacterium | 0 | 0.03 | 0.15 | 0.65 | 0.26 |
| Bacteria | __Proteobacteria | __Betaproteobacteria | __Nitrosomonadales | __Nitrosomonadaceae | __uncultured | 0.02 | 0.01 | 0.15 | 0.58 | 0.26 |
| Bacteria | __Cyanobacteria | __SubsectionI | __FamilyI | __uncultured_bacterium | Other | 0.03 | 0.01 | 0.15 | 1.31 | 0.26 |
| Bacteria | __Actinobacteria | __Thermoleophilia | __Gaiellales | __uncultured | __uncultured_bacterium | 0.03 | 0.02 | 0.15 | 0.99 | 0.25 |
| Bacteria | __Proteobacteria | __Gammaproteobacteria | __E01-9C-26_marine_group | __uncultured_gamma_proteobacterium | Other | 0.03 | 0.01 | 0.15 | 1.11 | 0.25 |
| Bacteria | __Actinobacteria | __Acidimicrobiia | __Acidimicrobiales | __Sva0996_marine_group | __uncultured_actinobacterium | 0.03 | 0.02 | 0.15 | 1.19 | 0.25 |
| Bacteria | __Bacteroidetes | __Flavobacteria | __Flavobacteriales | __Flavobacteriaceae | __Cloacibacterium | 0.02 | 0 | 0.15 | 0.5 | 0.25 |
| Bacteria | __Proteobacteria | __Alphaproteobacteria | __Rhodobacterales | __Rhodobacteraceae | __Roseobacter_clade_CHAB-I-5_lineage | 0.04 | 0.02 | 0.15 | 1.03 | 0.25 |
| Bacteria | __Proteobacteria | __Deltaproteobacteria | __Desulfovibrionales | __Desulfovibrionaceae | __Desulfovibrio | 0.02 | 0.01 | 0.14 | 1.04 | 0.25 |
| Bacteria | __Actinobacteria | __Acidimicrobiia | __Acidimicrobiales | __uncultured | __uncultured_bacterium | 0.03 | 0.01 | 0.14 | 1.22 | 0.24 |
| Bacteria | __Proteobacteria | __Alphaproteobacteria | __Rhizobiales | __Bradyrhizobiaceae | __Bradyrhizobium | 0.01 | 0.03 | 0.14 | 1.62 | 0.24 |
| Bacteria | __Acidobacteria | __Holophagae | __Acanthopleuribacterales | __Acanthopleuribacteraceae | __Acanthopleuribacter | 0 | 0.03 | 0.14 | 0.83 | 0.24 |
| Bacteria | __Proteobacteria | __Alphaproteobacteria | __Rhodobacterales | __Rhodobacteraceae | __Paracoccus | 0.02 | 0.03 | 0.14 | 0.91 | 0.24 |
| Bacteria | __Proteobacteria | __Alphaproteobacteria | __Sphingomonadales | __Sphingomonadaceae | __Sphingopyxis | 0.02 | 0.03 | 0.14 | 1.4 | 0.24 |
| Bacteria | __Proteobacteria | __Alphaproteobacteria | __E6aD10 | __uncultured_Rhizobiales_bacterium | Other | 0.02 | 0.02 | 0.14 | 1.29 | 0.24 |
| Bacteria | __Acidobacteria | __DA023 | __uncultured_bacterium | Other | Other | 0.02 | 0.01 | 0.14 | 1.02 | 0.24 |
| Bacteria | __Cyanobacteria | __SubsectionI | __FamilyI | __Synechococcus | Other | 0.02 | 0.01 | 0.14 | 1.22 | 0.23 |
| Bacteria | __Cyanobacteria | __4C0d-2 | __uncultured_bacterium | Other | Other | 0 | 0.02 | 0.14 | 0.55 | 0.23 |
| Bacteria | __Planctomycetes | __Planctomycetacia | __Planctomycetales | __Planctomycetaceae | __Pirellula | 0.04 | 0.04 | 0.14 | 1.18 | 0.23 |
| Bacteria | __Proteobacteria | __Gammaproteobacteria | __Pseudomonadales | __Moraxellaceae | __Enhydrobacter | 0.01 | 0.02 | 0.14 | 1.03 | 0.23 |
| Bacteria | __Proteobacteria | __Alphaproteobacteria | __Rhodospirillales | __Rhodospirillaceae | __Thalassospira | 0 | 0.02 | 0.14 | 0.55 | 0.23 |
| Bacteria | __Proteobacteria | __Deltaproteobacteria | __Myxococcales | __Cystobacterineae | __uncultured | 0.03 | 0.02 | 0.13 | 1.34 | 0.23 |
| Bacteria | __Actinobacteria | __Micrococcales | __Microbacteriaceae | Other | Other | 0.02 | 0.01 | 0.13 | 0.58 | 0.23 |
| Bacteria | __Proteobacteria | __Alphaproteobacteria | __Rhizobiales | __MNG7 | __uncultured_bacterium | 0.01 | 0.02 | 0.13 | 1.24 | 0.23 |
| Bacteria | __Proteobacteria | __Betaproteobacteria | __Burkholderiales | __Oxalobacteraceae | Other | 0.01 | 0.02 | 0.13 | 1.08 | 0.23 |
| Bacteria | __Bacteroidetes | __Sphingobacteriia | __Sphingobacteriales | __Saprospiraceae | __Lewinella | 0.01 | 0.02 | 0.13 | 0.96 | 0.23 |
| Bacteria | __Proteobacteria | __Deltaproteobacteria | __Myxococcales | __Nannocystineae | __Haliangiaceae | 0.02 | 0.01 | 0.13 | 1.19 | 0.23 |
| Bacteria | __Proteobacteria | __Alphaproteobacteria | __Rhizobiales | __Phyllobacteriaceae | __Nitratireductor | 0.02 | 0.01 | 0.13 | 1.75 | 0.22 |
| Bacteria | __Proteobacteria | __Alphaproteobacteria | __Rhodospirillales | __Rhodospirillaceae | __Defluviicoccus | 0.02 | 0.01 | 0.13 | 1.21 | 0.22 |
| Bacteria | __Proteobacteria | __Gammaproteobacteria | __EC3 | __uncultured_bacterium | Other | 0.02 | 0 | 0.13 | 1.52 | 0.22 |
| Bacteria | __Proteobacteria | __Gammaproteobacteria | __KI89A_clade | Other | Other | 0.02 | 0.01 | 0.13 | 1.19 | 0.22 |
| Bacteria | __Bacteroidetes | __Flavobacteria | __Flavobacteriales | __Flavobacteriaceae | Other | 0.02 | 0.02 | 0.13 | 1.13 | 0.22 |
| Bacteria | __Actinobacteria | __Micrococcales | __Micrococcaceae | __Kocuria | Other | 0.02 | 0 | 0.13 | 0.63 | 0.22 |
| Bacteria | __Acidobacteria | __RB41 | __uncultured_bacterium | Other | Other | 0.01 | 0.01 | 0.13 | 0.62 | 0.22 |
| Bacteria | __Proteobacteria | __Alphaproteobacteria | __Rhizobiales | __Phyllobacteriaceae | __Cohaesibacter | 0.02 | 0.01 | 0.13 | 1.36 | 0.22 |
| Bacteria | __Acidobacteria | __11-24 | __uncultured_bacterium | Other | Other | 0.01 | 0.01 | 0.13 | 0.61 | 0.21 |
| Bacteria | __Proteobacteria | __Gammaproteobacteria | __Alteromonadales | __Alteromonadaceae | __Microbulbifer | 0.02 | 0 | 0.13 | 1.28 | 0.21 |
| Bacteria | __Proteobacteria | __Gammaproteobacteria | __Thiotrichales | __EV818SWSAP88 | __uncultured_gamma_proteobacterium | 0.02 | 0.02 | 0.13 | 1.29 | 0.21 |
| Bacteria | __Cyanobacteria | __SubsectionIII | __FamilyI | __Lyngbya | __uncultured_bacterium | 0.01 | 0.02 | 0.12 | 0.86 | 0.21 |
| Bacteria | __Proteobacteria | __Alphaproteobacteria | __Rickettsiales | __Rickettsiaceae | __Rickettsia | 0.01 | 0.01 | 0.12 | 0.93 | 0.21 |
| Bacteria | __Proteobacteria | __Alphaproteobacteria | __Rhizobiales | __Rhodobiaceae | Other | 0.02 | 0.01 | 0.12 | 1.25 | 0.21 |
| Bacteria | __Planctomycetes | __Phycisphaerae | __Phycisphaerales | __Phycisphaeraceae | __SM1A02 | 0.02 | 0.01 | 0.12 | 0.87 | 0.21 |
| Bacteria | __Proteobacteria | __Gammaproteobacteria | __Alteromonadales | __Alteromonadaceae | __Haliea | 0.03 | 0.03 | 0.12 | 1.4 | 0.21 |
| Bacteria | __Bacteroidetes | __Bacteroidia | __Bacteroidales | __Porphyromonadaceae | __Paludibacter | 0.02 | 0 | 0.12 | 0.44 | 0.2 |
| Bacteria | __Planctomycetes | __Planctomycetacia | __Planctomycetales | __Planctomycetaceae | __uncultured | 0.02 | 0.01 | 0.12 | 1.08 | 0.2 |
| Bacteria | __Actinobacteria | __Acidimicrobiia | __Acidimicrobiales | Other | Other | 0.02 | 0.02 | 0.12 | 1.27 | 0.2 |
| Bacteria | __Proteobacteria | __Alphaproteobacteria | __DB1-14 | __uncultured_marine_bacterium | Other | 0.03 | 0.02 | 0.12 | 1.1 | 0.2 |
| Bacteria | __Planctomycetes | __Planctomycetacia | __Planctomycetales | __Planctomycetaceae | __Pir4_lineage | 0.07 | 0.06 | 0.12 | 1.35 | 0.2 |
| Bacteria | __Proteobacteria | __Gammaproteobacteria | __Alteromonadales | __Alteromonadaceae | __Melitea | 0.02 | 0 | 0.12 | 0.89 | 0.2 |
| Bacteria | __Chlamydiae | __Chlamydiales | Other | Other | Other | 0.02 | 0.01 | 0.12 | 1.15 | 0.2 |
| Bacteria | __Cyanobacteria | __SubsectionIII | __FamilyI | __Limnothrix | Other | 0 | 0.02 | 0.12 | 0.44 | 0.2 |
| Bacteria | __Proteobacteria | __Deltaproteobacteria | __Bdellovibrionales | __Bdellovibrionaceae | __Bdellovibrio | 0.02 | 0.02 | 0.12 | 1.16 | 0.2 |
| Bacteria | __Planctomycetes | __OM190 | __uncultured_bacterium | Other | Other | 0.02 | 0.02 | 0.12 | 1.11 | 0.2 |
| Bacteria | __Proteobacteria | __Deltaproteobacteria | __Syntrophobacterales | __Syntrophaceae | __uncultured | 0.01 | 0.02 | 0.12 | 1.13 | 0.2 |
| Bacteria | __Bacteroidetes | __Sphingobacteriia | __Sphingobacteriales | __Chitinophagaceae | __Hydrotalea | 0.02 | 0 | 0.12 | 1.05 | 0.2 |
| Bacteria | __Chlamydiae | __Chlamydiales | __cvE6 | __Chlamydiales | Other | 0.02 | 0.01 | 0.12 | 1.25 | 0.2 |
| Bacteria | __Nitrospirae | __Nitrospira | __Nitrospirales | __0319-6A21 | __uncultured_bacterium | 0.02 | 0 | 0.11 | 0.81 | 0.19 |
| Bacteria | __Nitrospirae | __Nitrospira | __Nitrospirales | __Nitrospiraceae | __Nitrospira | 0.02 | 0.01 | 0.11 | 1.16 | 0.19 |
| Bacteria | __Proteobacteria | __Deltaproteobacteria | __GR-WP33-30 | Other | Other | 0.02 | 0.01 | 0.11 | 1.05 | 0.19 |
| Bacteria | __Bacteroidetes | __Sphingobacteriia | __Sphingobacteriales | __Chitinophagaceae | __Chitinophaga | 0.02 | 0 | 0.11 | 0.44 | 0.19 |
| Bacteria | __Acidobacteria | __BPC015 | Other | Other | Other | 0.02 | 0 | 0.11 | 0.73 | 0.19 |
| Bacteria | __Bacteroidetes | __Cytophagia | __Order_II_Incertae_Sedis | __Rhodothermaceae | __Rubricoccus | 0.01 | 0.02 | 0.11 | 1.23 | 0.19 |
| Bacteria | __Actinobacteria | __Corynebacteriales | __Nocardiaceae | __Rhodococcus | Other | 0.03 | 0.01 | 0.11 | 1.06 | 0.19 |
| Bacteria | __Bacteroidetes | __Flavobacteria | __Flavobacteriales | __Flavobacteriaceae | __Muricola | 0.02 | 0.01 | 0.11 | 1.03 | 0.19 |
| Bacteria | __Cyanobacteria | __SubsectionII | __FamilyII | __Pleurocapsa | __uncultured_bacterium | 0.01 | 0.01 | 0.11 | 0.99 | 0.19 |
| Bacteria | __Cyanobacteria | __SubsectionII | __FamilyII | __Pleurocapsa | Other | 0.02 | 0.01 | 0.11 | 1.25 | 0.19 |
| Bacteria | __Proteobacteria | __Alphaproteobacteria | __DB1-14 | __uncultured_bacterium | Other | 0.01 | 0.02 | 0.11 | 1.29 | 0.19 |
| Bacteria | __Cyanobacteria | __SubsectionIII | __FamilyI | __Leptolyngbya | Other | 0.01 | 0.02 | 0.11 | 1.15 | 0.18 |
| Bacteria | __Firmicutes | __Clostridia | __Clostridiales | __Peptostreptococcaceae | __uncultured | 0 | 0.01 | 0.11 | 0.53 | 0.18 |
| Bacteria | __Planctomycetes | __Phycisphaerae | __mle1-8 | __uncultured_bacterium | Other | 0.01 | 0.02 | 0.11 | 0.92 | 0.18 |
| Bacteria | __Firmicutes | __Bacilli | __Bacillales | __Bacillaceae | Other | 0.02 | 0 | 0.11 | 0.44 | 0.18 |
| Bacteria | __Proteobacteria | __Gammaproteobacteria | __KI89A_clade | __uncultured_bacterium | Other | 0.01 | 0.01 | 0.11 | 1.03 | 0.18 |
| Bacteria | __Verrucomicrobia | __Candidatus_Methylacidiphilum | __uncultured_Verrucomicrobia_bacterium | Other | Other | 0.02 | 0 | 0.11 | 0.59 | 0.18 |
| Bacteria | __Proteobacteria | __Alphaproteobacteria | __Sphingomonadales | __Erythrobacteraceae | __Erythrobacter | 0.02 | 0.01 | 0.11 | 1.19 | 0.18 |
| Bacteria | __Actinobacteria | __Bifidobacteriales | __Bifidobacteriaceae | __Bifidobacterium | Other | 0.02 | 0 | 0.11 | 0.58 | 0.18 |
| Bacteria | __Proteobacteria | __Gammaproteobacteria | __Oceanospirillales | __Oleiphilaceae | __Oleiphilus | 0.01 | 0.01 | 0.11 | 0.97 | 0.18 |
| Bacteria | __Spirochaetes | __Spirochaetales | __Leptospiraceae | __uncultured | Other | 0.01 | 0.02 | 0.11 | 1.05 | 0.18 |
| Bacteria | __Proteobacteria | __Gammaproteobacteria | __Alteromonadales | __Pseudoalteromonadaceae | __Pseudoalteromonas | 0.01 | 0.01 | 0.11 | 0.81 | 0.18 |
| Bacteria | __Chlamydiae | __Chlamydiales | __Simkaniaceae | Other | Other | 0.01 | 0.02 | 0.11 | 0.81 | 0.18 |
| Bacteria | __Cyanobacteria | __SubsectionII | __FamilyI | __Xenococcus | Other | 0.01 | 0.02 | 0.1 | 1.26 | 0.18 |
| Bacteria | __Proteobacteria | __Alphaproteobacteria | __Rhodobacterales | __Rhodobacteraceae | __Wenxinia | 0.02 | 0.01 | 0.1 | 1.06 | 0.18 |
| Bacteria | __Proteobacteria | __Alphaproteobacteria | __Rhizobiales | __Hyphomicrobiaceae | Other | 0.02 | 0.02 | 0.1 | 1.51 | 0.17 |
| Bacteria | __Chlamydiae | __Chlamydiales | __Parachlamydiaceae | Other | Other | 0.02 | 0.01 | 0.1 | 1.08 | 0.17 |
| Bacteria | __Proteobacteria | __Betaproteobacteria | __Burkholderiales | __Comamonadaceae | __Delftia | 0.01 | 0.01 | 0.1 | 1.17 | 0.17 |
| Bacteria | __Firmicutes | __Bacilli | __Bacillales | __Planococcaceae | __Planococcus | 0.01 | 0 | 0.1 | 0.44 | 0.17 |
| Bacteria | __Proteobacteria | __Alphaproteobacteria | __Kordiimonadales | __Kordiimonadaceae | __Kordiimonas | 0 | 0.02 | 0.1 | 0.48 | 0.17 |
| Bacteria | __Actinobacteria | __Corynebacteriales | __Mycobacteriaceae | __Mycobacterium | Other | 0.02 | 0.01 | 0.1 | 1.02 | 0.17 |
| Bacteria | __Actinobacteria | __Acidimicrobiia | __Acidimicrobiales | __Acidimicrobiaceae | __uncultured | 0.02 | 0.01 | 0.1 | 1.14 | 0.17 |
| Bacteria | __Bacteroidetes | __Flavobacteria | __Flavobacteriales | __Flavobacteriaceae | __Muricauda | 0.02 | 0.01 | 0.1 | 1.15 | 0.17 |
| Bacteria | __Proteobacteria | __Gammaproteobacteria | __Thiotrichales | __H2-104-2 | __uncultured_deep-sea_bacterium | 0.01 | 0.01 | 0.1 | 0.83 | 0.17 |
| Bacteria | __Actinobacteria | __Corynebacteriales | __Corynebacteriaceae | __Corynebacterium | __uncultured_bacterium | 0.01 | 0 | 0.1 | 0.72 | 0.17 |
| Bacteria | __Proteobacteria | __Alphaproteobacteria | __Rhodospirillales | __Rhodospirillaceae | __Pelagibius | 0.01 | 0.01 | 0.1 | 0.89 | 0.17 |
| Bacteria | __Firmicutes | __Bacilli | __Bacillales | __Family_XII_Incertae_Sedis | __Exiguobacterium | 0.01 | 0.01 | 0.1 | 0.61 | 0.17 |
| Bacteria | __Chlamydiae | __Chlamydiales | __Parachlamydiaceae | __Candidatus_Protochlamydia | Other | 0.02 | 0 | 0.1 | 1.35 | 0.17 |
| Bacteria | __Proteobacteria | __Gammaproteobacteria | __Thiotrichales | __EV818SWSAP88 | Other | 0.02 | 0 | 0.1 | 1.19 | 0.16 |
| Bacteria | __Proteobacteria | __Alphaproteobacteria | __Rhizobiales | __Phyllobacteriaceae | __Phyllobacterium | 0.02 | 0.01 | 0.1 | 1.39 | 0.16 |
| Bacteria | __Proteobacteria | __Gammaproteobacteria | __Alteromonadales | __Alteromonadaceae | __BD1-7_clade | 0.01 | 0.02 | 0.1 | 1.14 | 0.16 |
| Bacteria | __Firmicutes | __Clostridia | __Clostridiales | __Family_XI_Incertae_Sedis | __Anaerococcus | 0.01 | 0 | 0.09 | 0.65 | 0.16 |
| Bacteria | __Proteobacteria | __Alphaproteobacteria | __Rhodobacterales | __Rhodobacteraceae | __Labrenzia | 0.01 | 0.01 | 0.09 | 1.15 | 0.16 |
| Bacteria | __Actinobacteria | __Acidimicrobiia | __Acidimicrobiales | __uncultured | Other | 0.01 | 0.01 | 0.09 | 1.09 | 0.16 |
| Bacteria | __Proteobacteria | __Gammaproteobacteria | __Oceanospirillales | __Alcanivoracaceae | __Alcanivorax | 0.01 | 0.01 | 0.09 | 0.91 | 0.16 |
| Bacteria | __Actinobacteria | __Corynebacteriales | __Corynebacteriaceae | __uncultured | Other | 0 | 0.02 | 0.09 | 0.97 | 0.16 |
| Bacteria | __Proteobacteria | __Alphaproteobacteria | __Sphingomonadales | __Sphingomonadaceae | __Sphingobium | 0 | 0.01 | 0.09 | 0.93 | 0.16 |
| Bacteria | __Proteobacteria | __Gammaproteobacteria | __Legionellales | __Coxiellaceae | Other | 0.02 | 0 | 0.09 | 1.33 | 0.16 |
| Bacteria | __Proteobacteria | __Deltaproteobacteria | __Sh765B-TzT-29 | Other | Other | 0.01 | 0.01 | 0.09 | 0.93 | 0.16 |
| Bacteria | __Proteobacteria | __Betaproteobacteria | __Burkholderiales | __Comamonadaceae | __uncultured | 0.01 | 0.01 | 0.09 | 0.88 | 0.16 |
| Bacteria | __Acidobacteria | __Candidatus_Chloracidobacterium | __uncultured_bacterium | Other | Other | 0.02 | 0.01 | 0.09 | 0.99 | 0.16 |
| Bacteria | __Proteobacteria | __Gammaproteobacteria | __Alteromonadales | __Alteromonadaceae | __BD2-7 | 0.01 | 0 | 0.09 | 0.44 | 0.15 |
| Bacteria | __Firmicutes | __Bacilli | __Bacillales | __Alicyclobacillaceae | __Tumebacillus | 0.01 | 0.01 | 0.09 | 1.14 | 0.15 |
| Bacteria | __Proteobacteria | __Deltaproteobacteria | __Desulfobacterales | __Nitrospinaceae | __Candidatus_Entotheonella | 0.01 | 0.01 | 0.09 | 0.95 | 0.15 |
| Bacteria | __Proteobacteria | __Gammaproteobacteria | __Alteromonadales | __Alteromonadaceae | __OM60(NOR5)_clade | 0.01 | 0.01 | 0.09 | 1.05 | 0.15 |
| Bacteria | __Actinobacteria | __Frankiales | __Acidothermaceae | __Acidothermus | __uncultured_bacterium | 0.01 | 0 | 0.09 | 0.44 | 0.15 |
| Bacteria | __Proteobacteria | __Gammaproteobacteria | __Pseudomonadales | __Moraxellaceae | __Psychrobacter | 0.01 | 0 | 0.09 | 0.44 | 0.15 |
| Bacteria | __Actinobacteria | __Acidimicrobiia | __Acidimicrobiales | __uncultured | __uncultured_actinobacterium | 0.02 | 0 | 0.09 | 1.18 | 0.15 |
| Bacteria | __Proteobacteria | __Gammaproteobacteria | __Alteromonadales | __Alteromonadaceae | __Marinobacter | 0.01 | 0 | 0.09 | 0.93 | 0.15 |
| Bacteria | __Proteobacteria | __Deltaproteobacteria | __Bdellovibrionales | __Bacteriovoracaceae | __Peredibacter | 0.01 | 0.01 | 0.09 | 0.9 | 0.15 |
| Bacteria | __BD1-5 | __uncultured_bacterium | Other | Other | Other | 0.01 | 0.01 | 0.09 | 1.14 | 0.15 |
| Bacteria | __Acidobacteria | __Holophagae | __Sva0725 | Other | Other | 0.02 | 0 | 0.09 | 0.82 | 0.15 |
| Bacteria | __Proteobacteria | __Alphaproteobacteria | __Rhizobiales | __Family_Incertae_Sedis | __Bauldia | 0.01 | 0.02 | 0.09 | 1.18 | 0.15 |
| Bacteria | __Acidobacteria | __RB25 | __uncultured_bacterium | Other | Other | 0.01 | 0.01 | 0.09 | 0.99 | 0.15 |
| Bacteria | __Acidobacteria | __Acidobacteriales | __Acidobacteriaceae | Other | Other | 0.01 | 0 | 0.09 | 0.44 | 0.15 |
| Bacteria | __Verrucomicrobia | __Verrucomicrobiae | __Verrucomicrobiales | __Verrucomicrobiaceae | __Persicirhabdus | 0.01 | 0.01 | 0.09 | 0.82 | 0.15 |
| Bacteria | __Verrucomicrobia | __OPB35_soil_group | __uncultured_bacterium | Other | Other | 0.01 | 0.01 | 0.09 | 0.65 | 0.15 |
| Bacteria | __Bacteroidetes | __Flavobacteria | __Flavobacteriales | __Flavobacteriaceae | __Tenacibaculum | 0 | 0.01 | 0.09 | 0.85 | 0.14 |
| Bacteria | __Proteobacteria | __Alphaproteobacteria | __Rhodobacterales | __Rhodobacteraceae | __Roseovarius | 0.02 | 0.01 | 0.08 | 1.17 | 0.14 |
| Bacteria | __Proteobacteria | __Gammaproteobacteria | __Thiotrichales | __H2-104-2 | __uncultured_bacterium | 0.01 | 0 | 0.08 | 1.21 | 0.14 |
| Bacteria | __Verrucomicrobia | __Candidatus_Methylacidiphilum | __uncultured_bacterium | Other | Other | 0.01 | 0.02 | 0.08 | 1.21 | 0.14 |
| Bacteria | __Verrucomicrobia | __Verrucomicrobiae | __Verrucomicrobiales | __Verrucomicrobiaceae | __Haloferula | 0.01 | 0.01 | 0.08 | 0.95 | 0.14 |
| Bacteria | __Proteobacteria | __Alphaproteobacteria | __Rhizobiales | __Hyphomicrobiaceae | __Hyphomicrobium | 0.01 | 0.01 | 0.08 | 1.16 | 0.14 |
| Bacteria | __Proteobacteria | __Betaproteobacteria | __Burkholderiales | __Comamonadaceae | Other | 0.01 | 0.01 | 0.08 | 1.01 | 0.14 |
| Bacteria | __Verrucomicrobia | __Verrucomicrobiae | __Verrucomicrobiales | __Rubritaleaceae | __Rubritalea | 0.01 | 0.01 | 0.08 | 0.99 | 0.14 |
| Bacteria | __Cyanobacteria | __SubsectionIII | __FamilyI | __uncultured | __uncultured_bacterium | 0.01 | 0.01 | 0.08 | 0.88 | 0.14 |
| Bacteria | __Acidobacteria | __Acidobacteriales | __Acidobacteriaceae | __Candidatus_Koribacter | __uncultured_bacterium | 0.01 | 0 | 0.08 | 0.44 | 0.14 |
| Bacteria | __Chloroflexi | __TK10 | Other | Other | Other | 0.01 | 0 | 0.08 | 0.44 | 0.14 |
| Bacteria | __Cyanobacteria | __SubsectionIII | __FamilyI | Other | Other | 0.01 | 0.01 | 0.08 | 0.97 | 0.14 |
| Bacteria | __Proteobacteria | __Alphaproteobacteria | __OCS116_clade | __uncultured_bacterium | Other | 0.01 | 0.01 | 0.08 | 1.06 | 0.14 |
| Bacteria | __Proteobacteria | __Alphaproteobacteria | __Rhodospirillales | __Candidatus_Alysiosphaera | __uncultured_alpha_proteobacterium | 0 | 0.02 | 0.08 | 1.56 | 0.14 |
| Bacteria | __Acidobacteria | __DA023 | __uncultured_Acidobacteria_bacterium | Other | Other | 0.01 | 0 | 0.08 | 0.65 | 0.14 |
| Bacteria | __Gemmatimonadetes | __BD2-11_terrestrial_group | __uncultured_bacterium | Other | Other | 0.01 | 0 | 0.08 | 0.79 | 0.13 |
| Bacteria | __Planctomycetes | __Phycisphaerae | __Phycisphaerales | __Phycisphaeraceae | __Phycisphaera | 0.01 | 0.01 | 0.08 | 1.32 | 0.13 |
| Bacteria | __Proteobacteria | __Deltaproteobacteria | __Bdellovibrionales | __Bacteriovoracaceae | Other | 0.01 | 0.01 | 0.08 | 0.95 | 0.13 |
| Bacteria | __Proteobacteria | __Deltaproteobacteria | __Desulfobacterales | __Desulfobulbaceae | __uncultured | 0.01 | 0.02 | 0.08 | 1.35 | 0.13 |
| Bacteria | __Bacteroidetes | Other | Other | Other | Other | 0.01 | 0 | 0.08 | 0.88 | 0.13 |
| Bacteria | __Proteobacteria | __Alphaproteobacteria | __Rhodospirillales | __Rhodospirillaceae | Other | 0.01 | 0.01 | 0.08 | 0.99 | 0.13 |
| Bacteria | __Proteobacteria | __Gammaproteobacteria | __Oceanospirillales | __Oceanospirillaceae | __Marinobacterium | 0.01 | 0.01 | 0.08 | 0.83 | 0.13 |
| Bacteria | __Proteobacteria | __Alphaproteobacteria | __Caulobacterales | __Caulobacteraceae | __Brevundimonas | 0.01 | 0.01 | 0.08 | 1.11 | 0.13 |
| Bacteria | __Proteobacteria | __Gammaproteobacteria | __Legionellales | __Legionellaceae | __Legionella | 0.01 | 0.01 | 0.08 | 1.04 | 0.13 |
| Bacteria | __Proteobacteria | __Alphaproteobacteria | __Rhodobacterales | __Rhodobacteraceae | __Rubribacterium | 0.01 | 0 | 0.07 | 0.63 | 0.13 |
| Bacteria | __Proteobacteria | __Deltaproteobacteria | __Myxococcales | __Nannocystineae | __uncultured | 0.01 | 0.01 | 0.07 | 1.05 | 0.13 |
| Bacteria | __Bacteroidetes | __SB-1 | __uncultured_bacterium | Other | Other | 0.01 | 0.01 | 0.07 | 0.91 | 0.13 |
| Bacteria | __Verrucomicrobia | __Verrucomicrobiae | __Verrucomicrobiales | __P._palm_C_85 | Other | 0 | 0.01 | 0.07 | 0.54 | 0.12 |
| Bacteria | __Proteobacteria | __Alphaproteobacteria | __Rhodobacterales | __Rhodobacteraceae | __Rhodobacter | 0.01 | 0.01 | 0.07 | 1.22 | 0.12 |
| Bacteria | __Proteobacteria | __Deltaproteobacteria | __Desulfobacterales | __Desulfobulbaceae | __SEEP-SRB4 | 0.01 | 0 | 0.07 | 0.73 | 0.12 |
| Bacteria | __Proteobacteria | __Alphaproteobacteria | __Rhodobacterales | __Rhodobacteraceae | __Roseivivax | 0.01 | 0.01 | 0.07 | 1.01 | 0.12 |
| Bacteria | __Proteobacteria | __Gammaproteobacteria | __Legionellales | __Coxiellaceae | __uncultured | 0.01 | 0.01 | 0.07 | 1.12 | 0.12 |
| Bacteria | __Proteobacteria | __Deltaproteobacteria | __GR-WP33-30 | __uncultured_delta_proteobacterium | Other | 0.01 | 0.01 | 0.07 | 0.86 | 0.12 |
| Bacteria | __Cyanobacteria | __SubsectionI | __FamilyI | __uncultured_cyanobacterium | Other | 0 | 0.01 | 0.07 | 1.02 | 0.12 |
| Bacteria | __Bacteroidetes | __Flavobacteria | __Flavobacteriales | __Flavobacteriaceae | __Croceitalea | 0.01 | 0.01 | 0.07 | 1.15 | 0.12 |
| Bacteria | __Proteobacteria | __Gammaproteobacteria | __E01-9C-26_marine_group | Other | Other | 0.01 | 0.01 | 0.07 | 0.9 | 0.12 |
| Bacteria | __Proteobacteria | __Deltaproteobacteria | __Myxococcales | Other | Other | 0.01 | 0 | 0.07 | 1.03 | 0.12 |
| Bacteria | __Proteobacteria | __Alphaproteobacteria | __Rhizobiales | __Hyphomicrobiaceae | __Maritalea | 0 | 0.01 | 0.07 | 1.03 | 0.12 |
| Bacteria | __Bacteroidetes | __Bacteroidia | __Bacteroidales | __Porphyromonadaceae | __Proteiniphilum | 0.01 | 0 | 0.07 | 0.44 | 0.12 |
| Bacteria | __Proteobacteria | __Alphaproteobacteria | __Sphingomonadales | __Erythrobacteraceae | __uncultured | 0 | 0.01 | 0.07 | 0.5 | 0.12 |
| Bacteria | __Proteobacteria | __Betaproteobacteria | __Burkholderiales | __Burkholderiaceae | __Limnobacter | 0.01 | 0 | 0.07 | 0.96 | 0.12 |
| Bacteria | __Proteobacteria | __Gammaproteobacteria | __Chromatiales | __Chromatiaceae | __Nitrosococcus | 0 | 0.01 | 0.07 | 0.63 | 0.12 |
| Bacteria | __Bacteroidetes | __Sphingobacteriia | __Sphingobacteriales | __PHOS-HE51 | __uncultured_bacterium | 0.01 | 0 | 0.07 | 0.59 | 0.12 |
| Bacteria | __Planctomycetes | __OM190 | __uncultured_planctomycete | Other | Other | 0.01 | 0 | 0.07 | 1 | 0.12 |
| Bacteria | __Proteobacteria | __Gammaproteobacteria | __Alteromonadales | __Alteromonadaceae | __uncultured | 0 | 0.01 | 0.07 | 0.54 | 0.12 |
| Bacteria | __Proteobacteria | __Gammaproteobacteria | __Xanthomonadales | __Xanthomonadaceae | __Pseudoxanthomonas | 0.01 | 0 | 0.07 | 0.92 | 0.12 |
| Bacteria | __Chloroflexi | __Caldilineae | __Caldilineales | __Caldilineaceae | __Caldilinea | 0.01 | 0 | 0.07 | 0.93 | 0.12 |
| Bacteria | __Proteobacteria | __Deltaproteobacteria | __Myxococcales | __Nannocystineae | __Nannocystaceae | 0.01 | 0 | 0.07 | 1.05 | 0.12 |
| Bacteria | __Proteobacteria | __Alphaproteobacteria | __DB1-14 | __uncultured_alpha_proteobacterium | Other | 0.01 | 0.01 | 0.07 | 0.73 | 0.11 |
| Bacteria | __Actinobacteria | __Micrococcales | __Intrasporangiaceae | __Janibacter | Other | 0 | 0.01 | 0.07 | 0.54 | 0.11 |
| Bacteria | __Proteobacteria | __Alphaproteobacteria | __Rhodobacterales | __Rhodobacteraceae | __Loktanella | 0.01 | 0.01 | 0.07 | 1.02 | 0.11 |
| Bacteria | __Proteobacteria | __Alphaproteobacteria | __Caulobacterales | __Hyphomonadaceae | __Oceanicaulis | 0 | 0.01 | 0.07 | 0.44 | 0.11 |
| Bacteria | __Proteobacteria | __Alphaproteobacteria | __Caulobacterales | __Hyphomonadaceae | __Hyphomonas | 0.01 | 0.01 | 0.07 | 0.95 | 0.11 |
| Bacteria | __Armatimonadetes | __Chthonomonadetes | __Chthonomonadales | __uncultured_bacterium | Other | 0.01 | 0 | 0.07 | 0.44 | 0.11 |
| Bacteria | __Bacteroidetes | __Sphingobacteriia | __Sphingobacteriales | __Chitinophagaceae | __Segetibacter | 0.01 | 0 | 0.07 | 0.44 | 0.11 |
| Bacteria | __Proteobacteria | __Gammaproteobacteria | __Sva0071 | Other | Other | 0.01 | 0 | 0.07 | 0.67 | 0.11 |
| Bacteria | __Actinobacteria | Other | Other | Other | Other | 0.01 | 0 | 0.07 | 0.44 | 0.11 |
| Bacteria | __Bacteroidetes | __Flavobacteria | __Flavobacteriales | __Flavobacteriaceae | __Kordia | 0.01 | 0 | 0.07 | 0.44 | 0.11 |
| Bacteria | __Proteobacteria | __Gammaproteobacteria | __Xanthomonadales | __Xanthomonadaceae | Other | 0.01 | 0.01 | 0.07 | 0.78 | 0.11 |
| Bacteria | __Proteobacteria | __Gammaproteobacteria | __Thiotrichales | __EV818SWSAP88 | __uncultured_bacterium | 0 | 0.01 | 0.07 | 0.79 | 0.11 |
| Bacteria | __Proteobacteria | __Alphaproteobacteria | __Rhizobiales | __Rhizobiaceae | __Rhizobium | 0.01 | 0.01 | 0.07 | 0.8 | 0.11 |
| Bacteria | __Proteobacteria | __Alphaproteobacteria | __Rhodospirillales | __Rhodospirillaceae | __Azospirillum | 0.01 | 0.01 | 0.06 | 1.07 | 0.11 |
| Bacteria | __Proteobacteria | __Alphaproteobacteria | __Caulobacterales | __Hyphomonadaceae | Other | 0.01 | 0 | 0.06 | 0.8 | 0.11 |
| Bacteria | __Candidate_division_OD1 | __uncultured_bacterium | Other | Other | Other | 0.01 | 0.01 | 0.06 | 1.18 | 0.11 |
| Bacteria | __Planctomycetes | __OM190 | Other | Other | Other | 0.01 | 0.01 | 0.06 | 1.13 | 0.11 |
| Bacteria | __Proteobacteria | __Alphaproteobacteria | __Rickettsiales | Other | Other | 0.01 | 0 | 0.06 | 0.92 | 0.11 |
| Bacteria | __Proteobacteria | __Deltaproteobacteria | __Desulfuromonadales | __GR-WP33-58 | Other | 0.01 | 0.01 | 0.06 | 0.79 | 0.11 |
| Bacteria | __Proteobacteria | __Alphaproteobacteria | __Rhizobiales | __Hyphomicrobiaceae | __Devosia | 0.01 | 0.01 | 0.06 | 1.25 | 0.11 |
| Bacteria | __Planctomycetes | __Planctomycetacia | __Brocadiales | __Brocadiaceae | __Candidatus_Brocadia | 0 | 0.01 | 0.06 | 0.73 | 0.11 |
| Bacteria | __Bacteroidetes | __Cytophagia | __Cytophagales | __Flammeovirgaceae | Other | 0.01 | 0 | 0.06 | 0.86 | 0.11 |
| Bacteria | __Cyanobacteria | __SubsectionI | __FamilyI | Other | Other | 0.01 | 0 | 0.06 | 0.88 | 0.11 |
| Bacteria | __Acidobacteria | __Holophagae | __TK85 | Other | Other | 0.01 | 0 | 0.06 | 1.21 | 0.11 |
| Bacteria | __Verrucomicrobia | __Verrucomicrobiae | __Verrucomicrobiales | __Verrucomicrobiaceae | __Roseibacillus | 0.01 | 0.01 | 0.06 | 0.91 | 0.11 |
| Bacteria | __Proteobacteria | __Betaproteobacteria | __Burkholderiales | __Burkholderiaceae | __Ralstonia | 0.01 | 0 | 0.06 | 0.44 | 0.1 |
| Bacteria | __Chloroflexi | __TK10 | __uncultured_bacterium | Other | Other | 0.01 | 0 | 0.06 | 0.44 | 0.1 |
| Bacteria | __Bacteroidetes | __Sphingobacteriia | __Sphingobacteriales | __Chitinophagaceae | __Ferruginibacter | 0.01 | 0 | 0.06 | 0.79 | 0.1 |
| Bacteria | __Proteobacteria | __Gammaproteobacteria | __Enterobacteriales | __Enterobacteriaceae | __Proteus | 0.01 | 0.01 | 0.06 | 0.62 | 0.1 |
| Bacteria | __Cyanobacteria | __SubsectionIII | __FamilyI | __Planktothrix | Other | 0.01 | 0.01 | 0.06 | 0.81 | 0.1 |
| Bacteria | __Proteobacteria | __Candidatus_Allobeggiatoa | Other | Other | Other | 0.01 | 0 | 0.06 | 0.98 | 0.1 |
| Bacteria | __Proteobacteria | __Gammaproteobacteria | __Thiotrichales | __Family_Incertae_Sedis | __Caedibacter | 0 | 0.01 | 0.06 | 0.79 | 0.1 |
| Bacteria | __Proteobacteria | __Alphaproteobacteria | __SB1-18 | __uncultured_bacterium | Other | 0.01 | 0 | 0.06 | 0.64 | 0.1 |
| Bacteria | __Proteobacteria | __Deltaproteobacteria | __Syntrophobacterales | __Syntrophaceae | __Syntrophus | 0.01 | 0 | 0.06 | 0.75 | 0.1 |
| Bacteria | __Firmicutes | __Clostridia | __Clostridiales | __Family_XI_Incertae_Sedis | __Finegoldia | 0 | 0.01 | 0.06 | 0.81 | 0.1 |
| Bacteria | __Verrucomicrobia | __Verrucomicrobiae | __Verrucomicrobiales | __DEV007 | Other | 0.01 | 0 | 0.06 | 0.95 | 0.1 |
| Bacteria | __Bacteroidetes | __Cytophagia | __Cytophagales | __Flammeovirgaceae | __Candidatus_Amoebophilus | 0.01 | 0.01 | 0.06 | 0.94 | 0.1 |
| Bacteria | __Proteobacteria | __Deltaproteobacteria | __Bdellovibrionales | __Bdellovibrionaceae | __OM27_clade | 0.01 | 0 | 0.06 | 1.02 | 0.1 |
| Bacteria | __Proteobacteria | __TA18 | Other | Other | Other | 0.01 | 0 | 0.06 | 0.67 | 0.1 |
| Bacteria | __Cyanobacteria | __SubsectionIII | __FamilyI | __Lyngbya | Other | 0 | 0.01 | 0.06 | 0.6 | 0.1 |
| Bacteria | __Firmicutes | __Bacilli | __Bacillales | __Bacillaceae | __Bacillus | 0.01 | 0.01 | 0.06 | 0.77 | 0.1 |
| Bacteria | __Firmicutes | __Bacilli | __Bacillales | __Paenibacillaceae | __Paenibacillus | 0.01 | 0 | 0.06 | 0.54 | 0.1 |
| Bacteria | __Acidobacteria | __Holophagae | __CA002 | Other | Other | 0.01 | 0.01 | 0.06 | 0.8 | 0.1 |
| Bacteria | __Cyanobacteria | __SM2F09 | Other | Other | Other | 0.01 | 0 | 0.06 | 0.44 | 0.1 |
| Bacteria | __Acidobacteria | __Holophagae | __CA002 | __uncultured_bacterium | Other | 0 | 0.01 | 0.06 | 0.66 | 0.1 |
| Bacteria | __Proteobacteria | __Gammaproteobacteria | __Chromatiales | __Ectothiorhodospiraceae | __Thioalkalispira | 0 | 0.01 | 0.06 | 0.44 | 0.1 |
| Bacteria | __Cyanobacteria | __SubsectionIV | __FamilyII | __Rivularia | __Nostocales | 0 | 0.01 | 0.06 | 1.06 | 0.09 |
| Bacteria | __Proteobacteria | __Alphaproteobacteria | __Sphingomonadales | __Erythrobacteraceae | __Altererythrobacter | 0.01 | 0.01 | 0.06 | 0.78 | 0.09 |
| Bacteria | __Actinobacteria | __Micrococcales | __Bogoriellaceae | __Georgenia | __uncultured_bacterium | 0.01 | 0 | 0.06 | 0.44 | 0.09 |
| Bacteria | __Proteobacteria | __Alphaproteobacteria | __Rhizobiales | __Brucellaceae | __Daeguia | 0.01 | 0 | 0.06 | 0.44 | 0.09 |
| Bacteria | __Chlamydiae | __Chlamydiales | __Chlamydiaceae | __uncultured_Chlamydiales_bacterium | Other | 0.01 | 0 | 0.06 | 0.81 | 0.09 |
| Bacteria | __Chlamydiae | __Chlamydiales | __Simkaniaceae | __Candidatus_Rhabdochlamydia | __Chlamydiales | 0.01 | 0 | 0.06 | 0.69 | 0.09 |
| Bacteria | __Proteobacteria | __Alphaproteobacteria | __Rhodobacterales | __Rhodobacteraceae | __Oceanicola | 0.01 | 0 | 0.06 | 0.82 | 0.09 |
| Bacteria | __Spirochaetes | __Spirochaetales | __Leptospiraceae | __Turneriella | __uncultured_bacterium | 0.01 | 0 | 0.05 | 0.98 | 0.09 |
| Bacteria | __Actinobacteria | __Acidimicrobiia | __Acidimicrobiales | __OCS155_marine_group | Other | 0.01 | 0 | 0.05 | 0.7 | 0.09 |
| Bacteria | __Proteobacteria | __Alphaproteobacteria | __Rickettsiales | __Candidatus_Odyssella | Other | 0 | 0.01 | 0.05 | 0.82 | 0.09 |
| Bacteria | __Bacteroidetes | __WCHB1-32 | __uncultured_bacterium | Other | Other | 0.01 | 0.01 | 0.05 | 1.03 | 0.09 |
| Bacteria | __Cyanobacteria | __MLE1-12 | __uncultured_bacterium | Other | Other | 0.01 | 0 | 0.05 | 0.75 | 0.09 |
| Bacteria | __Bacteroidetes | __Sphingobacteriia | __Sphingobacteriales | __NS11-12_marine_group | Other | 0.01 | 0 | 0.05 | 0.97 | 0.09 |
| Bacteria | __Firmicutes | __Bacilli | __Bacillales | __Family_XI_Incertae_Sedis | __Gemella | 0.01 | 0 | 0.05 | 0.74 | 0.09 |
| Bacteria | __Proteobacteria | __Gammaproteobacteria | __NKB5 | Other | Other | 0.01 | 0.01 | 0.05 | 0.92 | 0.09 |
| Bacteria | __Actinobacteria | __Micromonosporales | __Micromonosporaceae | __Phytohabitans | Other | 0.01 | 0 | 0.05 | 0.44 | 0.09 |
| Bacteria | __Chloroflexi | __JG37-AG-4 | __uncultured_bacterium | Other | Other | 0.01 | 0 | 0.05 | 0.44 | 0.09 |
| Bacteria | __Proteobacteria | __Alphaproteobacteria | __Rhodobacterales | __Rhodobacteraceae | __Dinoroseobacter | 0.01 | 0 | 0.05 | 0.77 | 0.09 |
| Bacteria | __Proteobacteria | __SC3-20 | __uncultured_gamma_proteobacterium | Other | Other | 0.01 | 0 | 0.05 | 1.07 | 0.09 |
| Bacteria | __Proteobacteria | __Alphaproteobacteria | __Rhizobiales | __Brucellaceae | Other | 0.01 | 0 | 0.05 | 0.93 | 0.09 |
| Bacteria | __Bacteroidetes | __Flavobacteria | __Flavobacteriales | __Cryomorphaceae | __Crocinitomix | 0 | 0 | 0.05 | 0.77 | 0.09 |
| Bacteria | __Bacteroidetes | __Cytophagia | __Cytophagales | __Flammeovirgaceae | __uncultured_deep-sea_bacterium | 0 | 0.01 | 0.05 | 0.59 | 0.09 |
| Bacteria | __Proteobacteria | __Gammaproteobacteria | __Oceanospirillales | __Oceanospirillaceae | __Pseudospirillum | 0.01 | 0 | 0.05 | 1.04 | 0.09 |
| Bacteria | __Verrucomicrobia | __Verrucomicrobiae | __Verrucomicrobiales | __Verrucomicrobiaceae | __Luteolibacter | 0.01 | 0 | 0.05 | 0.6 | 0.09 |
| Bacteria | __Proteobacteria | __Deltaproteobacteria | __Sh765B-TzT-29 | __uncultured_delta_proteobacterium | Other | 0.01 | 0.01 | 0.05 | 0.79 | 0.09 |
| Bacteria | __Proteobacteria | __Alphaproteobacteria | __Rhodospirillales | __MSB-1E8 | Other | 0.01 | 0 | 0.05 | 0.78 | 0.08 |
| Bacteria | __Proteobacteria | __Skagenf62 | __uncultured_bacterium | Other | Other | 0.01 | 0.01 | 0.05 | 0.91 | 0.08 |
| Bacteria | __Proteobacteria | __Alphaproteobacteria | __Rhodobacterales | __Rhodobacteraceae | __Sulfitobacter | 0.01 | 0 | 0.05 | 0.56 | 0.08 |
| Bacteria | __Proteobacteria | __Gammaproteobacteria | __Order_Incertae_Sedis | __Family_Incertae_Sedis | Other | 0.01 | 0 | 0.05 | 0.63 | 0.08 |
| Bacteria | __Cyanobacteria | __SubsectionIII | __FamilyI | __Oscillatoria | Other | 0 | 0.01 | 0.05 | 0.79 | 0.08 |
| Bacteria | __Proteobacteria | __Alphaproteobacteria | __Rhodospirillales | __DA111 | Other | 0 | 0.01 | 0.05 | 0.44 | 0.08 |
| Bacteria | __Proteobacteria | __Gammaproteobacteria | __Oceanospirillales | __Oceanospirillaceae | __Oceanospirillum | 0 | 0.01 | 0.05 | 0.44 | 0.08 |
| Bacteria | __Proteobacteria | __Alphaproteobacteria | __Rickettsiales | __SM2D12 | Other | 0.01 | 0 | 0.05 | 0.68 | 0.08 |
| Bacteria | __Firmicutes | __Clostridia | __Clostridiales | __Veillonellaceae | __Veillonella | 0.01 | 0 | 0.05 | 0.59 | 0.08 |
| Bacteria | __Bacteroidetes | __Cytophagia | __Cytophagales | __Flammeovirgaceae | __Reichenbachiella | 0.01 | 0 | 0.05 | 0.77 | 0.08 |
| Bacteria | __Planctomycetes | __Phycisphaerae | __Phycisphaerales | __Phycisphaeraceae | __Urania-1B-19_marine_sediment_group | 0.01 | 0.01 | 0.05 | 0.89 | 0.08 |
| Bacteria | __Proteobacteria | __Alphaproteobacteria | __Rhizobiales | __Phyllobacteriaceae | __Aquamicrobium | 0.01 | 0 | 0.05 | 0.93 | 0.08 |
| Bacteria | __Proteobacteria | __Gammaproteobacteria | __Alteromonadales | __Alteromonadaceae | __Porticoccus | 0.01 | 0 | 0.05 | 0.52 | 0.08 |
| Bacteria | __Proteobacteria | __Deltaproteobacteria | __Desulfobacterales | __Desulfobacteraceae | __Desulfobacula | 0.01 | 0 | 0.05 | 0.77 | 0.08 |
| Bacteria | __Proteobacteria | __Gammaproteobacteria | __Enterobacteriales | __Enterobacteriaceae | __Salmonella | 0.01 | 0 | 0.05 | 0.77 | 0.08 |
| Bacteria | __Proteobacteria | __Alphaproteobacteria | __E6aD10 | Other | Other | 0.01 | 0 | 0.05 | 0.78 | 0.08 |
| Bacteria | __Firmicutes | __Clostridia | __Clostridiales | __Lachnospiraceae | Other | 0 | 0.01 | 0.05 | 0.53 | 0.08 |
| Bacteria | __Actinobacteria | __Pseudonocardiales | __Pseudonocardiaceae | __Pseudonocardia | Other | 0.01 | 0 | 0.04 | 1.01 | 0.08 |
| Bacteria | __Actinobacteria | __Micrococcales | __Micrococcaceae | __Micrococcus | __Micrococcus_luteus | 0.01 | 0 | 0.04 | 0.64 | 0.08 |
| Bacteria | __Verrucomicrobia | __Spartobacteria | __Chthoniobacterales | __DA101_soil_group | Other | 0 | 0 | 0.04 | 0.61 | 0.08 |
| Bacteria | __Actinobacteria | __Micrococcales | __Micrococcaceae | __Rothia | Other | 0.01 | 0 | 0.04 | 0.44 | 0.08 |
| Bacteria | __Candidate_division_WS3 | __uncultured_bacterium | Other | Other | Other | 0.01 | 0.01 | 0.04 | 1.06 | 0.07 |
| Bacteria | __Bacteroidetes | __Flavobacteria | __Flavobacteriales | __Cryomorphaceae | __NS7_marine_group | 0.01 | 0 | 0.04 | 0.61 | 0.07 |
| Bacteria | __Chloroflexi | __JG30-KF-CM66 | __uncultured_Chloroflexi_bacterium | Other | Other | 0.01 | 0 | 0.04 | 0.75 | 0.07 |
| Bacteria | __Firmicutes | __Clostridia | __Clostridiales | __Christensenellaceae | __uncultured | 0.01 | 0 | 0.04 | 0.53 | 0.07 |
| Bacteria | __Firmicutes | __Clostridia | __Clostridiales | __Family_XI_Incertae_Sedis | __Peptoniphilus | 0 | 0 | 0.04 | 0.76 | 0.07 |
| Bacteria | __Firmicutes | __Bacilli | __Bacillales | Other | Other | 0.01 | 0 | 0.04 | 0.44 | 0.07 |
| Bacteria | __Firmicutes | __Bacilli | __Lactobacillales | __Carnobacteriaceae | __Atopococcus | 0.01 | 0 | 0.04 | 0.44 | 0.07 |
| Bacteria | __NPL-UPA2 | __uncultured_bacterium | Other | Other | Other | 0.01 | 0 | 0.04 | 0.69 | 0.07 |
| Bacteria | __Proteobacteria | __Gammaproteobacteria | __Vibrionales | __Vibrionaceae | __Photobacterium | 0 | 0.01 | 0.04 | 0.69 | 0.07 |
| Bacteria | __Proteobacteria | __Gammaproteobacteria | __Chromatiales | Other | Other | 0.01 | 0 | 0.04 | 0.73 | 0.07 |
| Bacteria | __Proteobacteria | __Gammaproteobacteria | __HOC36 | __uncultured_bacterium | Other | 0.01 | 0 | 0.04 | 0.66 | 0.07 |
| Bacteria | __Firmicutes | __Bacilli | __Bacillales | __Bacillaceae | __Geobacillus | 0.01 | 0 | 0.04 | 0.44 | 0.07 |
| Bacteria | __Proteobacteria | __Alphaproteobacteria | __Rhizobiales | __Xanthobacteraceae | Other | 0.01 | 0 | 0.04 | 0.44 | 0.07 |
| Bacteria | __TM6 | Other | Other | Other | Other | 0.01 | 0 | 0.04 | 0.59 | 0.07 |
| Bacteria | __Acidobacteria | __Holophagae | __TK85 | __uncultured_bacterium | Other | 0.01 | 0 | 0.04 | 0.79 | 0.07 |

1. *Acropora millepora* and *Turbinaria reniformis* (average dissimilarity 71.26)

|  |  |  |  |  |  | **Avg. Abund.** | **Avg. Abund.** |  |  |  |  |
| --- | --- | --- | --- | --- | --- | --- | --- | --- | --- | --- | --- |
| **Operational Taxonomic Unit** | | | | | | **A.millepora** | **T.reniformis** | **Avg.**  **Diss.** | **Diss/**  **SD** | **%Contrib** | **%Cum.** |
| Bacteria | __Proteobacteria | __Alphaproteobacteria | __Sphingomonadales | __Sphingomonadaceae | __Sphingomonas | 0.32 | 0.43 | 1.81 | 1.26 | 2.54 | 2.54 |
| Bacteria | __Firmicutes | __Clostridia | __Halanaerobiales | __Halanaerobiaceae | __Halanaerobium | 0.15 | 0.16 | 1.48 | 0.77 | 2.08 | 4.62 |
| Bacteria | __Proteobacteria | __Alphaproteobacteria | __Rhodobacterales | __Rhodobacteraceae | __Ruegeria | 0.12 | 0.25 | 1.4 | 1.32 | 1.96 | 6.59 |
| Bacteria | __Proteobacteria | __Gammaproteobacteria | __Pseudomonadales | __Pseudomonadaceae | __Pseudomonas | 0.23 | 0.17 | 1.27 | 0.69 | 1.78 | 8.37 |
| Bacteria | __Proteobacteria | __Alphaproteobacteria | __Rhodobacterales | __Rhodobacteraceae | Other | 0.1 | 0.23 | 1.09 | 1.6 | 1.53 | 9.9 |
| Bacteria | __Proteobacteria | __Gammaproteobacteria | __Pseudomonadales | __Moraxellaceae | __Acinetobacter | 0.08 | 0.12 | 0.89 | 0.82 | 1.24 | 11.15 |
| Bacteria | __Actinobacteria | __Corynebacteriales | __Nocardiaceae | __Rhodococcus | __Rhodococcus_fascians | 0.11 | 0.08 | 0.73 | 0.89 | 1.03 | 12.18 |
| Bacteria | __Firmicutes | __Bacilli | __Bacillales | __Staphylococcaceae | __Staphylococcus | 0.13 | 0.05 | 0.66 | 1.15 | 0.92 | 13.1 |
| Bacteria | __Proteobacteria | __Alphaproteobacteria | __Rhizobiales | __Methylobacteriaceae | __Methylobacterium | 0.14 | 0.13 | 0.62 | 1.15 | 0.87 | 13.96 |
| Bacteria | __Proteobacteria | __Alphaproteobacteria | __Rhodobacterales | __Rhodobacteraceae | __Pseudovibrio | 0.06 | 0.04 | 0.61 | 0.47 | 0.85 | 14.81 |
| Bacteria | __Verrucomicrobia | __Spartobacteria | __Chthoniobacterales | __DA101_soil_group | __uncultured_bacterium | 0.1 | 0.05 | 0.59 | 1.1 | 0.82 | 15.64 |
| Bacteria | __Bacteroidetes | __Cytophagia | __Cytophagales | __Flammeovirgaceae | __Persicobacter | 0.01 | 0.09 | 0.55 | 0.87 | 0.77 | 16.41 |
| Bacteria | __Proteobacteria | __Alphaproteobacteria | Other | Other | Other | 0.03 | 0.09 | 0.51 | 0.77 | 0.72 | 17.12 |
| Bacteria | __Proteobacteria | __Alphaproteobacteria | __Caulobacterales | __Caulobacteraceae | __uncultured | 0.02 | 0.07 | 0.51 | 1.24 | 0.71 | 17.84 |
| Unassigned | Other | Other | Other | Other | Other | 0.1 | 0.14 | 0.47 | 1.2 | 0.66 | 18.5 |
| Bacteria | __Proteobacteria | __Alphaproteobacteria | __Rhodobacterales | __Rhodobacteraceae | __uncultured | 0.03 | 0.08 | 0.46 | 1.51 | 0.65 | 19.15 |
| Bacteria | __Proteobacteria | __Alphaproteobacteria | __Caulobacterales | __Hyphomonadaceae | __uncultured | 0.01 | 0.07 | 0.45 | 1.47 | 0.64 | 19.78 |
| Bacteria | __Proteobacteria | __Alphaproteobacteria | __Rhizobiales | __Rhodobiaceae | __Rhodobium | 0.03 | 0.09 | 0.44 | 1.57 | 0.62 | 20.4 |
| Bacteria | __Firmicutes | __Clostridia | __Clostridiales | __Clostridiaceae | __Clostridium | 0.08 | 0.03 | 0.43 | 1.24 | 0.6 | 21.01 |
| Archaea | __Thaumarchaeota | __Soil_Crenarchaeotic_Group(SCG) | __uncultured_archaeon | Other | Other | 0.06 | 0.02 | 0.43 | 0.75 | 0.6 | 21.6 |
| Bacteria | __Proteobacteria | __Deltaproteobacteria | __Myxococcales | __Sorangiineae | __Sandaracinaceae | 0.01 | 0.07 | 0.42 | 1.36 | 0.59 | 22.2 |
| Bacteria | __Cyanobacteria | __SubsectionIV | __FamilyII | __Rivularia | Other | 0 | 0.06 | 0.4 | 1.28 | 0.57 | 22.76 |
| Bacteria | __Proteobacteria | __Alphaproteobacteria | __Rhizobiales | __Hyphomicrobiaceae | __Filomicrobium | 0.06 | 0.09 | 0.39 | 1.44 | 0.54 | 23.31 |
| Bacteria | __Proteobacteria | __Gammaproteobacteria | __Legionellales | __Coxiellaceae | __Coxiella | 0.03 | 0.08 | 0.38 | 1.1 | 0.54 | 23.84 |
| Bacteria | __Proteobacteria | __Alphaproteobacteria | __Rhizobiales | __Xanthobacteraceae | __uncultured | 0.05 | 0.02 | 0.38 | 0.77 | 0.53 | 24.37 |
| Bacteria | __Chlamydiae | __Chlamydiales | __Parachlamydiaceae | __Neochlamydia | Other | 0.05 | 0.03 | 0.38 | 0.96 | 0.53 | 24.9 |
| Bacteria | __Proteobacteria | __Deltaproteobacteria | __GR-WP33-30 | __uncultured_bacterium | Other | 0.08 | 0.03 | 0.37 | 1.08 | 0.52 | 25.42 |
| Bacteria | __Proteobacteria | __Gammaproteobacteria | __Xanthomonadales | __Sinobacteraceae | __JTB255_marine_benthic_group | 0.01 | 0.06 | 0.35 | 1.28 | 0.49 | 25.92 |
| Bacteria | __Bacteroidetes | __Cytophagia | __Cytophagales | __Flammeovirgaceae | __uncultured | 0.02 | 0.06 | 0.35 | 1.18 | 0.49 | 26.4 |
| Bacteria | __Proteobacteria | __Gammaproteobacteria | __Oceanospirillales | __Hahellaceae | __Endozoicomonas | 0.05 | 0 | 0.34 | 0.78 | 0.48 | 26.89 |
| Bacteria | __Proteobacteria | __Alphaproteobacteria | __Rhizobiales | __Phyllobacteriaceae | __uncultured | 0.02 | 0.05 | 0.34 | 1.39 | 0.48 | 27.37 |
| Bacteria | __Acidobacteria | __32-21 | __uncultured_bacterium | Other | Other | 0.06 | 0.03 | 0.34 | 0.92 | 0.47 | 27.84 |
| Bacteria | __Acidobacteria | __DA052 | __uncultured_bacterium | Other | Other | 0.05 | 0.02 | 0.33 | 0.95 | 0.47 | 28.31 |
| Bacteria | __Bacteroidetes | __Sphingobacteriia | __Sphingobacteriales | __Saprospiraceae | __uncultured | 0.02 | 0.04 | 0.33 | 1.76 | 0.46 | 28.77 |
| Bacteria | __TM6 | __uncultured_bacterium | Other | Other | Other | 0.02 | 0.07 | 0.33 | 1.57 | 0.46 | 29.23 |
| Bacteria | __Actinobacteria | __Acidimicrobiia | __Acidimicrobiales | __OCS155_marine_group | __uncultured_bacterium | 0.02 | 0.06 | 0.32 | 1.22 | 0.44 | 29.67 |
| Bacteria | __Proteobacteria | __Gammaproteobacteria | __Xanthomonadales | __Sinobacteraceae | Other | 0 | 0.05 | 0.31 | 1.07 | 0.44 | 30.11 |
| Bacteria | __Firmicutes | __Clostridia | __Clostridiales | __Clostridiaceae | __Clostridiisalibacter | 0.03 | 0.01 | 0.31 | 0.46 | 0.44 | 30.55 |
| Bacteria | __Planctomycetes | __Planctomycetacia | __Planctomycetales | __Planctomycetaceae | __Pir4_lineage | 0.03 | 0.07 | 0.31 | 1.76 | 0.43 | 30.98 |
| Bacteria | __Proteobacteria | __Alphaproteobacteria | __Rhodospirillales | __Rhodospirillaceae | __uncultured | 0.03 | 0.05 | 0.3 | 1.43 | 0.43 | 31.41 |
| Bacteria | __Proteobacteria | __Gammaproteobacteria | __Oceanospirillales | Other | Other | 0 | 0.05 | 0.3 | 1.09 | 0.42 | 31.83 |
| Bacteria | __Proteobacteria | __Epsilonproteobacteria | __Campylobacterales | __Campylobacteraceae | __Campylobacter | 0.05 | 0 | 0.29 | 0.3 | 0.4 | 32.23 |
| Bacteria | __Proteobacteria | __Deltaproteobacteria | __Sh765B-TzT-29 | __uncultured_bacterium | Other | 0.02 | 0.04 | 0.29 | 1.22 | 0.4 | 32.63 |
| Bacteria | __Actinobacteria | __Acidimicrobiia | __Acidimicrobiales | __Sva0996_marine_group | Other | 0.01 | 0.04 | 0.28 | 1.08 | 0.39 | 33.03 |
| Bacteria | __Bacteroidetes | __Flavobacteria | __Flavobacteriales | __Flavobacteriaceae | __Flavobacterium | 0.01 | 0.04 | 0.28 | 1.17 | 0.39 | 33.41 |
| Bacteria | __Planctomycetes | __Planctomycetacia | __Planctomycetales | __Planctomycetaceae | __Rhodopirellula | 0.02 | 0.06 | 0.27 | 1.65 | 0.38 | 33.8 |
| Bacteria | __Planctomycetes | __Planctomycetacia | __Planctomycetales | __Planctomycetaceae | __Planctomyces | 0.03 | 0.05 | 0.27 | 1.43 | 0.38 | 34.18 |
| Bacteria | __Chlamydiae | __Chlamydiales | __Simkaniaceae | __Candidatus_Rhabdochlamydia | Other | 0.01 | 0.04 | 0.27 | 1.02 | 0.37 | 34.55 |
| Bacteria | __Proteobacteria | __Betaproteobacteria | __Burkholderiales | __Comamonadaceae | __Variovorax | 0.01 | 0.04 | 0.26 | 1.09 | 0.37 | 34.93 |
| Bacteria | __Proteobacteria | __Deltaproteobacteria | __Myxococcales | __Cystobacterineae | __uncultured | 0.03 | 0.02 | 0.26 | 1.02 | 0.37 | 35.3 |
| Bacteria | __Acidobacteria | __RB41 | __uncultured_bacterium | Other | Other | 0.03 | 0.01 | 0.26 | 0.71 | 0.37 | 35.66 |
| Bacteria | __Proteobacteria | __Betaproteobacteria | __Burkholderiales | __Oxalobacteraceae | __Massilia | 0.01 | 0.03 | 0.26 | 0.47 | 0.37 | 36.03 |
| Bacteria | __Proteobacteria | __Alphaproteobacteria | __Rhizobiales | __Phyllobacteriaceae | Other | 0.01 | 0.05 | 0.26 | 1.39 | 0.36 | 36.39 |
| Bacteria | __Verrucomicrobia | __OPB35_soil_group | __uncultured_bacterium | Other | Other | 0.04 | 0.01 | 0.26 | 0.85 | 0.36 | 36.75 |
| Bacteria | __Firmicutes | __Clostridia | __Clostridiales | __Family_XI_Incertae_Sedis | __Peptoniphilus | 0.04 | 0 | 0.26 | 0.45 | 0.36 | 37.11 |
| Bacteria | __Proteobacteria | __Deltaproteobacteria | __Desulfovibrionales | __Desulfovibrionaceae | __Desulfovibrio | 0.03 | 0.02 | 0.25 | 0.63 | 0.36 | 37.47 |
| Bacteria | __Proteobacteria | __Gammaproteobacteria | __Vibrionales | __Vibrionaceae | __Vibrio | 0.02 | 0.02 | 0.25 | 1.17 | 0.36 | 37.83 |
| Bacteria | __Proteobacteria | __Alphaproteobacteria | __OCS116_clade | Other | Other | 0.02 | 0.05 | 0.25 | 1.59 | 0.36 | 38.18 |
| Bacteria | __Planctomycetes | __Planctomycetacia | __Planctomycetales | __Planctomycetaceae | __Pirellula | 0.04 | 0.04 | 0.25 | 1.39 | 0.35 | 38.53 |
| Bacteria | __Verrucomicrobia | __Verrucomicrobiae | __Verrucomicrobiales | __DEV007 | __uncultured_bacterium | 0.02 | 0.04 | 0.25 | 1.2 | 0.35 | 38.88 |
| Bacteria | __Proteobacteria | __Alphaproteobacteria | __Parvularculales | __Parvularculaceae | __Parvularcula | 0 | 0.04 | 0.25 | 1.43 | 0.35 | 39.23 |
| Bacteria | __Proteobacteria | __Alphaproteobacteria | __Sphingomonadales | Other | Other | 0.04 | 0.02 | 0.25 | 0.5 | 0.35 | 39.58 |
| Bacteria | __Proteobacteria | __Gammaproteobacteria | __Alteromonadales | __Alteromonadaceae | Other | 0.01 | 0.04 | 0.25 | 0.69 | 0.35 | 39.93 |
| Bacteria | __Proteobacteria | __TA18 | __uncultured_bacterium | Other | Other | 0.02 | 0.05 | 0.25 | 1.49 | 0.34 | 40.28 |
| Bacteria | __Proteobacteria | __Gammaproteobacteria | __Legionellales | __Coxiellaceae | __Aquicella | 0.01 | 0.04 | 0.24 | 1.04 | 0.34 | 40.62 |
| Bacteria | __Firmicutes | __Clostridia | __Clostridiales | __Family_XI_Incertae_Sedis | __Finegoldia | 0.04 | 0.01 | 0.24 | 0.56 | 0.34 | 40.96 |
| Bacteria | __Firmicutes | __Bacilli | __Bacillales | __Bacillaceae | __Bacillus | 0.04 | 0.01 | 0.24 | 0.74 | 0.34 | 41.3 |
| Bacteria | __Proteobacteria | __Gammaproteobacteria | __Chromatiales | __Granulosicoccaceae | __Granulosicoccus | 0 | 0.04 | 0.24 | 1.06 | 0.34 | 41.63 |
| Bacteria | __Proteobacteria | __Betaproteobacteria | __Hydrogenophilales | __Hydrogenophilaceae | __Hydrogenophilus | 0.03 | 0 | 0.24 | 0.41 | 0.33 | 41.97 |
| Bacteria | __Proteobacteria | __Deltaproteobacteria | __Myxococcales | __Nannocystineae | __Haliangiaceae | 0.03 | 0.02 | 0.24 | 0.7 | 0.33 | 42.3 |
| Bacteria | __Acidobacteria | __DA023 | __uncultured_bacterium | Other | Other | 0.03 | 0.02 | 0.23 | 0.77 | 0.33 | 42.63 |
| Bacteria | __Proteobacteria | __Deltaproteobacteria | __Myxococcales | __0319-6G20 | __uncultured_bacterium | 0.02 | 0.03 | 0.23 | 1.2 | 0.33 | 42.96 |
| Bacteria | __Proteobacteria | __Gammaproteobacteria | __NKB5 | __uncultured_bacterium | Other | 0.02 | 0.04 | 0.23 | 1.4 | 0.32 | 43.28 |
| Bacteria | __Firmicutes | __Bacilli | __Lactobacillales | __Streptococcaceae | __Streptococcus | 0.04 | 0.02 | 0.23 | 1.51 | 0.32 | 43.6 |
| Bacteria | __Planctomycetes | __Planctomycetacia | __Planctomycetales | __Planctomycetaceae | __Blastopirellula | 0.03 | 0.04 | 0.23 | 1.41 | 0.32 | 43.93 |
| Bacteria | __Proteobacteria | __Alphaproteobacteria | __Rhizobiales | Other | Other | 0.01 | 0.04 | 0.23 | 0.7 | 0.32 | 44.25 |
| Bacteria | __Actinobacteria | __Thermoleophilia | __Gaiellales | __uncultured | __uncultured_actinobacterium | 0.02 | 0.04 | 0.23 | 1.02 | 0.32 | 44.57 |
| Bacteria | __Actinobacteria | __Corynebacteriales | __Corynebacteriaceae | __Corynebacterium | Other | 0.03 | 0.02 | 0.23 | 1.13 | 0.32 | 44.89 |
| Bacteria | __Planctomycetes | __Planctomycetacia | __Planctomycetales | __Planctomycetaceae | __uncultured | 0.04 | 0.01 | 0.23 | 1.28 | 0.32 | 45.21 |
| Bacteria | __Acidobacteria | __Acidobacteriales | __Acidobacteriaceae | __uncultured | __uncultured_bacterium | 0.02 | 0.02 | 0.23 | 0.98 | 0.32 | 45.53 |
| Bacteria | __Bacteroidetes | __Sphingobacteriia | __Sphingobacteriales | __Chitinophagaceae | __uncultured | 0.04 | 0.03 | 0.23 | 1 | 0.32 | 45.85 |
| Bacteria | __Proteobacteria | __Gammaproteobacteria | Other | Other | Other | 0.02 | 0.04 | 0.23 | 1.35 | 0.32 | 46.16 |
| Bacteria | __Proteobacteria | __Gammaproteobacteria | __Alteromonadales | __Alteromonadaceae | __Haliea | 0.01 | 0.03 | 0.22 | 1.75 | 0.31 | 46.48 |
| Bacteria | __Nitrospirae | __Nitrospira | __Nitrospirales | __Nitrospiraceae | __Nitrospira | 0.03 | 0.01 | 0.22 | 0.99 | 0.31 | 46.79 |
| Bacteria | __Actinobacteria | __Thermoleophilia | __Gaiellales | __uncultured | __uncultured_bacterium | 0.03 | 0.02 | 0.22 | 1.02 | 0.31 | 47.1 |
| Bacteria | __Firmicutes | __Clostridia | __Clostridiales | __Family_XI_Incertae_Sedis | __Anaerococcus | 0.04 | 0.01 | 0.22 | 0.9 | 0.31 | 47.4 |
| Bacteria | __Proteobacteria | __Alphaproteobacteria | __Rhizobiales | __Rhodobiaceae | __Anderseniella | 0 | 0.04 | 0.22 | 1.18 | 0.3 | 47.71 |
| Bacteria | __Proteobacteria | __Alphaproteobacteria | __Rhizobiales | __Phyllobacteriaceae | __Ahrensia | 0.01 | 0.03 | 0.22 | 1.22 | 0.3 | 48.01 |
| Bacteria | __Proteobacteria | __Alphaproteobacteria | __Rhizobiales | __Phyllobacteriaceae | __Nitratireductor | 0.03 | 0.02 | 0.22 | 0.76 | 0.3 | 48.32 |
| Bacteria | __Verrucomicrobia | __Verrucomicrobiae | __Verrucomicrobiales | __Verrucomicrobiaceae | __Haloferula | 0.03 | 0.01 | 0.21 | 0.94 | 0.3 | 48.62 |
| Bacteria | __Chloroflexi | __Anaerolineae | __Anaerolineales | __Anaerolineaceae | __uncultured | 0.01 | 0.03 | 0.21 | 1.19 | 0.3 | 48.91 |
| Bacteria | __Proteobacteria | __Gammaproteobacteria | __Sva0071 | __uncultured_bacterium | Other | 0.01 | 0.03 | 0.21 | 0.85 | 0.29 | 49.21 |
| Bacteria | __Proteobacteria | __Gammaproteobacteria | __Enterobacteriales | __Enterobacteriaceae | __Enterobacter | 0.02 | 0.02 | 0.2 | 0.89 | 0.29 | 49.49 |
| Bacteria | __Proteobacteria | __Gammaproteobacteria | __Legionellales | __Legionellaceae | __uncultured | 0.04 | 0.05 | 0.2 | 0.73 | 0.29 | 49.78 |
| Bacteria | __Proteobacteria | __Alphaproteobacteria | __Rhodobacterales | __Rhodobacteraceae | __Stappia | 0.02 | 0.04 | 0.2 | 1.32 | 0.28 | 50.06 |
| Bacteria | __Proteobacteria | __Betaproteobacteria | __Burkholderiales | __Oxalobacteraceae | Other | 0.02 | 0.02 | 0.2 | 1.03 | 0.28 | 50.34 |
| Bacteria | __Proteobacteria | __Betaproteobacteria | __Nitrosomonadales | __Nitrosomonadaceae | __uncultured | 0.03 | 0.01 | 0.2 | 0.81 | 0.28 | 50.62 |
| Bacteria | __Proteobacteria | __Alphaproteobacteria | __Rhodobacterales | __Rhodobacteraceae | __Paracoccus | 0.02 | 0.02 | 0.2 | 0.94 | 0.28 | 50.91 |
| Bacteria | __Firmicutes | __Clostridia | __Clostridiales | __Lachnospiraceae | __uncultured | 0.01 | 0.02 | 0.2 | 0.51 | 0.28 | 51.18 |
| Bacteria | __Proteobacteria | __Alphaproteobacteria | __Rhodobacterales | __Rhodobacteraceae | __Rhodovulum | 0.01 | 0.03 | 0.2 | 1.36 | 0.28 | 51.46 |
| Bacteria | __Proteobacteria | __Alphaproteobacteria | __Sphingomonadales | __Erythrobacteraceae | Other | 0.01 | 0.03 | 0.19 | 1.35 | 0.27 | 51.73 |
| Bacteria | __Proteobacteria | __Gammaproteobacteria | __Order_Incertae_Sedis | __Family_Incertae_Sedis | __Marinicella | 0 | 0.03 | 0.19 | 0.53 | 0.27 | 52 |
| Bacteria | __Cyanobacteria | __SubsectionI | __FamilyI | __Synechococcus | __uncultured_Synechococcus_sp. | 0 | 0.03 | 0.19 | 0.59 | 0.27 | 52.27 |
| Bacteria | __Proteobacteria | __Gammaproteobacteria | __Pseudomonadales | __Moraxellaceae | __Enhydrobacter | 0.02 | 0.02 | 0.19 | 0.82 | 0.27 | 52.54 |
| Bacteria | __Proteobacteria | __Alphaproteobacteria | __Caulobacterales | __Caulobacteraceae | __Phenylobacterium | 0 | 0.02 | 0.19 | 0.92 | 0.27 | 52.81 |
| Bacteria | __Proteobacteria | __Alphaproteobacteria | __Sphingomonadales | __Sphingomonadaceae | Other | 0.01 | 0.03 | 0.19 | 0.84 | 0.26 | 53.07 |
| Bacteria | __Chloroflexi | __Caldilineae | __Caldilineales | __Caldilineaceae | __uncultured | 0.01 | 0.02 | 0.19 | 1.09 | 0.26 | 53.33 |
| Bacteria | __Proteobacteria | __Betaproteobacteria | __Burkholderiales | __Burkholderiaceae | __Cupriavidus | 0 | 0.02 | 0.18 | 0.62 | 0.26 | 53.59 |
| Bacteria | __Proteobacteria | __Alphaproteobacteria | __Rhizobiales | __Bradyrhizobiaceae | __Bradyrhizobium | 0.02 | 0.02 | 0.18 | 0.99 | 0.25 | 53.85 |
| Bacteria | __Cyanobacteria | __MLE1-12 | __uncultured_bacterium | Other | Other | 0.02 | 0 | 0.18 | 0.49 | 0.25 | 54.09 |
| Bacteria | __Proteobacteria | __Deltaproteobacteria | __Desulfobacterales | __Desulfobulbaceae | __uncultured | 0.02 | 0.01 | 0.18 | 0.55 | 0.25 | 54.34 |
| Bacteria | __Proteobacteria | __Alphaproteobacteria | __Rhodobacterales | __Rhodobacteraceae | __Roseobacter_clade_CHAB-I-5_lineage | 0.01 | 0.03 | 0.18 | 1.28 | 0.25 | 54.59 |
| Bacteria | __Proteobacteria | __Deltaproteobacteria | __Desulfobacterales | __Nitrospinaceae | __Candidatus_Entotheonella | 0.02 | 0.01 | 0.17 | 0.77 | 0.25 | 54.84 |
| Bacteria | __Verrucomicrobia | __Verrucomicrobiae | __Verrucomicrobiales | __DEV007 | __uncultured_Verrucomicrobia_bacterium | 0.01 | 0.03 | 0.17 | 1.24 | 0.24 | 55.08 |
| Bacteria | __Proteobacteria | __Alphaproteobacteria | __Rhizobiales | __Xanthobacteraceae | Other | 0.02 | 0 | 0.17 | 0.78 | 0.24 | 55.32 |
| Bacteria | __Firmicutes | __Bacilli | __Bacillales | __Family_XII_Incertae_Sedis | __Exiguobacterium | 0.02 | 0.01 | 0.17 | 0.69 | 0.24 | 55.56 |
| Bacteria | __Proteobacteria | __Gammaproteobacteria | __Xanthomonadales | __Sinobacteraceae | __uncultured | 0.01 | 0.02 | 0.17 | 1.07 | 0.23 | 55.79 |
| Bacteria | __Proteobacteria | __Deltaproteobacteria | __Bdellovibrionales | __Bdellovibrionaceae | __Bdellovibrio | 0.01 | 0.02 | 0.17 | 1.03 | 0.23 | 56.02 |
| Bacteria | __Candidate_division_WS3 | __uncultured_bacterium | Other | Other | Other | 0.02 | 0.01 | 0.16 | 0.51 | 0.23 | 56.25 |
| Bacteria | __Acidobacteria | __Candidatus_Solibacter | __uncultured_bacterium | Other | Other | 0.02 | 0 | 0.16 | 0.72 | 0.23 | 56.48 |
| Bacteria | __Proteobacteria | __Alphaproteobacteria | __Sphingomonadales | __Sphingomonadaceae | __Sphingopyxis | 0 | 0.03 | 0.16 | 1.17 | 0.23 | 56.71 |
| Bacteria | __Proteobacteria | __Alphaproteobacteria | __Rhodospirillales | __DA111 | __uncultured_bacterium | 0.02 | 0.01 | 0.16 | 0.59 | 0.23 | 56.94 |
| Bacteria | __Proteobacteria | __Betaproteobacteria | __Burkholderiales | __Comamonadaceae | Other | 0.02 | 0.01 | 0.16 | 0.73 | 0.23 | 57.17 |
| Bacteria | __Bacteroidetes | __Cytophagia | __Cytophagales | __Flammeovirgaceae | __Flexithrix | 0.01 | 0.02 | 0.16 | 1.19 | 0.22 | 57.39 |
| Bacteria | __Proteobacteria | __TA18 | __uncultured_delta_proteobacterium | Other | Other | 0 | 0.03 | 0.16 | 0.8 | 0.22 | 57.61 |
| Bacteria | __Cyanobacteria | __SubsectionI | __FamilyI | __uncultured_bacterium | Other | 0.01 | 0.02 | 0.16 | 1.02 | 0.22 | 57.83 |
| Bacteria | __Bacteroidetes | __Cytophagia | __Cytophagales | __Flammeovirgaceae | __Ekhidna | 0 | 0.02 | 0.16 | 0.87 | 0.22 | 58.05 |
| Bacteria | __Actinobacteria | __Corynebacteriales | __Nocardiaceae | __Rhodococcus | Other | 0.02 | 0.02 | 0.15 | 1.52 | 0.22 | 58.27 |
| Bacteria | __Acidobacteria | __Acidobacteriales | __Acidobacteriaceae | __uncultured | __uncultured_Acidobacteria_bacterium | 0.02 | 0 | 0.15 | 0.6 | 0.21 | 58.48 |
| Bacteria | __Proteobacteria | __Alphaproteobacteria | __DB1-14 | __uncultured_marine_bacterium | Other | 0.01 | 0.02 | 0.15 | 1.32 | 0.21 | 58.69 |
| Bacteria | __Bacteroidetes | __Cytophagia | __Cytophagales | __Flammeovirgaceae | __Fulvivirga | 0 | 0.02 | 0.15 | 0.92 | 0.21 | 58.9 |
| Bacteria | __Actinobacteria | __Corynebacteriales | __Corynebacteriaceae | __Corynebacterium | __uncultured_bacterium | 0.02 | 0.01 | 0.15 | 0.59 | 0.21 | 59.1 |
| Bacteria | __Bacteroidetes | __Sphingobacteriia | __Sphingobacteriales | __Chitinophagaceae | __Ferruginibacter | 0.02 | 0.01 | 0.15 | 0.43 | 0.21 | 59.31 |
| Bacteria | __Proteobacteria | __Alphaproteobacteria | __Rhizobiales | __MNG7 | __uncultured_bacterium | 0 | 0.02 | 0.15 | 0.94 | 0.2 | 59.51 |
| Bacteria | __Actinobacteria | __Acidimicrobiia | __Acidimicrobiales | __Sva0996_marine_group | __uncultured_actinobacterium | 0 | 0.02 | 0.15 | 1.06 | 0.2 | 59.72 |
| Bacteria | __Actinobacteria | __Acidimicrobiia | __Acidimicrobiales | __uncultured | __uncultured_bacterium | 0.01 | 0.02 | 0.15 | 0.94 | 0.2 | 59.92 |
| Bacteria | __Deinococcus-Thermus | __Deinococci | __Thermales | __Thermaceae | __Thermus | 0.02 | 0 | 0.14 | 0.59 | 0.2 | 60.12 |
| Bacteria | __Proteobacteria | Other | Other | Other | Other | 0 | 0.02 | 0.14 | 0.85 | 0.2 | 60.32 |
| Bacteria | __Proteobacteria | __Gammaproteobacteria | __KI89A_clade | Other | Other | 0.01 | 0.02 | 0.14 | 0.95 | 0.2 | 60.52 |
| Bacteria | __Proteobacteria | __Alphaproteobacteria | __Rhizobiales | __Phyllobacteriaceae | __Hoeflea | 0.01 | 0.02 | 0.14 | 1.23 | 0.2 | 60.72 |
| Bacteria | __Chlamydiae | __Chlamydiales | __Family_Incertae_Sedis | __Criblamydia | Other | 0.02 | 0 | 0.14 | 0.53 | 0.2 | 60.92 |
| Bacteria | __Proteobacteria | __Gammaproteobacteria | __Thiotrichales | __EV818SWSAP88 | __uncultured_gamma_proteobacterium | 0.01 | 0.02 | 0.14 | 1.04 | 0.2 | 61.11 |
| Bacteria | __Proteobacteria | __Alphaproteobacteria | __Caulobacterales | __Caulobacteraceae | __Brevundimonas | 0.01 | 0.01 | 0.14 | 0.64 | 0.2 | 61.31 |
| Bacteria | __Actinobacteria | __Acidimicrobiia | __Acidimicrobiales | __Sva0996_marine_group | __uncultured_bacterium | 0.01 | 0.02 | 0.14 | 0.99 | 0.2 | 61.51 |
| Bacteria | __Proteobacteria | __Alphaproteobacteria | __E6aD10 | __uncultured_Rhizobiales_bacterium | Other | 0 | 0.02 | 0.14 | 1.04 | 0.2 | 61.7 |
| Bacteria | __Firmicutes | __Clostridia | __Clostridiales | __Ruminococcaceae | __Ruminococcus | 0 | 0.02 | 0.14 | 0.56 | 0.19 | 61.9 |
| Bacteria | __Chlamydiae | __Chlamydiales | __Simkaniaceae | Other | Other | 0.01 | 0.01 | 0.14 | 0.77 | 0.19 | 62.09 |
| Bacteria | __Proteobacteria | __Alphaproteobacteria | __Rhizobiales | __Phyllobacteriaceae | __Cohaesibacter | 0.01 | 0.02 | 0.14 | 1.14 | 0.19 | 62.28 |
| Bacteria | __Acidobacteria | __11-24 | __uncultured_bacterium | Other | Other | 0.01 | 0.01 | 0.14 | 0.63 | 0.19 | 62.47 |
| Bacteria | __Planctomycetes | __OM190 | __uncultured_bacterium | Other | Other | 0.01 | 0.02 | 0.14 | 1.14 | 0.19 | 62.66 |
| Bacteria | __Proteobacteria | __Deltaproteobacteria | __GR-WP33-30 | __uncultured_delta_proteobacterium | Other | 0.02 | 0.01 | 0.13 | 0.58 | 0.19 | 62.85 |
| Bacteria | __Spirochaetes | __Spirochaetales | __Leptospiraceae | __uncultured | __uncultured_bacterium | 0 | 0.02 | 0.13 | 1.12 | 0.19 | 63.03 |
| Bacteria | __Firmicutes | __Bacilli | __Bacillales | __Alicyclobacillaceae | __Alicyclobacillus | 0.02 | 0 | 0.13 | 0.52 | 0.18 | 63.21 |
| Bacteria | __Proteobacteria | __Betaproteobacteria | __Burkholderiales | __Alcaligenaceae | __Alcaligenes | 0.02 | 0 | 0.13 | 0.48 | 0.18 | 63.39 |
| Bacteria | __Firmicutes | __Bacilli | __Lactobacillales | __Lactobacillaceae | __Lactobacillus | 0.02 | 0 | 0.13 | 0.75 | 0.18 | 63.57 |
| Bacteria | __Bacteroidetes | __Flavobacteria | __Flavobacteriales | __Flavobacteriaceae | Other | 0 | 0.02 | 0.13 | 1.06 | 0.18 | 63.75 |
| Bacteria | __Firmicutes | __Clostridia | __Clostridiales | __Peptostreptococcaceae | __uncultured | 0.01 | 0.01 | 0.13 | 0.48 | 0.18 | 63.93 |
| Bacteria | __Bacteroidetes | __Cytophagia | __Order_II_Incertae_Sedis | __Rhodothermaceae | __Rubricoccus | 0 | 0.02 | 0.13 | 1.03 | 0.18 | 64.1 |
| Bacteria | __Actinobacteria | __Frankiales | __Acidothermaceae | __Acidothermus | __uncultured_bacterium | 0.02 | 0.01 | 0.12 | 0.67 | 0.17 | 64.28 |
| Bacteria | __Cyanobacteria | __SubsectionII | __FamilyII | __Chroococcidiopsis | __uncultured_bacterium | 0.02 | 0 | 0.12 | 0.3 | 0.17 | 64.45 |
| Bacteria | __Acidobacteria | __DA023 | __uncultured_Acidobacteria_bacterium | Other | Other | 0.02 | 0.01 | 0.12 | 0.61 | 0.17 | 64.62 |
| Bacteria | __Bacteroidetes | __Sphingobacteriia | __Sphingobacteriales | __Saprospiraceae | __Lewinella | 0 | 0.02 | 0.12 | 0.67 | 0.17 | 64.79 |
| Bacteria | __Proteobacteria | __Gammaproteobacteria | __E01-9C-26_marine_group | __uncultured_gamma_proteobacterium | Other | 0 | 0.02 | 0.12 | 0.93 | 0.17 | 64.96 |
| Bacteria | __Chlamydiae | __Chlamydiales | __Parachlamydiaceae | Other | Other | 0.01 | 0.01 | 0.12 | 1.11 | 0.17 | 65.12 |
| Bacteria | __Proteobacteria | __Alphaproteobacteria | __Rhizobiales | __Hyphomicrobiaceae | Other | 0 | 0.02 | 0.12 | 1.38 | 0.17 | 65.29 |
| Bacteria | __Actinobacteria | __Corynebacteriales | __Mycobacteriaceae | __Mycobacterium | Other | 0.01 | 0.01 | 0.12 | 0.99 | 0.16 | 65.45 |
| Bacteria | __Actinobacteria | __Acidimicrobiia | __Acidimicrobiales | Other | Other | 0 | 0.02 | 0.12 | 1.08 | 0.16 | 65.62 |
| Bacteria | __Proteobacteria | __Gammaproteobacteria | __Alteromonadales | __Colwelliaceae | __Thalassomonas | 0 | 0.02 | 0.12 | 0.35 | 0.16 | 65.78 |
| Bacteria | __Proteobacteria | __Gammaproteobacteria | __Alteromonadales | __Alteromonadaceae | __Alteromonas | 0 | 0.02 | 0.12 | 0.51 | 0.16 | 65.95 |
| Bacteria | __Cyanobacteria | __SubsectionII | __FamilyI | __Xenococcus | Other | 0.01 | 0.01 | 0.12 | 0.94 | 0.16 | 66.11 |
| Bacteria | __Cyanobacteria | __SubsectionI | __FamilyI | __Synechococcus | Other | 0 | 0.02 | 0.12 | 0.96 | 0.16 | 66.27 |
| Bacteria | __Proteobacteria | __Deltaproteobacteria | __Syntrophobacterales | __Syntrophaceae | __uncultured | 0 | 0.02 | 0.11 | 0.8 | 0.15 | 66.42 |
| Bacteria | __Actinobacteria | __Micrococcales | __Microbacteriaceae | Other | Other | 0.01 | 0.01 | 0.11 | 0.54 | 0.15 | 66.58 |
| Bacteria | __Proteobacteria | __Alphaproteobacteria | __Rhizobiales | __Methylobacteriaceae | __Microvirga | 0.02 | 0 | 0.11 | 0.36 | 0.15 | 66.73 |
| Bacteria | __Cyanobacteria | __4C0d-2 | __uncultured_bacterium | Other | Other | 0 | 0.01 | 0.11 | 0.41 | 0.15 | 66.89 |
| Bacteria | __Proteobacteria | __Alphaproteobacteria | __Sphingomonadales | __Sphingomonadaceae | __Sphingobium | 0.01 | 0.01 | 0.11 | 0.84 | 0.15 | 67.04 |
| Bacteria | __Proteobacteria | __Deltaproteobacteria | __Myxococcales | __Nannocystineae | __uncultured | 0.01 | 0.01 | 0.11 | 0.79 | 0.15 | 67.19 |
| Bacteria | __Proteobacteria | __Alphaproteobacteria | __Rhodospirillales | __Rhodospirillaceae | __Defluviicoccus | 0 | 0.02 | 0.11 | 0.88 | 0.15 | 67.34 |
| Bacteria | __Proteobacteria | __Gammaproteobacteria | __Xanthomonadales | __Sinobacteraceae | __Nevskia | 0 | 0.02 | 0.11 | 0.62 | 0.15 | 67.49 |
| Bacteria | __Proteobacteria | __Alphaproteobacteria | __Rhodobacterales | __Rhodobacteraceae | __Labrenzia | 0.01 | 0.01 | 0.11 | 1.12 | 0.15 | 67.64 |
| Bacteria | __Proteobacteria | __Gammaproteobacteria | __Legionellales | __Coxiellaceae | Other | 0.01 | 0.01 | 0.11 | 0.85 | 0.15 | 67.79 |
| Bacteria | __Cyanobacteria | __SubsectionIII | __FamilyI | __Lyngbya | __uncultured_bacterium | 0 | 0.01 | 0.1 | 0.69 | 0.15 | 67.93 |
| Bacteria | __Proteobacteria | __Deltaproteobacteria | __GR-WP33-30 | Other | Other | 0 | 0.02 | 0.1 | 0.89 | 0.15 | 68.08 |
| Bacteria | __Proteobacteria | __Gammaproteobacteria | __Oceanospirillales | __Alcanivoracaceae | __Alcanivorax | 0.01 | 0.01 | 0.1 | 0.86 | 0.14 | 68.23 |
| Bacteria | __Chlamydiae | __Chlamydiales | __cvE6 | __Chlamydiales | Other | 0.01 | 0.01 | 0.1 | 0.86 | 0.14 | 68.37 |
| Bacteria | __Proteobacteria | __Alphaproteobacteria | __Rhodospirillales | __wr0007 | Other | 0.01 | 0 | 0.1 | 0.42 | 0.14 | 68.51 |
| Bacteria | __Chlamydiae | __Chlamydiales | Other | Other | Other | 0.01 | 0.01 | 0.1 | 1.16 | 0.14 | 68.66 |
| Bacteria | __Acidobacteria | __Holophagae | __Acanthopleuribacterales | __Acanthopleuribacteraceae | __Acanthopleuribacter | 0 | 0.01 | 0.1 | 0.54 | 0.14 | 68.8 |
| Bacteria | __Firmicutes | __Bacilli | __Lactobacillales | __Enterococcaceae | __Enterococcus | 0 | 0.01 | 0.1 | 0.37 | 0.14 | 68.95 |
| Bacteria | __Proteobacteria | __Betaproteobacteria | __Burkholderiales | __Comamonadaceae | __Delftia | 0 | 0.01 | 0.1 | 0.78 | 0.14 | 69.09 |
| Bacteria | __Firmicutes | __Clostridia | __Clostridiales | __Family_XVIII_Incertae_Sedis | __Symbiobacterium | 0.02 | 0 | 0.1 | 0.56 | 0.14 | 69.23 |
| Bacteria | __Acidobacteria | __RB25 | __uncultured_bacterium | Other | Other | 0.01 | 0.01 | 0.1 | 0.63 | 0.14 | 69.37 |
| Bacteria | __Verrucomicrobia | __Candidatus_Methylacidiphilum | __uncultured_bacterium | Other | Other | 0 | 0.01 | 0.1 | 1.03 | 0.14 | 69.51 |
| Bacteria | __Proteobacteria | __Deltaproteobacteria | __Bdellovibrionales | __Bdellovibrionaceae | __OM27_clade | 0.01 | 0.01 | 0.1 | 0.5 | 0.14 | 69.65 |
| Bacteria | __Cyanobacteria | __SubsectionIII | __FamilyI | __Spirulina | __uncultured_bacterium | 0 | 0.01 | 0.1 | 0.42 | 0.14 | 69.79 |
| Bacteria | __Bacteroidetes | __Cytophagia | __Cytophagales | __Cytophagaceae | __Flexibacter | 0.02 | 0 | 0.1 | 0.3 | 0.14 | 69.93 |
| Bacteria | __Bacteroidetes | __Flavobacteria | __Flavobacteriales | __Flavobacteriaceae | __Cloacibacterium | 0 | 0.01 | 0.1 | 0.46 | 0.14 | 70.07 |
| Bacteria | __Firmicutes | __Bacilli | __Bacillales | __Planococcaceae | __Planococcus | 0.01 | 0.01 | 0.1 | 0.47 | 0.14 | 70.2 |
| Bacteria | __Proteobacteria | __Alphaproteobacteria | __DB1-14 | __uncultured_bacterium | Other | 0 | 0.01 | 0.1 | 0.86 | 0.14 | 70.34 |
| Bacteria | __Candidate_division_OD1 | __uncultured_bacterium | Other | Other | Other | 0.01 | 0.01 | 0.1 | 1.2 | 0.14 | 70.48 |
| Bacteria | __Proteobacteria | __Alphaproteobacteria | __Rhizobiales | __Family_Incertae_Sedis | __Bauldia | 0 | 0.01 | 0.1 | 0.97 | 0.14 | 70.61 |
| Bacteria | __Proteobacteria | __Alphaproteobacteria | __Rhodospirillales | __Candidatus_Alysiosphaera | __uncultured_alpha_proteobacterium | 0 | 0.01 | 0.1 | 1.14 | 0.14 | 70.75 |
| Bacteria | __Acidobacteria | __Candidatus_Chloracidobacterium | __uncultured_bacterium | Other | Other | 0.01 | 0.01 | 0.1 | 0.67 | 0.13 | 70.88 |
| Bacteria | __Actinobacteria | __Pseudonocardiales | __Pseudonocardiaceae | __Pseudonocardia | Other | 0.01 | 0.01 | 0.1 | 0.68 | 0.13 | 71.02 |
| Bacteria | __Cyanobacteria | __SubsectionIII | __FamilyI | __Leptolyngbya | Other | 0 | 0.01 | 0.1 | 0.81 | 0.13 | 71.15 |
| Bacteria | __Planctomycetes | __Phycisphaerae | __mle1-8 | __uncultured_bacterium | Other | 0 | 0.01 | 0.1 | 0.67 | 0.13 | 71.28 |
| Bacteria | __Bacteroidetes | __Flavobacteria | __Flavobacteriales | __Flavobacteriaceae | __Muricauda | 0 | 0.01 | 0.1 | 1.01 | 0.13 | 71.42 |
| Bacteria | __Acidobacteria | __DA023 | Other | Other | Other | 0.01 | 0 | 0.1 | 0.79 | 0.13 | 71.55 |
| Bacteria | __Proteobacteria | __Gammaproteobacteria | __KI89A_clade | __uncultured_bacterium | Other | 0 | 0.01 | 0.09 | 0.73 | 0.13 | 71.68 |
| Bacteria | __Cyanobacteria | __SubsectionII | __FamilyII | __Pleurocapsa | __uncultured_bacterium | 0 | 0.01 | 0.09 | 0.84 | 0.13 | 71.82 |
| Bacteria | __Firmicutes | __Clostridia | __Clostridiales | __Veillonellaceae | Other | 0.01 | 0 | 0.09 | 0.3 | 0.13 | 71.95 |
| Bacteria | __Firmicutes | __Bacilli | __Lactobacillales | __Carnobacteriaceae | __Granulicatella | 0.01 | 0 | 0.09 | 0.41 | 0.13 | 72.07 |
| Bacteria | __Proteobacteria | __Gammaproteobacteria | __Legionellales | __Legionellaceae | __Legionella | 0 | 0.01 | 0.09 | 0.87 | 0.13 | 72.2 |
| Bacteria | __Proteobacteria | __Alphaproteobacteria | __Rhodobacterales | __Rhodobacteraceae | __Roseovarius | 0 | 0.01 | 0.09 | 1.17 | 0.13 | 72.33 |
| Bacteria | __Proteobacteria | __Alphaproteobacteria | __Rhodospirillales | __Rhodospirillaceae | __Thalassospira | 0 | 0.01 | 0.09 | 0.38 | 0.13 | 72.46 |
| Bacteria | __Proteobacteria | __Alphaproteobacteria | __Rhizobiales | __Hyphomicrobiaceae | __Hyphomicrobium | 0.01 | 0.01 | 0.09 | 1 | 0.13 | 72.59 |
| Bacteria | __Acidobacteria | __Acidobacteriales | __Acidobacteriaceae | Other | Other | 0.01 | 0.01 | 0.09 | 0.41 | 0.13 | 72.72 |
| Bacteria | __Proteobacteria | __Deltaproteobacteria | __Desulfurellales | __Desulfurellaceae | __uncultured | 0.01 | 0 | 0.09 | 0.34 | 0.13 | 72.84 |
| Bacteria | __Proteobacteria | __Alphaproteobacteria | __Rickettsiales | __Rickettsiaceae | __Rickettsia | 0 | 0.01 | 0.09 | 0.65 | 0.13 | 72.97 |
| Bacteria | __Actinobacteria | __Corynebacteriales | __Corynebacteriaceae | __uncultured | Other | 0.01 | 0.01 | 0.09 | 0.74 | 0.13 | 73.1 |
| Bacteria | __Proteobacteria | __Alphaproteobacteria | __Rhizobiales | __Rhodobiaceae | Other | 0 | 0.01 | 0.09 | 0.93 | 0.13 | 73.22 |
| Bacteria | __Spirochaetes | __Spirochaetales | __Leptospiraceae | __uncultured | Other | 0 | 0.01 | 0.09 | 0.74 | 0.13 | 73.35 |
| Bacteria | __Proteobacteria | __Gammaproteobacteria | __Legionellales | __Coxiellaceae | __uncultured | 0.01 | 0.01 | 0.09 | 0.94 | 0.13 | 73.47 |
| Bacteria | __Cyanobacteria | __SubsectionII | __FamilyII | __Pleurocapsa | Other | 0 | 0.01 | 0.09 | 1.02 | 0.13 | 73.6 |
| Bacteria | __Proteobacteria | __Alphaproteobacteria | __Sphingomonadales | __Erythrobacteraceae | __Erythrobacter | 0 | 0.01 | 0.09 | 0.89 | 0.12 | 73.72 |
| Bacteria | __Acidobacteria | __Acidobacteriales | __Acidobacteriaceae | __uncultured | Other | 0.01 | 0 | 0.09 | 0.48 | 0.12 | 73.85 |
| Bacteria | __Firmicutes | __Bacilli | __Bacillales | __Alicyclobacillaceae | __Tumebacillus | 0 | 0.01 | 0.09 | 0.86 | 0.12 | 73.97 |
| Bacteria | __Bacteroidetes | __Flavobacteria | __Flavobacteriales | __Flavobacteriaceae | __Muricola | 0 | 0.01 | 0.09 | 0.78 | 0.12 | 74.09 |
| Bacteria | __Bacteroidetes | __Bacteroidia | __Bacteroidales | __Prevotellaceae | __Prevotella | 0.01 | 0 | 0.09 | 0.68 | 0.12 | 74.21 |
| Bacteria | __Proteobacteria | __Gammaproteobacteria | __EC3 | __uncultured_bacterium | Other | 0 | 0.01 | 0.09 | 0.97 | 0.12 | 74.33 |
| Bacteria | __Actinobacteria | __Thermoleophilia | __Gaiellales | __uncultured | Other | 0.01 | 0 | 0.09 | 0.65 | 0.12 | 74.46 |
| Bacteria | __Proteobacteria | __Gammaproteobacteria | __Oceanospirillales | __Oleiphilaceae | __Oleiphilus | 0 | 0.01 | 0.09 | 0.7 | 0.12 | 74.58 |
| Bacteria | __Planctomycetes | __Phycisphaerae | __Phycisphaerales | __Phycisphaeraceae | __SM1A02 | 0 | 0.01 | 0.09 | 0.71 | 0.12 | 74.7 |
| Bacteria | __Bacteroidetes | __Sphingobacteriia | __Sphingobacteriales | __Chitinophagaceae | __Sediminibacterium | 0 | 0.01 | 0.09 | 0.63 | 0.12 | 74.82 |
| Bacteria | __Actinobacteria | __Acidimicrobiia | __Acidimicrobiales | __Acidimicrobiaceae | __uncultured | 0 | 0.01 | 0.08 | 0.97 | 0.12 | 74.94 |
| Bacteria | __Proteobacteria | __Alphaproteobacteria | __Rhizobiales | __Phyllobacteriaceae | __Phyllobacterium | 0 | 0.01 | 0.08 | 1.29 | 0.12 | 75.06 |
| Bacteria | __Proteobacteria | __Alphaproteobacteria | __Rhodobacterales | __Rhodobacteraceae | __Wenxinia | 0 | 0.01 | 0.08 | 1.07 | 0.12 | 75.17 |
| Bacteria | __Proteobacteria | __Betaproteobacteria | __Burkholderiales | __Comamonadaceae | __uncultured | 0.01 | 0.01 | 0.08 | 0.93 | 0.12 | 75.29 |
| Bacteria | __Nitrospirae | __Nitrospira | __Nitrospirales | __0319-6A21 | __uncultured_bacterium | 0.01 | 0.01 | 0.08 | 0.67 | 0.12 | 75.41 |
| Bacteria | __Actinobacteria | __Micrococcales | __Micrococcaceae | __Kocuria | Other | 0 | 0.01 | 0.08 | 0.55 | 0.12 | 75.53 |
| Bacteria | __BD1-5 | __uncultured_bacterium | Other | Other | Other | 0 | 0.01 | 0.08 | 0.93 | 0.12 | 75.65 |
| Bacteria | __Proteobacteria | __Gammaproteobacteria | __Alteromonadales | __Alteromonadaceae | __BD1-7_clade | 0 | 0.01 | 0.08 | 0.78 | 0.12 | 75.77 |
| Bacteria | __Proteobacteria | __Gammaproteobacteria | __Enterobacteriales | __Enterobacteriaceae | __Salmonella | 0.01 | 0 | 0.08 | 0.93 | 0.12 | 75.88 |
| Bacteria | __Proteobacteria | __Alphaproteobacteria | __Rickettsiales | __Family_Incertae_Sedis | __Caedibacter | 0.01 | 0 | 0.08 | 0.35 | 0.12 | 76 |
| Bacteria | __Proteobacteria | __Gammaproteobacteria | __Enterobacteriales | __Enterobacteriaceae | Other | 0.01 | 0 | 0.08 | 0.52 | 0.12 | 76.11 |
| Bacteria | __Proteobacteria | __Betaproteobacteria | __Nitrosomonadales | __Nitrosomonadaceae | Other | 0.01 | 0 | 0.08 | 0.3 | 0.11 | 76.23 |
| Bacteria | __Proteobacteria | __Betaproteobacteria | __Burkholderiales | __Burkholderiaceae | __Ralstonia | 0.01 | 0 | 0.08 | 0.74 | 0.11 | 76.34 |
| Bacteria | __Chlamydiae | __Chlamydiales | __Parachlamydiaceae | __Candidatus_Protochlamydia | Other | 0 | 0.01 | 0.08 | 1.03 | 0.11 | 76.45 |
| Bacteria | __Bacteroidetes | __Bacteroidia | __Bacteroidales | __Porphyromonadaceae | __Paludibacter | 0 | 0.01 | 0.08 | 0.4 | 0.11 | 76.57 |
| Bacteria | __Actinobacteria | __Acidimicrobiia | __Acidimicrobiales | __uncultured | Other | 0 | 0.01 | 0.08 | 0.83 | 0.11 | 76.68 |
| Bacteria | __Bacteroidetes | __Cytophagia | __Cytophagales | __Cytophagaceae | __Siphonobacter | 0.01 | 0 | 0.08 | 0.33 | 0.11 | 76.79 |
| Bacteria | __Proteobacteria | __Deltaproteobacteria | __Sh765B-TzT-29 | Other | Other | 0 | 0.01 | 0.08 | 0.82 | 0.11 | 76.9 |
| Bacteria | __Planctomycetes | __Phycisphaerae | __Phycisphaerales | __Phycisphaeraceae | __Phycisphaera | 0 | 0.01 | 0.08 | 1.02 | 0.11 | 77.01 |
| Bacteria | __Proteobacteria | __Alphaproteobacteria | __Rhodospirillales | __Rhodospirillaceae | __Pelagibius | 0 | 0.01 | 0.08 | 0.66 | 0.11 | 77.12 |
| Bacteria | __Proteobacteria | __Deltaproteobacteria | __Bdellovibrionales | __Bacteriovoracaceae | __Peredibacter | 0 | 0.01 | 0.08 | 0.69 | 0.11 | 77.23 |
| Bacteria | __Actinobacteria | __Thermoleophilia | __Gaiellales | __Gaiellaceae | __Gaiella | 0.01 | 0 | 0.08 | 0.33 | 0.11 | 77.34 |
| Bacteria | __Actinobacteria | __Micrococcales | __Intrasporangiaceae | __Janibacter | Other | 0.01 | 0.01 | 0.08 | 0.48 | 0.11 | 77.45 |
| Bacteria | __Proteobacteria | __Gammaproteobacteria | __Thiotrichales | __H2-104-2 | __uncultured_deep-sea_bacterium | 0 | 0.01 | 0.08 | 0.56 | 0.11 | 77.56 |
| Bacteria | __Proteobacteria | __Alphaproteobacteria | __Rhodobacterales | __Rhodobacteraceae | __Rhodobacter | 0.01 | 0.01 | 0.08 | 1.13 | 0.11 | 77.67 |
| Bacteria | __Cyanobacteria | __SubsectionIII | __FamilyI | __Limnothrix | Other | 0 | 0.01 | 0.08 | 0.3 | 0.11 | 77.77 |
| Bacteria | __Bacteroidetes | __Cytophagia | __Cytophagales | __Cytophagaceae | __Hymenobacter | 0.01 | 0 | 0.08 | 0.34 | 0.11 | 77.88 |
| Bacteria | __Firmicutes | __Clostridia | __Clostridiales | __Ruminococcaceae | __uncultured | 0.01 | 0 | 0.08 | 0.43 | 0.11 | 77.98 |
| Bacteria | __Proteobacteria | __Alphaproteobacteria | __DB1-14 | Other | Other | 0.01 | 0 | 0.08 | 0.35 | 0.11 | 78.09 |
| Bacteria | __Proteobacteria | __Gammaproteobacteria | __Alteromonadales | __Pseudoalteromonadaceae | __Pseudoalteromonas | 0 | 0.01 | 0.07 | 0.55 | 0.11 | 78.19 |
| Bacteria | __Proteobacteria | __Alphaproteobacteria | __Rhizobiales | __Hyphomicrobiaceae | __Maritalea | 0.01 | 0.01 | 0.07 | 0.68 | 0.1 | 78.3 |
| Bacteria | __Bacteroidetes | __BSV13 | __uncultured_bacterium | Other | Other | 0.01 | 0 | 0.07 | 0.32 | 0.1 | 78.4 |
| Archaea | __Thaumarchaeota | __Soil_Crenarchaeotic_Group(SCG) | __uncultured_crenarchaeote | Other | Other | 0.01 | 0 | 0.07 | 0.34 | 0.1 | 78.51 |
| Bacteria | __Actinobacteria | Other | Other | Other | Other | 0.01 | 0.01 | 0.07 | 0.55 | 0.1 | 78.61 |
| Bacteria | __Verrucomicrobia | __Verrucomicrobiae | __Verrucomicrobiales | __Verrucomicrobiaceae | __Persicirhabdus | 0 | 0.01 | 0.07 | 0.63 | 0.1 | 78.71 |
| Bacteria | __Proteobacteria | __Deltaproteobacteria | __Myxococcales | __Sorangiineae | __uncultured | 0.01 | 0 | 0.07 | 0.38 | 0.1 | 78.81 |
| Bacteria | __Planctomycetes | __OM190 | Other | Other | Other | 0 | 0.01 | 0.07 | 0.75 | 0.1 | 78.91 |
| Bacteria | __Bacteroidetes | __SB-1 | __uncultured_bacterium | Other | Other | 0 | 0.01 | 0.07 | 0.86 | 0.1 | 79.01 |
| Bacteria | __Deinococcus-Thermus | __Deinococci | __Thermales | __Thermaceae | __Meiothermus | 0.01 | 0 | 0.07 | 0.4 | 0.1 | 79.11 |
| Bacteria | __Actinobacteria | __Micromonosporales | __Micromonosporaceae | __Actinoplanes | Other | 0.01 | 0 | 0.07 | 0.44 | 0.1 | 79.21 |
| Bacteria | __Verrucomicrobia | __Spartobacteria | __Chthoniobacterales | __DA101_soil_group | Other | 0.01 | 0 | 0.07 | 0.82 | 0.1 | 79.31 |
| Bacteria | __Actinobacteria | __Bifidobacteriales | __Bifidobacteriaceae | __Bifidobacterium | Other | 0 | 0.01 | 0.07 | 0.48 | 0.1 | 79.41 |
| Bacteria | __Proteobacteria | __Gammaproteobacteria | __Alteromonadales | __Alteromonadaceae | __Microbulbifer | 0 | 0.01 | 0.07 | 0.76 | 0.1 | 79.5 |
| Bacteria | __Proteobacteria | __Deltaproteobacteria | __Myxococcales | __0319-6G20 | __uncultured_delta_proteobacterium | 0.01 | 0 | 0.07 | 0.36 | 0.1 | 79.6 |
| Bacteria | __Proteobacteria | __Alphaproteobacteria | __Rhizobiales | __Hyphomicrobiaceae | __Devosia | 0 | 0.01 | 0.07 | 0.99 | 0.1 | 79.7 |
| Bacteria | __Bacteroidetes | __Flavobacteria | __Flavobacteriales | __Flavobacteriaceae | __Croceitalea | 0 | 0.01 | 0.07 | 0.85 | 0.1 | 79.79 |
| Bacteria | __Acidobacteria | __DA023 | __uncultured_soil_bacterium | Other | Other | 0.01 | 0 | 0.07 | 0.3 | 0.1 | 79.89 |
| Bacteria | __Acidobacteria | __Candidatus_Solibacter | Other | Other | Other | 0.01 | 0 | 0.07 | 0.3 | 0.1 | 79.98 |
| Bacteria | __Verrucomicrobia | __Verrucomicrobiae | __Verrucomicrobiales | __Rubritaleaceae | __Rubritalea | 0 | 0.01 | 0.07 | 0.75 | 0.09 | 80.08 |
| Bacteria | __Chloroflexi | __Ktedonobacteria | __Ktedonobacterales | __Ktedonobacteraceae | __uncultured | 0.01 | 0 | 0.07 | 0.3 | 0.09 | 80.17 |
| Bacteria | __Acidobacteria | __RB41 | Other | Other | Other | 0.01 | 0 | 0.07 | 0.37 | 0.09 | 80.26 |
| Bacteria | __Proteobacteria | __Gammaproteobacteria | __Chromatiales | __Chromatiaceae | __Marichromatium | 0.01 | 0 | 0.07 | 0.57 | 0.09 | 80.36 |
| Bacteria | __Proteobacteria | __Alphaproteobacteria | __Rhodobacterales | __Rhodobacteraceae | __Roseobacter | 0.01 | 0 | 0.07 | 0.49 | 0.09 | 80.45 |
| Bacteria | __Proteobacteria | __Alphaproteobacteria | __Rhodobacterales | __Rhodobacteraceae | __Roseivivax | 0 | 0.01 | 0.07 | 0.78 | 0.09 | 80.54 |
| Bacteria | __Cyanobacteria | __SubsectionIII | __FamilyI | Other | Other | 0 | 0.01 | 0.07 | 0.68 | 0.09 | 80.64 |
| Bacteria | __Proteobacteria | __Gammaproteobacteria | __Alteromonadales | __Alteromonadaceae | __Melitea | 0 | 0.01 | 0.07 | 0.66 | 0.09 | 80.73 |
| Bacteria | __Acidobacteria | __BPC015 | Other | Other | Other | 0 | 0.01 | 0.07 | 0.51 | 0.09 | 80.82 |
| Bacteria | __Proteobacteria | __Alphaproteobacteria | __Rhizobiales | __Hyphomicrobiaceae | __Pedomicrobium | 0.01 | 0 | 0.07 | 0.3 | 0.09 | 80.91 |
| Bacteria | __Verrucomicrobia | __OPB35_soil_group | __uncultured_Verrucomicrobia_subdivision_3_bacterium | Other | Other | 0.01 | 0 | 0.07 | 0.34 | 0.09 | 81 |
| Bacteria | __Actinobacteria | __Thermoleophilia | __Solirubrobacterales | __TM146 | __uncultured_bacterium | 0.01 | 0 | 0.07 | 0.32 | 0.09 | 81.1 |
| Bacteria | __Proteobacteria | __Gammaproteobacteria | __Pseudomonadales | __Moraxellaceae | __Psychrobacter | 0 | 0.01 | 0.07 | 0.41 | 0.09 | 81.19 |
| Bacteria | __Proteobacteria | __Alphaproteobacteria | __Kordiimonadales | __Kordiimonadaceae | __Kordiimonas | 0 | 0.01 | 0.07 | 0.32 | 0.09 | 81.28 |
| Bacteria | __Firmicutes | __Bacilli | __Bacillales | __Staphylococcaceae | __Salinicoccus | 0.01 | 0 | 0.06 | 0.44 | 0.09 | 81.37 |
| Bacteria | __Proteobacteria | __Alphaproteobacteria | __OCS116_clade | __uncultured_bacterium | Other | 0 | 0.01 | 0.06 | 0.78 | 0.09 | 81.46 |
| Bacteria | __Cyanobacteria | __uncultured | __uncultured_bacterium | Other | Other | 0.01 | 0 | 0.06 | 0.36 | 0.09 | 81.55 |
| Bacteria | __Proteobacteria | __Gammaproteobacteria | __Alteromonadales | __Alteromonadaceae | __OM60(NOR5)_clade | 0 | 0.01 | 0.06 | 0.73 | 0.09 | 81.64 |
| Bacteria | __Proteobacteria | __Gammaproteobacteria | __Thiotrichales | __EV818SWSAP88 | Other | 0 | 0.01 | 0.06 | 0.84 | 0.09 | 81.73 |
| Bacteria | __Firmicutes | __Clostridia | __Clostridiales | __Veillonellaceae | __Megasphaera | 0.01 | 0 | 0.06 | 0.33 | 0.09 | 81.82 |
| Bacteria | __Bacteroidetes | __Sphingobacteriia | __Sphingobacteriales | __AKYH767 | __uncultured_bacterium | 0.01 | 0 | 0.06 | 0.3 | 0.09 | 81.91 |
| Bacteria | __Bacteroidetes | __Flavobacteria | __Flavobacteriales | __Flavobacteriaceae | __Tenacibaculum | 0 | 0.01 | 0.06 | 0.56 | 0.09 | 82 |
| Bacteria | __Proteobacteria | __Alphaproteobacteria | __Rickettsiales | __Holosporaceae | __Holospora | 0.01 | 0 | 0.06 | 0.45 | 0.09 | 82.09 |
| Bacteria | __Firmicutes | __Bacilli | __Bacillales | __Paenibacillaceae | __Paenibacillus | 0.01 | 0 | 0.06 | 0.43 | 0.09 | 82.18 |
| Bacteria | __Firmicutes | __Bacilli | __Bacillales | __Listeriaceae | __Brochothrix | 0.01 | 0 | 0.06 | 0.3 | 0.09 | 82.26 |
| Bacteria | __Proteobacteria | __Deltaproteobacteria | __Bdellovibrionales | __Bacteriovoracaceae | Other | 0 | 0.01 | 0.06 | 0.73 | 0.09 | 82.35 |
| Bacteria | __Firmicutes | __Bacilli | __Lactobacillales | __Streptococcaceae | __Lactococcus | 0.01 | 0 | 0.06 | 0.4 | 0.09 | 82.44 |
| Bacteria | __Proteobacteria | __Gammaproteobacteria | __Oceanospirillales | __Oceanospirillaceae | __Marinobacterium | 0 | 0.01 | 0.06 | 0.75 | 0.09 | 82.52 |
| Bacteria | __Proteobacteria | __Gammaproteobacteria | __Xanthomonadales | __Xanthomonadaceae | __Pseudoxanthomonas | 0 | 0.01 | 0.06 | 0.75 | 0.09 | 82.61 |
| Bacteria | __Actinobacteria | __Streptosporangiales | __Thermomonosporaceae | __Actinoallomurus | Other | 0.01 | 0 | 0.06 | 0.38 | 0.09 | 82.7 |
| Bacteria | __Proteobacteria | __Alphaproteobacteria | __DB1-14 | __uncultured_alpha_proteobacterium | Other | 0 | 0.01 | 0.06 | 0.6 | 0.09 | 82.78 |
| Bacteria | __Chloroflexi | __Ktedonobacteria | __Ktedonobacterales | __HSB_OF53-F07 | __uncultured_bacterium | 0.01 | 0 | 0.06 | 0.38 | 0.08 | 82.87 |
| Bacteria | __Firmicutes | __Clostridia | __Clostridiales | __Clostridiaceae | __Caminicella | 0.01 | 0 | 0.06 | 1.02 | 0.08 | 82.95 |
| Bacteria | __Cyanobacteria | __SubsectionI | __FamilyI | __uncultured_cyanobacterium | Other | 0 | 0.01 | 0.06 | 0.67 | 0.08 | 83.03 |
| Bacteria | __Proteobacteria | __Alphaproteobacteria | __Rhodospirillales | __Rhodospirillaceae | Other | 0 | 0.01 | 0.06 | 0.69 | 0.08 | 83.12 |
| Bacteria | __Bacteroidetes | __Sphingobacteriia | __Sphingobacteriales | __Chitinophagaceae | __Hydrotalea | 0 | 0.01 | 0.06 | 0.6 | 0.08 | 83.2 |
| Bacteria | __Spirochaetes | __Spirochaetales | __Leptospiraceae | __Turneriella | __uncultured_bacterium | 0 | 0.01 | 0.06 | 0.74 | 0.08 | 83.28 |
| Bacteria | __Actinobacteria | __Micrococcales | Other | Other | Other | 0.01 | 0 | 0.06 | 0.42 | 0.08 | 83.36 |
| Bacteria | __Proteobacteria | __Betaproteobacteria | __B1-7BS | __uncultured_bacterium | Other | 0.01 | 0 | 0.06 | 0.36 | 0.08 | 83.44 |
| Bacteria | __Bacteroidetes | __Sphingobacteriia | __Sphingobacteriales | __Chitinophagaceae | __Chitinophaga | 0 | 0.01 | 0.06 | 0.3 | 0.08 | 83.53 |
| Bacteria | __Proteobacteria | __Gammaproteobacteria | __E01-9C-26_marine_group | Other | Other | 0 | 0.01 | 0.06 | 0.68 | 0.08 | 83.6 |
| Bacteria | __Proteobacteria | __Alphaproteobacteria | __Rhodobacterales | __Rhodobacteraceae | __Loktanella | 0 | 0.01 | 0.06 | 0.84 | 0.08 | 83.68 |
| Bacteria | __Proteobacteria | __Gammaproteobacteria | __Xanthomonadales | __Xanthomonadaceae | Other | 0 | 0.01 | 0.06 | 0.7 | 0.08 | 83.76 |
| Bacteria | __Proteobacteria | __Alphaproteobacteria | __Rhizobiales | __Rhizobiaceae | __Rhizobium | 0 | 0.01 | 0.06 | 0.61 | 0.08 | 83.84 |
| Bacteria | __Actinobacteria | __Acidimicrobiia | __Acidimicrobiales | __uncultured | __uncultured_actinobacterium | 0 | 0.01 | 0.06 | 0.77 | 0.08 | 83.92 |
| Bacteria | __Firmicutes | __Clostridia | __Clostridiales | __Lachnospiraceae | Other | 0.01 | 0 | 0.06 | 0.55 | 0.08 | 84 |
| Bacteria | __Proteobacteria | __Gammaproteobacteria | __Alteromonadales | __Alteromonadaceae | __Marinobacter | 0 | 0.01 | 0.06 | 0.65 | 0.08 | 84.08 |
| Bacteria | __Proteobacteria | __Gammaproteobacteria | __Xanthomonadales | __Xanthomonadaceae | __Stenotrophomonas | 0.01 | 0 | 0.06 | 0.43 | 0.08 | 84.15 |
| Bacteria | __Proteobacteria | __Betaproteobacteria | __Methylophilales | __Methylophilaceae | __OM43_clade | 0.01 | 0 | 0.05 | 0.82 | 0.08 | 84.23 |
| Bacteria | __Cyanobacteria | __SubsectionIII | __FamilyI | __uncultured | __uncultured_bacterium | 0 | 0.01 | 0.05 | 0.52 | 0.08 | 84.31 |
| Bacteria | __Acidobacteria | __Holophagae | __Sva0725 | __uncultured_bacterium | Other | 0.01 | 0 | 0.05 | 0.63 | 0.08 | 84.38 |
| Bacteria | __Proteobacteria | __Betaproteobacteria | __Burkholderiales | __Burkholderiaceae | __Limnobacter | 0 | 0.01 | 0.05 | 0.7 | 0.08 | 84.46 |
| Bacteria | __Planctomycetes | __Planctomycetacia | __Brocadiales | __Brocadiaceae | __Candidatus_Brocadia | 0 | 0.01 | 0.05 | 0.65 | 0.08 | 84.54 |
| Bacteria | __Proteobacteria | __Alphaproteobacteria | __E6aD10 | Other | Other | 0 | 0 | 0.05 | 0.78 | 0.08 | 84.61 |
| Bacteria | __Firmicutes | __Bacilli | __Bacillales | __Bacillaceae | Other | 0 | 0.01 | 0.05 | 0.3 | 0.08 | 84.69 |
| Bacteria | __Proteobacteria | __Alphaproteobacteria | __Rhizobiales | __Phyllobacteriaceae | __Mesorhizobium | 0.01 | 0 | 0.05 | 0.45 | 0.08 | 84.77 |
| Bacteria | __Verrucomicrobia | __Candidatus_Methylacidiphilum | __uncultured_Verrucomicrobia_bacterium | Other | Other | 0 | 0.01 | 0.05 | 0.39 | 0.08 | 84.84 |
| Bacteria | __Acidobacteria | __32-21 | __uncultured_Acidobacteria_bacterium | Other | Other | 0.01 | 0 | 0.05 | 0.39 | 0.08 | 84.92 |
| Bacteria | __Proteobacteria | __Alphaproteobacteria | __Caulobacterales | __Hyphomonadaceae | __Hyphomonas | 0 | 0.01 | 0.05 | 0.78 | 0.07 | 84.99 |
| Bacteria | __Proteobacteria | __Deltaproteobacteria | __Syntrophobacterales | __Syntrophaceae | __Syntrophus | 0 | 0.01 | 0.05 | 0.76 | 0.07 | 85.07 |
| Bacteria | __Acidobacteria | __Acidobacteriales | __Acidobacteriaceae | __Acidobacterium | __uncultured_bacterium | 0.01 | 0 | 0.05 | 0.3 | 0.07 | 85.14 |
| Bacteria | __Acidobacteria | __Holophagae | __Sva0725 | Other | Other | 0 | 0.01 | 0.05 | 0.57 | 0.07 | 85.21 |
| Bacteria | __Planctomycetes | __Phycisphaerae | __Phycisphaerales | __Phycisphaeraceae | __Urania-1B-19_marine_sediment_group | 0 | 0.01 | 0.05 | 0.73 | 0.07 | 85.28 |
| Bacteria | __Gemmatimonadetes | __BD2-11_terrestrial_group | __uncultured_bacterium | Other | Other | 0 | 0.01 | 0.05 | 0.56 | 0.07 | 85.35 |
| Bacteria | __Proteobacteria | __Alphaproteobacteria | __Rhodospirillales | __DA111 | Other | 0 | 0 | 0.05 | 0.42 | 0.07 | 85.43 |
| Bacteria | __Verrucomicrobia | __Verrucomicrobiae | __Verrucomicrobiales | __Verrucomicrobiaceae | __Roseibacillus | 0 | 0.01 | 0.05 | 0.66 | 0.07 | 85.5 |
| Bacteria | __Proteobacteria | __Gammaproteobacteria | __Enterobacteriales | __Enterobacteriaceae | __Proteus | 0 | 0.01 | 0.05 | 0.52 | 0.07 | 85.57 |
| Bacteria | __Proteobacteria | __Gammaproteobacteria | __Thiotrichales | __Family_Incertae_Sedis | __Caedibacter | 0 | 0.01 | 0.05 | 0.63 | 0.07 | 85.64 |
| Bacteria | __Proteobacteria | __Gammaproteobacteria | __Thiotrichales | __EV818SWSAP88 | __uncultured_bacterium | 0 | 0.01 | 0.05 | 0.53 | 0.07 | 85.71 |
| Bacteria | __Bacteroidetes | __WCHB1-32 | __uncultured_bacterium | Other | Other | 0 | 0.01 | 0.05 | 1.03 | 0.07 | 85.78 |
| Bacteria | __Bacteroidetes | __Sphingobacteriia | __Sphingobacteriales | __Sphingobacteriaceae | Other | 0.01 | 0 | 0.05 | 0.33 | 0.07 | 85.84 |
| Bacteria | __Proteobacteria | __Alphaproteobacteria | __Sphingomonadales | __Erythrobacteraceae | __uncultured | 0 | 0.01 | 0.05 | 0.33 | 0.07 | 85.91 |
| Bacteria | __Bacteroidetes | Other | Other | Other | Other | 0 | 0.01 | 0.05 | 0.62 | 0.07 | 85.98 |
| Bacteria | __Proteobacteria | __Gammaproteobacteria | __Chromatiales | __Chromatiaceae | __Nitrosococcus | 0 | 0.01 | 0.05 | 0.41 | 0.07 | 86.05 |
| Bacteria | __Proteobacteria | __Alphaproteobacteria | __Rickettsiales | Other | Other | 0 | 0.01 | 0.05 | 0.57 | 0.07 | 86.12 |
| Bacteria | __Firmicutes | __Clostridia | __Clostridiales | __Peptostreptococcaceae | __Tepidibacter | 0.01 | 0 | 0.05 | 0.47 | 0.07 | 86.18 |
| Bacteria | __Verrucomicrobia | __Verrucomicrobiae | __Verrucomicrobiales | __P._palm_C_85 | Other | 0 | 0.01 | 0.05 | 0.36 | 0.07 | 86.25 |
| Bacteria | __Proteobacteria | __Deltaproteobacteria | __Myxococcales | __Nannocystineae | __Nannocystaceae | 0 | 0.01 | 0.05 | 0.74 | 0.07 | 86.32 |
| Bacteria | __Verrucomicrobia | __Verrucomicrobiae | __Verrucomicrobiales | __DEV007 | Other | 0 | 0.01 | 0.05 | 0.77 | 0.07 | 86.38 |
| Bacteria | __Acidobacteria | __Holophagae | __CA002 | __uncultured_bacterium | Other | 0 | 0.01 | 0.05 | 0.53 | 0.07 | 86.45 |
| Bacteria | __Cyanobacteria | __SubsectionIV | __FamilyII | __Rivularia | __Nostocales | 0 | 0.01 | 0.05 | 0.75 | 0.07 | 86.51 |
| Bacteria | __Acidobacteria | __Acidobacteriales | __Acidobacteriaceae | __Candidatus_Koribacter | __uncultured_bacterium | 0 | 0.01 | 0.05 | 0.35 | 0.07 | 86.58 |
| Bacteria | __Proteobacteria | __Gammaproteobacteria | __Alteromonadales | __Alteromonadaceae | __BD2-7 | 0 | 0.01 | 0.05 | 0.3 | 0.06 | 86.64 |
| Bacteria | __Proteobacteria | __Gammaproteobacteria | __Xanthomonadales | __Xanthomonadaceae | __Dyella | 0.01 | 0 | 0.05 | 0.3 | 0.06 | 86.71 |
| Bacteria | __Firmicutes | __Clostridia | __Clostridiales | __Clostridiaceae | __Oxobacter | 0 | 0 | 0.05 | 0.49 | 0.06 | 86.77 |
| Bacteria | __Chlamydiae | __Chlamydiales | __Chlamydiaceae | __uncultured_Chlamydiales_bacterium | Other | 0 | 0 | 0.05 | 0.52 | 0.06 | 86.83 |
| Bacteria | __Acidobacteria | __RB41 | __uncultured_Acidobacteria_bacterium | Other | Other | 0.01 | 0 | 0.05 | 0.3 | 0.06 | 86.9 |
| Bacteria | __Acidobacteria | __Holophagae | __TK85 | Other | Other | 0 | 0.01 | 0.05 | 0.89 | 0.06 | 86.96 |
| Bacteria | __Proteobacteria | __Alphaproteobacteria | __Rhizobiales | __KF-JG30-B3 | __uncultured_bacterium | 0.01 | 0 | 0.05 | 0.35 | 0.06 | 87.03 |
| Bacteria | __Firmicutes | __Bacilli | __Bacillales | __Family_XI_Incertae_Sedis | __Gemella | 0 | 0.01 | 0.05 | 0.68 | 0.06 | 87.09 |
| Bacteria | __Proteobacteria | __Gammaproteobacteria | __Alteromonadales | __Alteromonadaceae | __uncultured | 0 | 0.01 | 0.04 | 0.35 | 0.06 | 87.15 |
| Bacteria | __Proteobacteria | __Betaproteobacteria | __Burkholderiales | __Comamonadaceae | __Pseudorhodoferax | 0.01 | 0 | 0.04 | 0.51 | 0.06 | 87.21 |
| Bacteria | __Proteobacteria | __Alphaproteobacteria | __Sphingomonadales | __Erythrobacteraceae | __Altererythrobacter | 0 | 0.01 | 0.04 | 0.54 | 0.06 | 87.28 |
| Bacteria | __Bacteroidetes | __Sphingobacteriia | __Sphingobacteriales | __Sphingobacteriaceae | __Pedobacter | 0.01 | 0 | 0.04 | 0.41 | 0.06 | 87.34 |
| Bacteria | __Actinobacteria | __Corynebacteriales | __Corynebacteriaceae | __Corynebacterium | __Corynebacterium_freneyi | 0.01 | 0 | 0.04 | 0.3 | 0.06 | 87.4 |
| Bacteria | __Bacteroidetes | __Cytophagia | __Cytophagales | __Flammeovirgaceae | __Candidatus_Amoebophilus | 0 | 0.01 | 0.04 | 0.66 | 0.06 | 87.46 |
| Bacteria | __Actinobacteria | __Acidimicrobiia | __Acidimicrobiales | __TM214 | __uncultured_bacterium | 0 | 0 | 0.04 | 0.39 | 0.06 | 87.52 |
| Bacteria | __Proteobacteria | __Alphaproteobacteria | __Rhodospirillales | __Rhodospirillaceae | __Azospirillum | 0 | 0.01 | 0.04 | 0.64 | 0.06 | 87.59 |
| Bacteria | __Planctomycetes | __OM190 | __uncultured_planctomycete | Other | Other | 0 | 0.01 | 0.04 | 0.69 | 0.06 | 87.65 |
| Bacteria | __Proteobacteria | __Betaproteobacteria | __Rhodocyclales | __Rhodocyclaceae | __uncultured | 0 | 0 | 0.04 | 0.42 | 0.06 | 87.71 |
| Bacteria | __Proteobacteria | __Deltaproteobacteria | __Myxococcales | Other | Other | 0 | 0.01 | 0.04 | 0.68 | 0.06 | 87.77 |
| Bacteria | __Proteobacteria | __Betaproteobacteria | __Neisseriales | __Neisseriaceae | __uncultured | 0 | 0 | 0.04 | 0.48 | 0.06 | 87.83 |
| Bacteria | __Armatimonadetes | __Chthonomonadetes | __Chthonomonadales | __uncultured_bacterium | Other | 0 | 0.01 | 0.04 | 0.39 | 0.06 | 87.89 |
| Bacteria | __Proteobacteria | __Deltaproteobacteria | __Desulfobacterales | __Desulfobulbaceae | __SEEP-SRB4 | 0 | 0.01 | 0.04 | 0.51 | 0.06 | 87.95 |
| Bacteria | __Proteobacteria | __Alphaproteobacteria | __Caulobacterales | __Hyphomonadaceae | __Oceanicaulis | 0 | 0.01 | 0.04 | 0.3 | 0.06 | 88.01 |
| Bacteria | __Proteobacteria | __Gammaproteobacteria | __Thiotrichales | __H2-104-2 | __uncultured_bacterium | 0 | 0.01 | 0.04 | 0.66 | 0.06 | 88.07 |
| Bacteria | __Proteobacteria | __Betaproteobacteria | __Rhodocyclales | __Rhodocyclaceae | __Ferribacterium | 0.01 | 0 | 0.04 | 0.79 | 0.06 | 88.13 |
| Bacteria | __Proteobacteria | __Skagenf62 | __uncultured_bacterium | Other | Other | 0 | 0.01 | 0.04 | 0.71 | 0.06 | 88.19 |
| Bacteria | __Gemmatimonadetes | __Gemmatimonadales | __Gemmatimonadaceae | __uncultured | __uncultured_bacterium | 0.01 | 0 | 0.04 | 0.45 | 0.06 | 88.25 |
| Bacteria | __Proteobacteria | __Deltaproteobacteria | __Desulfuromonadales | __GR-WP33-58 | Other | 0 | 0.01 | 0.04 | 0.51 | 0.06 | 88.31 |
| Bacteria | __Actinobacteria | __Micrococcales | __Dermabacteraceae | __Brachybacterium | Other | 0.01 | 0 | 0.04 | 0.44 | 0.06 | 88.37 |
| Bacteria | __Armatimonadetes | __Chthonomonadetes | __Chthonomonadales | __Chthonomonadaceae | __Chthonomonas | 0.01 | 0 | 0.04 | 0.33 | 0.06 | 88.43 |
| Bacteria | __Actinobacteria | __Propionibacteriales | __Propionibacteriaceae | __Propionibacterium | Other | 0.01 | 0 | 0.04 | 0.68 | 0.06 | 88.49 |
| Bacteria | __Firmicutes | __Clostridia | __Clostridiales | __Veillonellaceae | __Veillonella | 0 | 0 | 0.04 | 0.53 | 0.06 | 88.55 |
| Bacteria | __Proteobacteria | __Gammaproteobacteria | __NKB5 | Other | Other | 0 | 0.01 | 0.04 | 0.7 | 0.06 | 88.61 |
| Bacteria | __Proteobacteria | __SC3-20 | __uncultured_gamma_proteobacterium | Other | Other | 0 | 0.01 | 0.04 | 0.83 | 0.06 | 88.66 |
| Bacteria | __Chloroflexi | __TK10 | Other | Other | Other | 0 | 0.01 | 0.04 | 0.3 | 0.06 | 88.72 |
| Bacteria | __Chloroflexi | __Caldilineae | __Caldilineales | __Caldilineaceae | __Caldilinea | 0 | 0.01 | 0.04 | 0.66 | 0.06 | 88.78 |
| Bacteria | __Firmicutes | __Clostridia | __Clostridiales | __Christensenellaceae | __uncultured | 0 | 0 | 0.04 | 0.5 | 0.06 | 88.84 |
| Bacteria | __Actinobacteria | __Frankiales | __Acidothermaceae | __Acidothermus | __uncultured_actinobacterium | 0.01 | 0 | 0.04 | 0.39 | 0.06 | 88.89 |
| Bacteria | __Bacteroidetes | __Sphingobacteriia | __Sphingobacteriales | __NS11-12_marine_group | Other | 0 | 0.01 | 0.04 | 0.74 | 0.06 | 88.95 |
| Bacteria | __Proteobacteria | __Alphaproteobacteria | __Rickettsiales | __Candidatus_Hepatincola | __uncultured_bacterium | 0 | 0 | 0.04 | 0.58 | 0.06 | 89.01 |
| Bacteria | __Gemmatimonadetes | __Gemmatimonadales | __Gemmatimonadaceae | __Gemmatimonas | Other | 0 | 0 | 0.04 | 0.41 | 0.06 | 89.06 |
| Bacteria | __Proteobacteria | __TA18 | Other | Other | Other | 0 | 0.01 | 0.04 | 0.56 | 0.06 | 89.12 |
| Bacteria | __Proteobacteria | __Alphaproteobacteria | __Rickettsiales | __Candidatus_Odyssella | __uncultured_bacterium | 0.01 | 0 | 0.04 | 0.3 | 0.06 | 89.18 |
| Bacteria | __Proteobacteria | __Alphaproteobacteria | __SB1-18 | __uncultured_bacterium | Other | 0 | 0 | 0.04 | 0.58 | 0.06 | 89.23 |
| Bacteria | __Cyanobacteria | __SubsectionIII | __FamilyI | __Planktothrix | Other | 0 | 0.01 | 0.04 | 0.52 | 0.06 | 89.29 |
| Bacteria | __Proteobacteria | __Alphaproteobacteria | __Rhodobacterales | __Rhodobacteraceae | __Dinoroseobacter | 0 | 0 | 0.04 | 0.65 | 0.06 | 89.34 |
| Bacteria | __Proteobacteria | __Candidatus_Allobeggiatoa | Other | Other | Other | 0 | 0.01 | 0.04 | 0.66 | 0.06 | 89.4 |
| Bacteria | __Proteobacteria | __Deltaproteobacteria | __Desulfobacterales | __Desulfobulbaceae | __Desulforhopalus | 0 | 0 | 0.04 | 0.83 | 0.06 | 89.46 |
| Bacteria | __Proteobacteria | __Alphaproteobacteria | __Rickettsiales | __Candidatus_Odyssella | __uncultured_alpha_proteobacterium | 0 | 0 | 0.04 | 0.3 | 0.06 | 89.51 |
| Bacteria | __Proteobacteria | __Deltaproteobacteria | __Desulfuromonadales | __Geobacteraceae | __Geobacter | 0 | 0 | 0.04 | 0.39 | 0.05 | 89.57 |
| Bacteria | __Firmicutes | __Erysipelotrichi | __Erysipelotrichales | __Erysipelotrichaceae | __Turicibacter | 0 | 0 | 0.04 | 0.47 | 0.05 | 89.62 |
| Bacteria | __Proteobacteria | __Gammaproteobacteria | __Oceanospirillales | __Oceanospirillaceae | __Pseudospirillum | 0 | 0.01 | 0.04 | 0.79 | 0.05 | 89.67 |
| Bacteria | __Bacteroidetes | __Flavobacteria | __Flavobacteriales | __Cryomorphaceae | __Crocinitomix | 0 | 0 | 0.04 | 0.48 | 0.05 | 89.73 |
| Bacteria | __Acidobacteria | __Holophagae | __CA002 | Other | Other | 0 | 0.01 | 0.04 | 0.54 | 0.05 | 89.78 |
| Bacteria | __Proteobacteria | __Deltaproteobacteria | __Desulfobacterales | __Desulfobulbaceae | __Desulfocapsa | 0 | 0 | 0.04 | 0.71 | 0.05 | 89.83 |
| Bacteria | __Proteobacteria | __Alphaproteobacteria | __Rickettsiales | __Candidatus_Odyssella | Other | 0 | 0.01 | 0.04 | 0.54 | 0.05 | 89.89 |
| Bacteria | __Actinobacteria | __Micrococcales | __Dermacoccaceae | Other | Other | 0.01 | 0 | 0.04 | 0.4 | 0.05 | 89.94 |
| Bacteria | __Verrucomicrobia | __OPB35_soil_group | __uncultured_Verrucomicrobia_bacterium | Other | Other | 0 | 0 | 0.04 | 0.42 | 0.05 | 89.99 |
| Bacteria | __Firmicutes | __Clostridia | __Clostridiales | __Family_XIII_Incertae_Sedis | __uncultured | 0 | 0 | 0.04 | 0.6 | 0.05 | 90.05 |
