# Supplementary material for: Coral physiology and microbiome dynamics under combined warming and ocean acidification
Source: PLoS One. 2018 Jan 16;13(1):e0191156. doi: 10.1371/journal.pone.0191156 (PMC5770069; doi:10.1371/journal.pone.0191156)
Supplement: S3 Table — Effects of species and treatment were fixed and fully crossed. A posteriori slice tests were used to test for differences between treatment and controls within species. df = degrees of freedom, SS = type III sum of squares of the main effects, F = F-statistic. Significant effects are bolded. (DOCX) [file pone.0191156.s005.docx]

**Supporting Information**

**Coral physiology and microbiome dynamics under combined warming and ocean acidification**

Andréa G Grottoli, Paula Dalcin Martins, Michael J. Wilkins, Michael D. Johnston, Mark E Warner, Wei-Jun Cai, Todd F. Melman, Kenneth D. Hoadley, D. Tye Pettay, Stephen Levas, Verena Schoepf

**S3 Table. Results of a two-way ANOVA testing the effect of species and treatment on Shannon Diversity Index.**

| **ANOVA Effect** | **df** | **SS** | **F** | **p-value** |
| --- | --- | --- | --- | --- |
| Model | 3 | 7.2057 | 3.82 | **0.026** |
| Species | 1 | 1.8732 | 2.89 | 0.099 |
| Treatment | 1 | 4.5423 | 7.22 | **0.014** |
| Species x Treatment | 1 | 0.7913 | 1.26 | 0.276 |
|  |  |  |  |  |
| **Slice Test** |  |  |  |  |
| *A. millepora* | 1 | 4.5609 | 7.24 | **0.014** |
| *T. reniformis* | 1 | 0.7716 | 1.23 | 0.281 |
|  |  |  |  |  |

Effects of species and treatment were fixed and fully crossed. *A posteriori* slice tests were used to test for differences between treatment and controls within species. df = degrees of freedom, SS = type III sum of squares of the main effects, F = F-statistic. Significant effects are bolded.
