# Supplementary material for: Coral physiology and microbiome dynamics under combined warming and ocean acidification
Source: PLoS One. 2018 Jan 16;13(1):e0191156. doi: 10.1371/journal.pone.0191156 (PMC5770069; doi:10.1371/journal.pone.0191156)
Supplement: S4 Table — Significant differences (p ≤ 0.05) are bolded. calc = calcification, chla = chlorophyll a, cells = endosymbiotic algal cell density, lipid = total lipids, protein = soluble animal protein concentration, carbs = carbohydrate concentration, biomass = coral ash free dry weight per area, h = host, a = endosymbiotic algae. (DOCX) [file pone.0191156.s006.docx]

**Supporting Information**

**Coral physiology and microbiome dynamics under combined warming and ocean acidification**

Andréa G Grottoli, Paula Dalcin Martins, Michael J. Wilkins, Michael D. Johnston, Mark E Warner, Wei-Jun Cai, Todd F. Melman, Kenneth D. Hoadley, D. Tye Pettay, Stephen Levas, Verena Schoepf

**S4 Table.** **Kruskal-Wallis p-values for each physiological variable.**

| **Coral species** | **calc** | **lipid** | **h_protein** | **h_carbs** | **biomass** | **PR** | **FvFm** | **cells** | **chla** | **a_protein** | **a_carbs** | **POC** |
| --- | --- | --- | --- | --- | --- | --- | --- | --- | --- | --- | --- | --- |
| *A. millepora* | **0.03** | 0.94 | **0.01** | 0.93 | 0.26 | 0.42 | **0.004** | 0.87 | 0.20 | 0.34 | **0.03** | 0.52 |
| *T. reniformis* | 0.08 | 0.20 | 0.75 | 0.34 | 0.42 | 0.63 | 0.42 | 0.08 | 0.52 | **0.02** | **0.02** | 0.87 |

Significant differences (p ≤ 0.05) are bolded. calc = calcification, chla = chlorophyll *a*, cells = endosymbiotic algal cell density, lipid = total lipids, protein = soluble animal protein concentration, carbs = carbohydrate concentration, biomass = coral ash free dry weight per area, h= host, a = endosymbiotic algae.
