# Supplementary material for: Coral physiology and microbiome dynamics under combined warming and ocean acidification
Source: PLoS One. 2018 Jan 16;13(1):e0191156. doi: 10.1371/journal.pone.0191156 (PMC5770069; doi:10.1371/journal.pone.0191156)
Supplement: S5 Table — Summary of average squared distance (Av.Sq.Dist = 29.54) in coral physiology between Acropora millepora and Turbinaria reniformis across all control and treatment coral fragments. SD = standard deviation, Av.Value = average, Contrib % = percent contribution, Cum. % = cumulative percent. (DOCX) [file pone.0191156.s007.docx]

**Supporting Information**

**Coral physiology and microbiome dynamics under combined warming and ocean acidification**

Andréa G Grottoli, Paula Dalcin Martins, Michael J. Wilkins, Michael D. Johnston, Mark E Warner, Wei-Jun Cai, Todd F. Melman, Kenneth D. Hoadley, D. Tye Pettay, Stephen Levas, Verena Schoepf

**S5 Table.** **SIMPER analysis of coral physiology.**

| **Variable** | **Group**  ***A. millepora* Av.Value** | **Group**  ***T. reniformis* Av.Value** | **Av.Sq.Dist** | **Sq.Dist/SD** | **Contrib%** | **Cum.%** |
| --- | --- | --- | --- | --- | --- | --- |
| host protein | -0.851 | 0.851 | 3.38 | 1.42 | 11.47 | 11.47 |
| algal carbohydrates | -0.794 | 0.794 | 3.03 | 1 | 10.29 | 21.77 |
| biomass | -0.701 | 0.701 | 3 | 1.05 | 10.2 | 31.97 |
| host carbohydrates | -0.625 | 0.625 | 2.73 | 1.01 | 9.28 | 41.26 |
| algal protein | -0.554 | 0.554 | 2.55 | 0.54 | 8.68 | 49.93 |
| calcification | -0.606 | 0.606 | 2.41 | 0.54 | 8.18 | 58.12 |
| FvFm | -0.569 | 0.569 | 2.3 | 0.83 | 7.82 | 65.94 |
| POC | -0.372 | 0.372 | 2.18 | 0.76 | 7.4 | 73.34 |
| lipid | 0.326 | -0.326 | 2.13 | 0.77 | 7.23 | 80.57 |
| P:R | -1.05E-03 | 1.05E-03 | 1.99 | 0.36 | 6.76 | 87.33 |
| algal cell density | -0.299 | 0.299 | 1.88 | 0.72 | 6.4 | 93.73 |

Summary of average squared distance (Av.Sq.Dist = 8.45) in coral physiology between *Acropora millepora* and *Turbinaria reniformis* across all control and treatment coral fragments. SD = standard deviation, Av.Value = average, Contrib % = percent contribution, Cum. % = cumulative percent.
